# The Intrinsic Barrier Width and Its Role in Chemical Reactivity

**DOI:** 10.1021/acscentsci.3c00926

**Published:** 2023-11-06

**Authors:** Guanqi Qiu, Peter R. Schreiner

**Affiliations:** Institute of Organic Chemistry, Justus Liebig University, Heinrich-Buff-Ring 17, 35392 Giessen, Germany

## Abstract

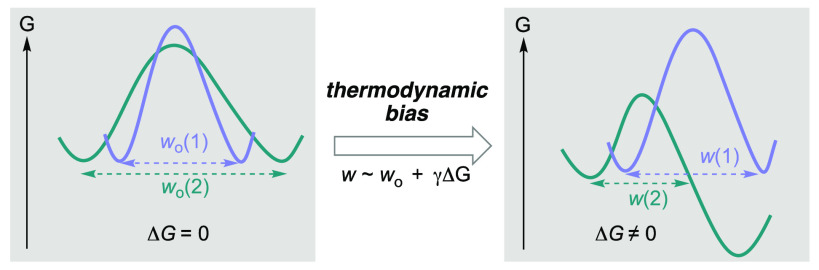

Chemical reactions are in virtually all cases understood and explained on the basis of depicting the molecular potential energy landscape, i.e., the change in atomic positions vs the free-energy change. With such landscapes, the features of the reaction barriers solely determine chemical reactivities. The Marcus dissection of the barrier height (activation energy) on such a potential into the thermodynamically independent (intrinsic) and the thermodynamically dependent (Bell–Evans–Polanyi) contributions successfully models the interplay of reaction rate and driving force. This has led to the well-known and ubiquitously used reactivity paradigm of “kinetic versus thermodynamic control”. However, an analogous dissection concept regarding the barrier width is absent. Here we define and outline the concept of intrinsic barrier width and the driving force effect on the barrier width and report experimental as well as theoretical studies to demonstrate their distinct roles. We present the idea of changing the barrier widths of conformational isomerizations of some simple aromatic carboxylic acids as models and use quantum mechanical tunneling (QMT) half-lives as a read-out for these changes because QMT is particularly sensitive to barrier widths. We demonstrate the distinct roles of the intrinsic and the thermodynamic contributions of the barrier width on QMT half-lives. This sheds light on resolving conflicting trends in chemical reactivities where barrier widths are relevant and allows us to draw some important conclusions about the general relevance of barrier widths, their qualitative definition, and the consequences for more complete descriptions of chemical reactions.

## Abstract 2

We demonstrate that like the reaction barrier height, the barrier width also consists of thermodynamically independent and dependent components.

## Introduction

Chemists are well versed in describing reactions pictorially and rigorously through reaction rates (kinetics) and driving forces (thermodynamics) in terms of the relative positions of the involved molecules with respect to their (Gibbs) energy. This is particularly true for barrier heights of transition structures, but the consideration of barrier widths is virtually nonexistent. That is, the intrinsic reaction coordinate,^1^ typically defined as a one-dimensional parameter, is considered to be more of a complement than a variable. Even the typical and often ignored unit for the reaction coordinate that may be composed of atomic momenta, distances, and angles does not always reveal an immediate meaning to the practicing chemist. It is somewhat astonishing to see that IUPAC notes that “’Reaction coordinate’ is sometimes used as an undefined label for the horizontal axis of a potential-energy profile or a Gibbs energy diagram”.^2^ However, the barrier width, displayed prominently on the *x* axis of such a diagram, is key when it comes to quantum mechanical tunneling (QMT) as it linearly affects the tunneling rate.^3,4^ Eyring’s semiclassical theory of the “activated complex” mentions QMT only in passing as “Tunneling may occasionally play some role in the motion”.^5^ Similarly, Evans and Polanyi simply state that “For light masses, such as hydrogen and deuterium, the statistical probability must be calculated according to the principles outlined by Wigner [···] which will result in the appearance of tunnelling effects”, but neither publication mentions the term “barrier width”.^6^ Of course, modern developments of transition state theory (TST) take tunneling fully into account,^7−9^ but in contrast to the notion of barrier heights, barrier widths play essentially no role in the qualitative description of chemical reactions. As QMT has been recognized as being more common than typically assumed,^10^ one cannot argue that it is likely to be of minor importance for typical chemical reactions.^11−24^ In particular, the barrier width reduction was found to be directly linked to the increase in the rates of QMT processes.^12^ Therefore, just as there is a systematic modulation of the barrier height, the barrier width also deserves a similarly systematic treatment in order to acquire a holistic understanding of chemical reactivity.

Here we present the idea and first results of distinctively changing the thermodynamically independent and the thermodynamically dependent components of the barrier widths of a chemical reaction using the very sensitive QMT half-lives as a read-out. As a system to analyze this cleanly, we chose the simple conformational isomerizations of substituted benzoic acid derivatives. This allows us to conceptualize the qualitative definition of barrier width based on an intrinsic barrier width, draw important conclusions about the consequences for more holistic descriptions of chemical reactions on the basis of one-dimensional reaction coordinates, and examine the general relevance of barrier widths in chemical reactions.

We approach our analysis from Marcus theory^25−34^ because it dissects the Gibbs energy of activation Δ^⧧^*G* into intrinsic (the intrinsic barrier Δ^⧧^*G*_0_, which corresponds to Δ^⧧^*G* for Gibbs reaction energy Δ*G* = 0) and thermodynamic (the effect of Δ*G* on top of Δ^⧧^*G*_0_) contributions. The Marcus dissection was quantitatively expressed in the linear approximation Δ^⧧^*G* = Δ^⧧^*G*_0_ + αΔ*G* as the Leffler equation.^35^ The thermodynamic contribution had already been captured by the Bell–Evans–Polanyi (BEP) principle that describes a linear correlation of reaction rate constants (or other activation parameters) with Δ*G*.^36,37^ The BEP principle is applicable to the same family of reactions in which the change in reaction barrier is affected only by the driving force change (ΔΔ*G*) but is otherwise agnostic to the causes of the thermodynamic change; this relates qualitatively to the Hammond postulate.^38^ The thermodynamically independent contribution, the intrinsic barrier, thereby reflects the reorganization energy that is required for the change in the nuclear coordinates of the reactant state to that of the product state at zero driving force.

**Figure 1:**
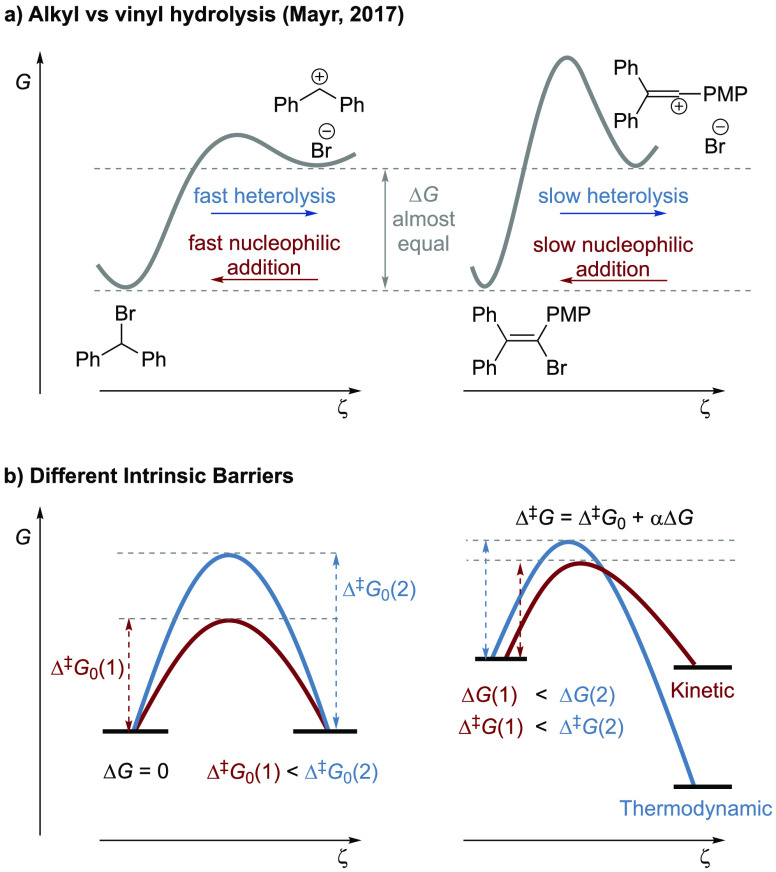
Importance of the intrinsic barrier: (a) Given equal thermodynamic driving force, a higher intrinsic barrier slows both the forward reaction, heterolysis, and its microscopically reverse reaction, nucleophilic addition. (b) The principle of kinetic versus thermodynamic control is a general phenomenological consequence of Marcus dissection: due to its lower intrinsic barrier, the actual barrier of the kinetic path is lower despite its thermodynamic disadvantage.

The joint description of reactivity using intrinsic barriers and the BEP principle can explain a great number of reactivity patterns and trends.^31,33,39−43^ For example, Mayr reported that, given an equal thermodynamic driving force compared to that of benzhydrylium ions, vinyl cations react more slowly with nucleophiles and form less readily via heterolysis (Figure 1a).^40^ This is a manifestation of different intrinsic barriers that were attributed to differences in rehybridization energies. A crucial general phenomenological consequence of Marcus dissection is the well-known principle of “kinetic versus thermodynamic control”^44^ (Figure 1b, right): due to its lower intrinsic barrier, the Gibbs energy of activation for the thermodynamically less favored red reaction is still lower than that of the blue reaction, and kinetic control ensues.

Remarkably, concepts analogous to Marcus’ dissection for barrier widths are absent. This is most notable for, but not limited to, QMT reactivity.^11,16,18,45,46^ When QMT is taken into the reactivity picture, two components should then be defined: the intrinsic barrier width and the thermodynamic driving force effect on the barrier width, i.e., the barrier width counterpart of intrinsic reactivity and the BEP principle. Support for the concept of the intrinsic barrier width and thermodynamic driving force comes from many examples reporting that while QMT reactivity is strongly affected by the thermodynamic driving force, substituents, and matrix environments, the trends are often conflicting.^34,47−50^ Resolving these conflicts, and thus formulating predictive reactivity models, will be valuable for us to bring about a deep and detailed understanding of a variety of reactions that prove to be sensitive to changes in barrier width.

Analogous to intrinsic barrier heights, physically, intrinsic barrier widths reflect the reorganization of the nuclear coordinates that is required for the deformation of the geometry of the reactant state to that of the product state at zero driving force. Herein we define and explain this original concept and report experimental and theoretical studies to demonstrate the distinct roles of intrinsic barrier width and driving force effects on the barrier width to develop a unified paradigm (Figure 3). Such a unified reactivity theory includes both the competition between thermal and tunneling processes^51−53^ and the interplay between intrinsic reactivity and thermodynamic BEP contributions.

**Figure 2:**
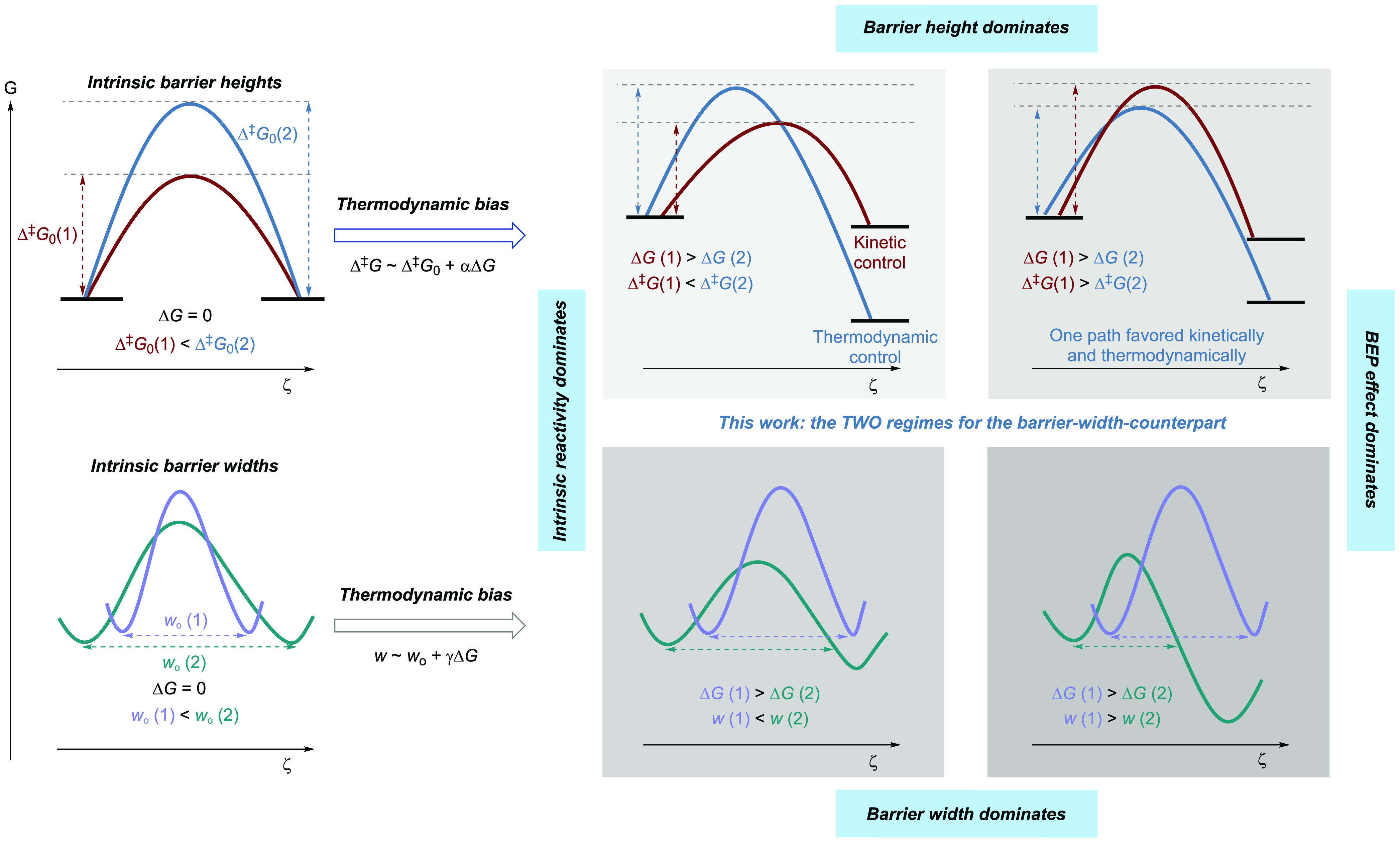
Considerations of potential energy hypersurfaces. Pictorial presentation of the formulation of a unified reactivity paradigm that dissects and combines, compares, and contrasts over-the-barrier thermal reactions and intrinsic and thermodynamic contributions to the overall reactivity. The intrinsic barrier height reflects the reorganization energy, whereas the intrinsic barrier width reflects the reorganization distance. As the barrier width is most significantly (though not exclusively) represented by QMT, we take the barrier width as the reorganization distance over which the wave functions of the reactant state extend into the classically forbidden region under the barrier.

Reaction selectivity involves various competing aspects: Is the reactivity dominated by the barrier height or the barrier width? For each, is the path with higher intrinsic reactivity or the thermodynamically more exergonic path favored? Would each path respond to the thermodynamic changes differently? The four quadrants outlined in Figure 2 capture these reactivity paradigms. We outline here the intrinsic relationship between barrier characteristics, including height and width, and the thermodynamic driving force. This work brings the barrier width into focus as exemplified by, but not limited to, the modulation of QMT reactivity using benzoic acid derivatives and their conformational isomerization as an example.

## Results and Discussion

### Definition and Concept

**Figure 3:**
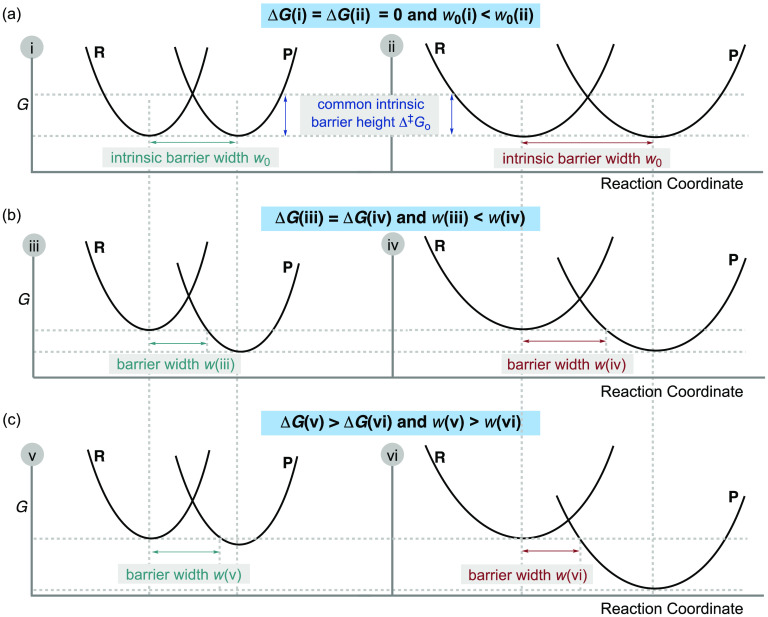
Definition and demonstration of the concept of intrinsic barrier width. Marcus-type analysis of the barrier widths. Left-hand-side reactions (i), (iii), and (v) have the same intrinsic barrier width, whereas right-hand-side reactions (ii), (iv), and (vi) all have a different common intrinsic barrier width. (a) The two reactions have the same intrinsic barrier height but different intrinsic barrier widths. (b) The barrier widths of the two reactions respond to the same thermodynamic driving force change at different sensitivities. (c) Because of a large thermodynamic bias, reaction (vi) has a greater intrinsic barrier width but a smaller actual barrier width than reaction (v).

At the start, we define the intrinsic barrier width (*w*_0_) as the barrier width at zero driving force (Figure 3a). To the best of our knowledge, the “intrinsic barrier width” has been named in only one study^54^ and has never been systematized in chemical reactivity. Marcus theory hereby assists in our reasoning, as it, as well as other related concepts, considers a reactant (R) and product (P) nestling in a parabolic bowl, and the transition state is approximated as the point of intersection of the two bowls. For simplicity, the reactant and product states are assumed to have the same nuclear vibrational force constants (an assumption that is silently made also for the BEP principle and the Hammond postulate), and the zero-point vibrational energy (ZPVE) is omitted. In Figure 3, all three reactions on the left-hand side in a, b, and c are associated with the same intrinsic barrier width (*w*). The three reactions on the right-hand side also have the same intrinsic barrier width, which is larger than that on the left-hand side. The two reactions (i) and (ii) experience equal barrier heights at zero driving force, i.e., the same intrinsic barrier height. The stiffness of the parabolae reflects the energy associated with displacement from the equilibrium nuclei coordinate along the one-dimensional reaction profile, which we employ here for simplicity. Reaction (i) has a smaller intrinsic barrier width, indicating a higher intrinsic QMT rate constant than for reaction (ii). Comparing reactions (iii) and (iv), reaction (iv) involves flatter parabolae than reaction (iii), where the change in the barrier width is more sensitive to the thermodynamic driving force change than in reaction (iii). Hence, a sufficiently large thermodynamic driving force is likely to lead to narrowing of the actual barrier. This is shown in reactions (v) and (vi): reaction (vi) has a larger intrinsic barrier width than (v) but a higher thermodynamic driving force that significantly decreases the barrier width *w*(vi). Therefore, thermodynamic bias can reverse the QMT reactivity trend set by the relative intrinsic barrier width; i.e., the greater intrinsic barrier width could end up with the smaller actual barrier width. Note that barrier width is relevant not only in QMT reactions but also for others such as over-the-barrier dynamic reactivities. Examples include nonstatistical internal energy redistributions and post-transition-state bifurcations, in which the propagating trajectories along the reaction energy surface, and thus the reaction barrier shapes, are crucial to the reactivity.^55−60^

**Figure 4:**
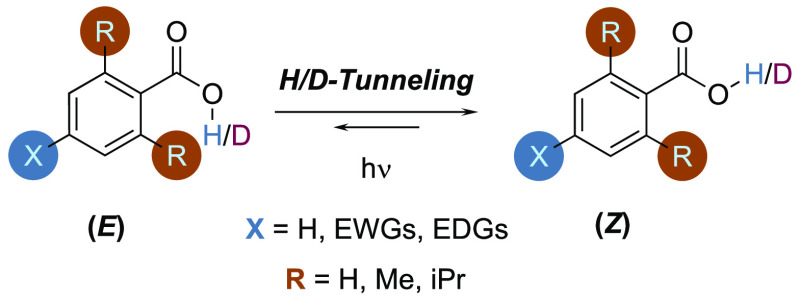
Studied model system. Benzoic acid derivatives were employed to evaluate the effects of steric hindrance and electronic properties on the *E/Z*-equilibration QMT behavior. These effects help in evaluating the influence of the barrier width on the QMT back reaction of the (*E*)- to the (*Z*)-isomer. The (*E*)-isomer is populated photochemically.

As a model system, we chose to study the *E* ⇄ *Z* conformational isomerization of benzoic acid derivatives (Figure 4) because (1) the reaction coordinate is well represented by H-atom movements and (2) these compounds lend themselves very well to the separation of electronic (far from the primary reaction sphere via *para-*substituent X) and steric effects (change in the direct vicinity around the reaction center via *ortho-*substituents R). By changing X, the electronic density at the carboxylic carbon can be varied with negligible disturbance to the geometry at this site (represented by the vertical displacement of Marcus parabolae in Figure 3).^61^ In contrast, substituents R introduce mostly steric interactions in close proximity to the carboxylic acid, thereby changing the reorganization path (represented by changes in the horizontal displacement or stiffness of Marcus parabolae; compare Figure 3(iii)(iv)). To limit electronic effects transmitted to the R groups, we restrict our analysis to R = H, Me, *i*Pr.

The *E*/*Z*-conformers of carboxylic acids interconvert through C–O bond rotations. Stabilized by an intramolecular hydrogen bond, the (*Z*)-isomer is effectively the only observable conformer under ambient conditions.^62−64^ The higher-lying (*E*)-isomer can be accessed photochemically by photoirradiation of the (*Z*)-isomer and can be trapped in various inert matrices at cryogenic temperatures.^65−69^ In our previous studies on the conformational isomerization of *para*-substituted benzoic acid derivatives^61^ the ^1^H-(*E*)-conformers could not be observed because of fast H-tunneling to the more stable (*Z*)-conformers for a variety of *para*-substituted derivatives. The carboxylic acid moiety must be deuterated (to form the respective ^2^H-(*E*)-conformers) to attain measurable kinetics, manifesting a large kinetic isotope effect (KIE). The rate constants (∼10^–3^ s^–1^ in the Ar matrix at 11 K) of the *E* → *Z* isomerizations are impossibly high for an over-the-barrier process at cryogenic temperatures, at which only the vibrational ground state is populated. The rates of *E* → *Z* isomerizations were found to be temperature-independent within the 11–20 K temperature range. All of these observations strongly support a QMT process. We have preliminarily demonstrated that electron-donating groups (EDGs) and electron-withdrawing groups (EWGs) at the *para*-position systematically change the barrier widths (determined by computing the intrinsic reaction coordinates (IRCs) and that the experimental QMT rate constants correlate strongly with the computed barrier widths.^61^

The important question concerns the origin of the rate changes (whether it is the intrinsic barrier width or the BEP effect) in QMT reactivity upon substitution. As a reaction barrier describes the energy of a collection of atoms in terms of the position of atoms, we expect that factors affecting the intrinsic barrier height are also able to affect the intrinsic barrier width: together, they constitute the “intrinsic barrier shape”. To this end, we opted for three sets of benzoic acid derivatives, each having an alkyl R substituent at the *ortho*-positions, which introduces steric interactions with the acid group’s conformational isomerization, thereby altering the barrier width. The electronic effects are limited to moderate electron donation (+I) for R = Me or *i*Pr. For each set, the *para*-substituent is varied to generate the respective linear Gibbs energy relationships (LFER) for the barrier width and QMT rate constant. As the barrier width is related to the distance the participating atoms must move, steric interactions are expected to result in different intrinsic barrier widths and/or different sensitivities of the thermodynamic driving force to barrier width. For example, we expect in the case of steric hindrance through substitution at the *ortho*-position to change how much the carboxylic acid moiety deviates from coplanarity with the arene.^35^ Therefore, we expect three nonoverlayed LFER lines for the three sets (unless some coincide due to the cancellation of differences).

### Computational Predictions

The tunneling barrier widths were first analyzed through computations of the intrinsic reaction coordinates (IRCs) connecting the rotamerization transition structures with the (*E*)- and (*Z*)-conformers at the MP2/cc-pVDZ level of theory. A final potential energy curve along the isomerization IRC was then constructed from MP2/cc-pVDZ energy points and ZPVEs of the vibrational “reaction” mode of the (*E*)-isomer (typically around 500 cm^–1^ for ^1^H (OH) acid and 400 cm^–1^ for ^2^H (OD) acid) toward the transition structure (details in the SI). The tunneling path is assumed to be one-dimensional and to go through the Gibbs energy barrier of the conformational isomerization. The MP2/cc-pVDZ level of theory has been chosen based on the comparison of single points for (*Z*)-benzoic acid derived from various levels of theories with those obtained at CCSD(T)/cc-pVTZ from our previous study.^61^ MP2 relative energies are the closest to the CCSD(T)/cc-pVTZ and are better than those of B3LYP and M06–2X with the same basis set.

**Figure 5:**
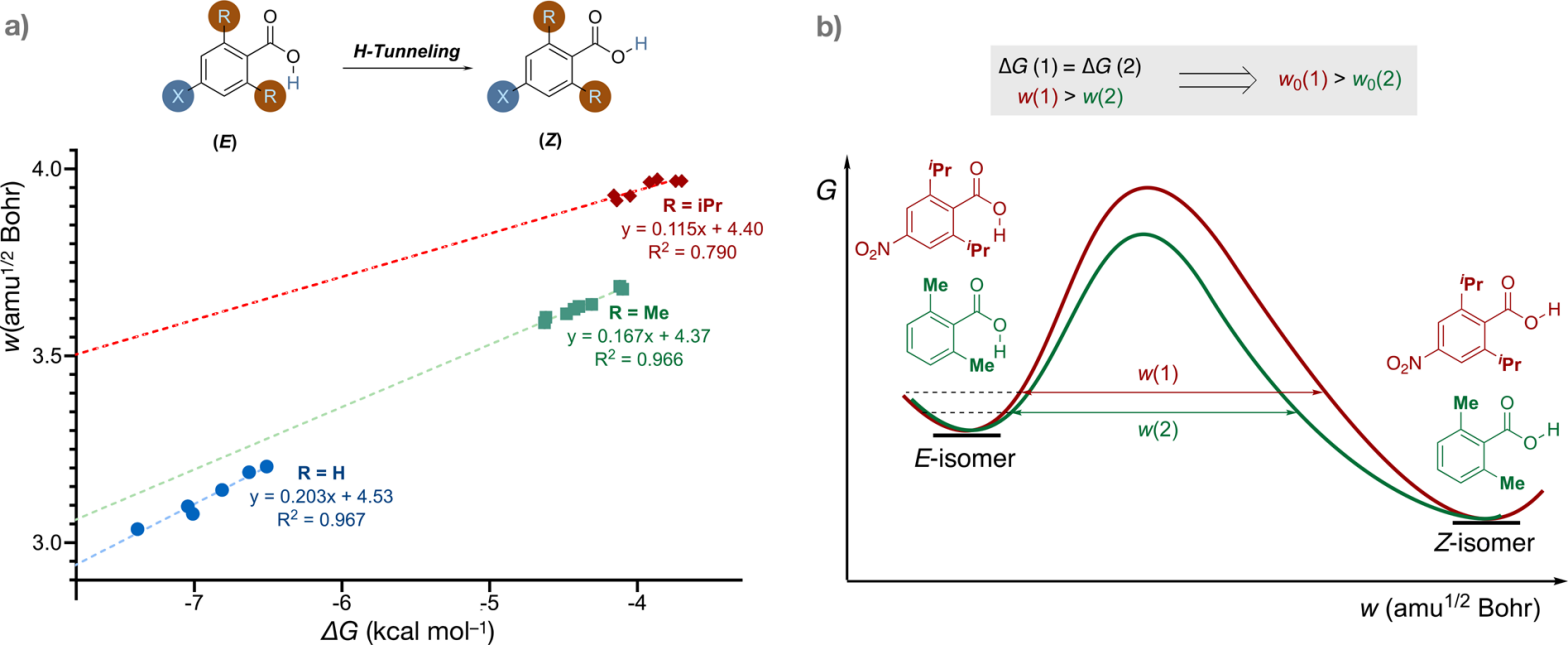
Computational predictions. (a) BEP correlations for three series of different *ortho*-substituents manifesting three different intrinsic barrier widths. All computations were performed at MP2/cc-pVDZ. The vertical axis, the “barrier width”, shows the mass-weighted Cartesian coordinates in units of amu^1/2^ Bohr along the path for R = H, X = CN, Cl, F, CH_2_F, H, Me; for R = Me, X = NO_2_, CN, AcNMe, Cl, F, CCH, H, Me; and for R = *i*Pr, X = CN, NO_2_, CF_3_, Cl, F, H, Me, all in ascending order of the Gibbs energy change of isomerization (i.e., these are ordered as above left to right). (b) Qualitative IRCs of two reactions signifying different intrinsic barrier widths: with the same thermodynamic driving force, different barrier widths are the result of the different intrinsic barrier widths.

Figure 5 shows a plot of the Gibbs energy change of the conformational (−O^1^H) isomerization against the barrier width for each of the three series with different *ortho*-substituents. Selected computed half-lives are summarized in Table 1 (CVT/SCT//MP2/cc-pVDZ). The *para*-substituents were varied so that different series covered comparable ranges of the conformational Gibbs energy change. As expected, there are three nonoverlayed LFER lines (compare entries 1 and 2 as well as 3–5 in Table 1). Within each series, the intrinsic barrier width is fairly constant, and the variation of the *para*-substituent can be represented by the vertical displacement of the Marcus parabolae without changing the shape or the horizontal displacement (Figure 3). The slopes and intercepts are different for different *ortho*-substituents, i.e., the intrinsic barrier widths are all different among the three series, represented by the horizontal displacement and/or the stiffness of Marcus parabolae described in Figure 3. *ortho*-Substituents change the intrinsic barrier width, as they disturb the intrinsic barrier shape of the isomerization.

**Table 1:** Selected Computed Half-Lives from the Reactions in Figure 6 (CVT/SCT//MP2/cc-pVDZ)

**Entry**	**R =**	**X =**	**Δ*****G*****(kcal mol**^**–1**^**)**	*t*_1/2(comp)_ (min)
**1**	H	H	–6.6	2.0 × 10^–6^
**2**	H	CN	–7.4	1.3 × 10^–7^
**3**	Me	H	–4.2	4.9 × 10^–2^
**4**	Me	Cl	–4.4	2.1 × 10^–2^
**5**	Me	CN	–4.6	4.7 × 10^–3^

The *ortho*-Me series has a moderately smaller intrinsic barrier width than the *ortho*-H series, as the former’s correlation line has a smaller vertical intercept. On top of that, the EDG *ortho*-Me substituent decreases the exergonicity, leading to the *ortho*-Me series being less exergonic than the *ortho*-H series. As a result, despite the smaller intrinsic barrier width, the thermodynamic contribution to the barrier width outcompetes the intrinsic barrier width effect such that the resultant barrier width for the *ortho*-Me series is larger than that of the *ortho*-H series (represented in Figure 3c). For example, in a comparison of entries 1 and 3 in Table 1, entry 1 is both more exergonic and is predicted to react faster than entry 3. The relative QMT reactivity between the *ortho*-Me series and the *ortho*-H series is dictated by the BEP effect, belonging to the bottom-right quadrant in Figure 2.

The EDG *ortho*-*i*Pr substituent decreases the exergonicity slightly more than the *ortho*-Me substituent, with an overlap in the range of thermodynamic driving forces between the two series. However, the difference in the intrinsic barrier width is significant, leading to a resultant barrier width for the *ortho*-*i*Pr series being considerably larger than that of the *ortho*-Me series.

**Figure 6:**
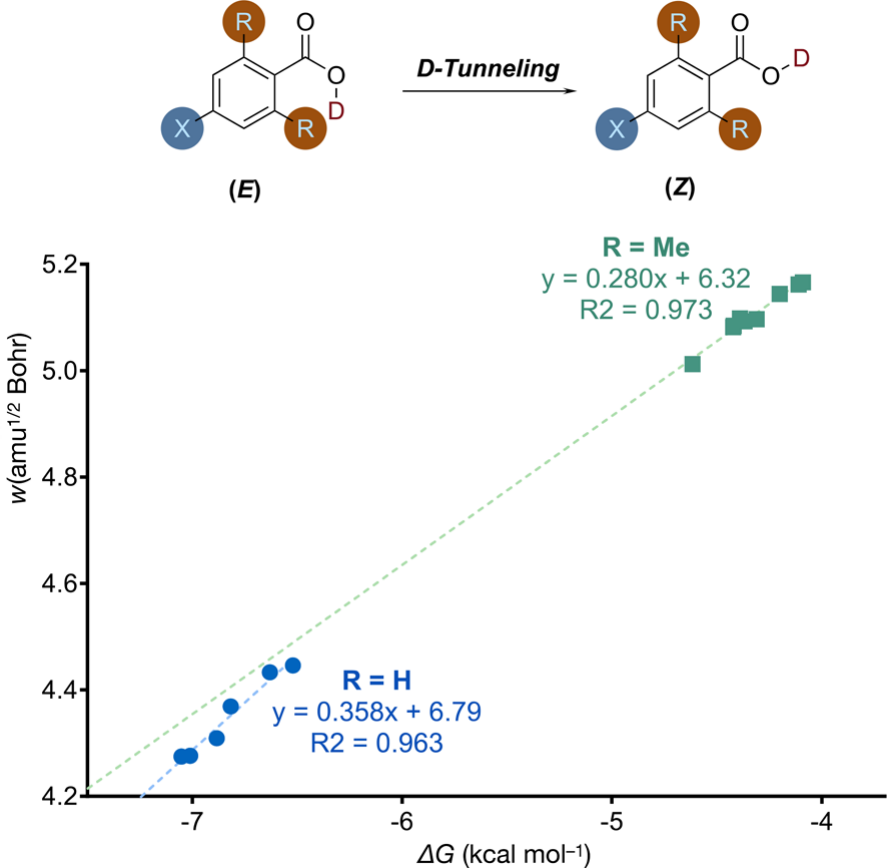
BEP correlations. We show two series of deuterated carboxylic acids of different *ortho*-substituents—R = H, X= Cl, F, CCH, CH_2_F, H, Me and R = Me, X= CN, Br, Cl, F, CHO, CCH, NMe_2_, H, Me—all in ascending order of the Gibbs energy change of isomerization (i.e., from left to right). All computations were performed at MP2/cc-pVDZ.

As an alternative to *ortho*-substitution, one could introduce isotopic substitution that changes the intrinsic barrier width by cutting through the reaction barrier at a different ZPVE for reactions at the ground vibrational level. We studied the (−O^2^H) deuterated *ortho*-H and Me carboxylic acids series (ArCOOD) with the same computational method (Figure 6). This again results in two different BEP correlations with different slopes and vertical intercepts. We compare, in particular, the ArCOOH and ArCOOD *ortho*-Me series for which we find clear isotope effects in both the driving force sensitivity and intercept in the BEP correlation with the barrier width. As the Cartesian reaction coordinate is mass-weighted, the mass effect of isotopic substitution is mapped into the barrier width. Therefore, isotopic substitution changes the intrinsic barrier width mostly not via altering the intrinsic barrier shape as a whole but by cutting through the reaction barrier at a different ZPVE.

### Experimental Validation

The QMT kinetics of the conformational isomerizations were studied via matrix-isolation techniques. In a typical kinetic experiment, the more stable (*Z*)-isomer of the aryl carboxylic acid was deposited on a CsI window in an Ar matrix at 11 K. The higher-lying (*E*)-isomer was generated photochemically by irradiation of the (*Z*)-isomer at 254 nm. The C=O stretching characteristic IR bands were quantitatively monitored to determine the *E* → *Z* isomerization rate constants (details in the SI). In general, the C=O stretching band position is around 1780 cm^–1^ in the (*E*)-isomer and around 1740 cm^–1^ in the (*Z*)-isomer. As before, the ^1^H-(*E*)-conformer could not be observed because of fast H-tunneling to the more stable (*Z*)-conformer (see Table 1 for the computed half-lives). Thus, matrix isolation kinetic measurements were performed for the deuterated forms of all aryl carboxylic acids.

**Figure 7:**
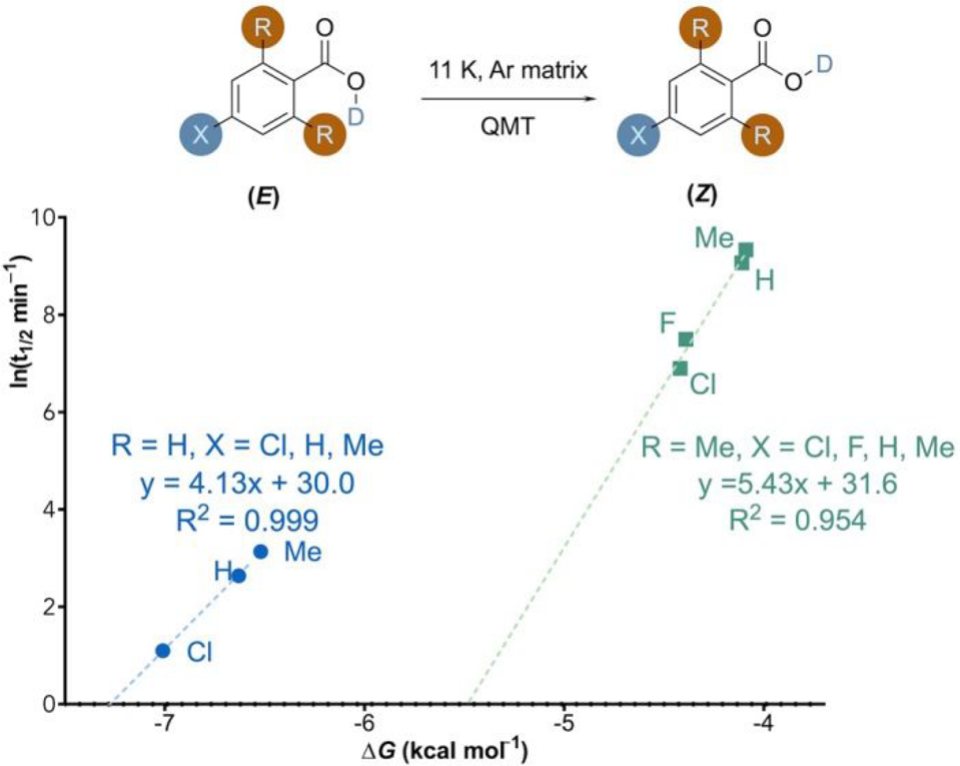
Comparison of tunneling half-lives and Gibbs energy change. Plot of experimental QMT ln(*t*_1/2_) in min against the Gibbs energy change of *E* → *Z* rotamerization in the Ar matrix at 11 K. For R = H, X = Cl, H, Me and for R = Me and X = Cl, F, H, Me, all in ascending order of the Gibbs energy change of isomerization. The Δ*G* values were computed at MP2/cc-pVDZ.

Table 2 summarizes the experimental kinetic measurements for the *E* → *Z* rotamerizations in Ar matrices at 11 K. The experimental entries are divided into two groups, each of which has a distinct *ortho*-substituent: H (entries 1–3) and Me (entries 4–7). The temperature independence of the half-lives at 11 and 20 K and the very large primary H/D KIE (the computed KIEs are on the order of 10^6^), whereas the experimental large KIE is suggested by the undetectability of the higher-lying protium (*E*)-conformer upon photoexcitation, support the notion of a QMT mechanism. For each particular *ortho*-substituent, the electronic *para*-substituent was varied and the isomerization kinetics of the *para*-substituted deuterated aryl acids were systematically studied to derive the respective BEP correlation for the series of each *ortho*-substituent. Figure 7 shows the BEP correlation for each series. Clearly, the experimental kinetics measurements also lead to two distinct BEP correlation lines for the *ortho*-H and *ortho*-Me series. Both the slopes and intercepts are significantly different between the *ortho*-H and *ortho*-Me series.

**Table 2:** Computed Half-Lives *t*_1/2(comp)_ of the Reactions Depicted in Figure 7 (at CVT/SCT//MP2/cc-pVDZ) and Experimental Half-Lives (*t*_1/2(exp)_) for the *E* → *Z* Rotamerization in the Ar Matrix at 11 K

**Entry**	**R =**	**X =**	**Δ*****G*****(kcal mol**^**–1**^**)**^a^	***t***_1/2**(comp)**_**(min)**	***t***_1/2**(exp)**_**(min)**
**1**	H	H	–6.6	0.036	14
**2**	H	Me	–6.5	0.047	23
**3**	H	Cl	–7.0	0.006	3
**4**	Me	H	–4.1	38100	8660
**5**	Me	Me	–4.1	53000	13300
**6**	Me	Cl	–4.4	17600	1600
**7**	Me	F	–4.4	18000	2800

a The Gibbs energies of isomerization at 11 K are about the same at 298 K.

Qualitatively, the computed half-lives (*t*_1/2(comp)_) reproduce the trend for both the *ortho*-H and *ortho*-Me series. Within each series, the more exergonic the reaction is, the faster the reaction is predicted to be. The predicted half-lives are systematically lower (by about two orders of magnitude) in the *ortho*-H series but systematically higher (by up to one order of magnitude) in the *ortho*-Me series than the experimental half-lives. For the *ortho*-H series, the computed rate is more sensitive to the thermodynamic driving force than is the experimental rate. However, for the *ortho*-Me series, the computed rate is less sensitive to the thermodynamic driving force than is the experimental rate. These disparities are possibly due to the solvent effects of the Ar matrix host.^34^ More importantly, these disparities suggest that the solvent effects are also likely to consist of both intrinsic and thermodynamic components, making the *ortho*-H and *ortho*-Me series respond differently.

**Figure 8:**
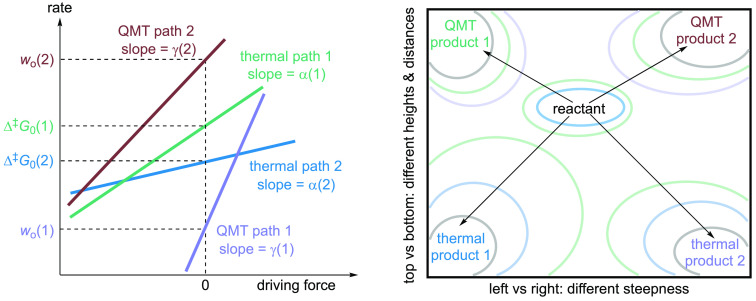
Left: Rate-driving force relationships in thermal and QMT reactions, with different slopes and intercepts, i.e., various intrinsic and thermodynamic contributions to the barrier height and the barrier width. The slopes and intercepts are arbitrarily assigned. Right: Multidimensional reaction energy contour.

In this study, we assumed that QMT processes happen through the same reaction coordinate as the over-the-barrier thermal reaction in one dimension. For such a simple conformational isomerization at cryogenic temperatures, we expect this assumption to be reasonable. However, in general, chemical reactions are multidimensional and the most favored QMT path may not necessarily proceed through the reactional barrier of the most favored thermal reaction path. The path with the highest intrinsic thermal reactivity, the path with the greatest thermodynamic contribution to the thermal reactivity, the path with the highest intrinsic QMT reactivity, and the path with the greatest thermodynamic contribution to the QMT reactivity could all lead to different products rather than coincide (Figure 8). The reactivities of all four paths could respond to changes in the thermodynamic driving force differently, making the dominance of each of the four paths possible.

## Conclusions

In this work, we define the intrinsic barrier width by analogy to the well-established instrinsic barrier height. As we demonstrate, both affect the rates of chemical reactions, and both need to be taken into account for a deep understanding of chemical reactivity. Our concept to include the notion of barrier widths uses the ideas of Marcus dissection to arrive at an intuitive picture that uses the moving of parabolae to construct different scenarios for the shapes of one-dimensional potential energy hypersurfaces.

We use simple QMT rotamerizations of substituted benzoic acids as our “read-out” to uncover the effects of barrier width because QMT is highly sensitive to even minute changes. Deconvolution analyses of intrinsic barrier widths and thermodynamic effects on the barrier width hence delineate the ramifications of chemical reactivity. By considering the control of chemical reactions with over-the-barrier thermal reactions along with QMT reactivity, one can devise a unified reactivity paradigm consisting of four elements: intrinsic barrier height, thermodynamic modification to the barrier height, intrinsic barrier width, and thermodynamic modification to the barrier width.

The immediate application and future challenge will be controlling barrier widths, which has, in contrast to changing barrier heights (generally practiced in catalysis), not been established at all. This may be achieved, for example, with further progress in the fields of external electric field catalysis^70−73^ (which could help tune the intrinsic barrier width) and strong vibrational coupling^74,75^ (which could help tune the driving force) to develop more selective and unprecedented chemical reactions.

## Data Availability

Metadata can be accessed at 10.22029/jlupub-11394

## Supporting Information Available

Experimental procedures, kinetic data, and computations of Gibbs free energies (PDF)
Transparent Peer Review report available (PDF)
