# Supplementary material for: The Intrinsic Barrier Width and Its Role in Chemical Reactivity
Source: ACS Cent Sci. 2023 Nov 6;9(11):2129–37. doi: 10.1021/acscentsci.3c00926 (PMC10683502; doi:10.1021/acscentsci.3c00926)
Supplement: Supplementary file 1 — oc3c00926_si_001.pdf [file oc3c00926_si_001.pdf]

The Intrinsic Barrier Width and its Role in Chemical Reactivity

Guanqi Qiu* and Peter R. Schreiner*

Institute of Organic Chemistry, Justus Liebig University, Heinrich-Buff-Ring 17, 35392 Giessen, Germany

*Authors to whom correspondence should be addressed; Email: Guanqi.Qiu@org.chemie.uni-giessen.de, prs@uni-giessen.de

Supporting Information

Table of Contents

1. Cartesian Coordinates of Computationally Optimized Geometries S2

2. Tunneling Computations.. S68

3.Computed Linear Free Energy Relationships S80

4. Computed and Matrix IR Spectra.. S84

5. Matrix Kinetic Measurements S99

References S106

1. Cartesian Coordinates of Computationally Optimized Geometries

Atomic cartesian coordinates [Å] of benzoic acid derivatives were optimized at the MP2/cc-pVDZ level of theory as implemented in Gaussian 16.[1] This choice was made based on the comparison of energy single points for *Z*-benzoic acid derived from various levels of theory with those obtained at CCSD(T)/cc-pVTZ from our previous study.[2] MP2/cc-pVDZ energies are the closest to CCSD(T)/cc-pVTZ and are better than B3LYP/cc-pVDZ and M06-2X/cc-pVDZ.

Optimized structures, electronic energies, and zero-point vibrational energies (ZPVE) are provided. For transition states, the imaginary frequency is given.

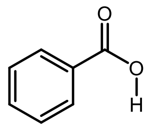

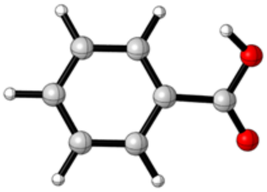

***E*-1a**

6	-1.887838000	-1.196161000	0.145423000
6	-0.486404000	-1.224983000	0.121509000
6	0.241804000	-0.026138000	-0.007883000
6	-0.445650000	1.196516000	-0.152074000
6	-1.850185000	1.220018000	-0.141478000
1	-2.450089000	-2.128843000	0.258727000
1	0.066195000	-2.165262000	0.205201000
1	0.102075000	2.131965000	-0.316342000
1	-2.379345000	2.170529000	-0.264690000
6	1.743833000	-0.121196000	-0.032888000
8	2.340949000	-1.136182000	-0.317100000
8	2.420672000	1.017907000	0.285710000
1	1.780736000	1.647868000	0.649580000
6	-2.573042000	0.025895000	0.015682000
1	-3.667647000	0.046240000	0.028900000

E = –419.618700 au   ZPVE = 0.115519 au

For deuterated acid (***E*-5a**): ZPVE = 0.112060175 au

***TS*-1a**

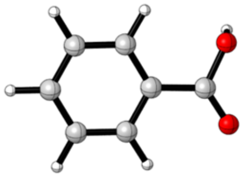

6	1.917806000	1.183191000	-0.050175000
6	0.517359000	1.236958000	-0.035482000
6	-0.231406000	0.043092000	0.010649000
6	0.427152000	-1.201812000	0.054143000
6	1.830447000	-1.248549000	0.042496000
1	2.499251000	2.110223000	-0.089022000
1	-0.015658000	2.191905000	-0.060439000

1	-0.162545000	-2.120095000	0.111328000
1	2.343156000	-2.215354000	0.080257000
6	-1.725319000	0.143275000	0.021973000
8	-2.328250000	1.191255000	0.086624000
8	-2.387811000	-1.080893000	0.003015000
1	-2.488677000	-1.330798000	-0.928012000
6	2.577485000	-0.059340000	-0.011920000
1	3.671818000	-0.099668000	-0.021323000

E = –419.609303 au ZPVE = 0.114051 au v_{i} = 563.02i cm^{-1}For deuterated acid (***TS*-5a**): ZPVE = 0.110967219 au v_{i} = 394.28i cm^{-1}

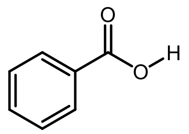

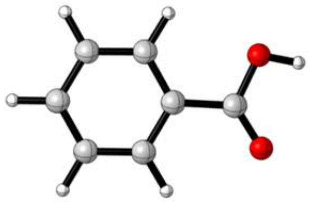

***Z*-1a**

6	1.909138000	1.194908000	0.000277000
6	0.507344000	1.230532000	0.000227000
6	-0.223538000	0.026826000	0.000022000
6	0.447399000	-1.211868000	-0.000229000
6	1.850926000	-1.239124000	-0.000250000
1	2.478733000	2.130042000	0.000520000
1	-0.039617000	2.177765000	0.000355000
1	-0.129075000	-2.140318000	-0.000299000
1	2.375667000	-2.200185000	-0.000592000
6	-1.715178000	0.120914000	-0.000044000
8	-2.345271000	1.162627000	-0.000602000
8	-2.311934000	-1.099674000	0.000579000
1	-3.265625000	-0.902027000	0.000319000
6	2.583748000	-0.039314000	-0.000026000
1	3.678531000	-0.066158000	0.000018000

E = –419.629553 au ZPVE = 0.115807 au
For deuterated acid (***Z*-5a**): ZPVE = 0.112342502 au

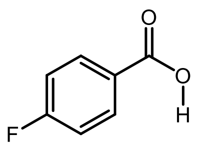

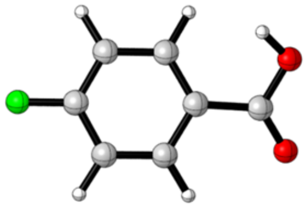

***E*-1b**

6	-1.446013000	-1.215298000	0.135566000
6	-0.046196000	-1.228168000	0.116899000
6	0.678122000	-0.025869000	-0.005803000
6	-0.018455000	1.191277000	-0.150489000
6	-1.421492000	1.213208000	-0.145946000

1	-2.027187000	-2.135601000	0.241826000
1	0.507548000	-2.167491000	0.199943000
1	0.519646000	2.132458000	-0.310490000
1	-1.980939000	2.144757000	-0.267933000
6	2.179233000	-0.115369000	-0.029808000
8	2.778546000	-1.130895000	-0.306929000
8	2.853222000	1.028080000	0.278947000
1	2.216269000	1.656251000	0.650736000
6	-2.114432000	0.008297000	0.005100000
9	-3.461565000	0.024852000	0.017407000

E = –518.651803 au   ZPVE = 0.107515 au
For deuterated acid (***E*-5b**): ZPVE = 0.104060717 au

***TS*-1b**

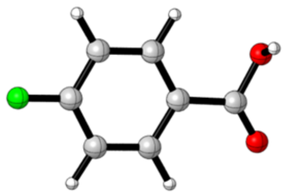

6	1.467389000	1.218437000	0.045646000
6	0.068152000	1.248317000	0.034234000
6	-0.669050000	0.047049000	-0.010305000
6	0.006566000	-1.188617000	-0.057226000
6	1.408271000	-1.225849000	-0.049291000
1	2.062182000	2.135411000	0.082949000
1	-0.472075000	2.198834000	0.060855000
1	-0.568503000	-2.115762000	-0.114778000
1	1.957751000	-2.170532000	-0.089265000
6	-2.162149000	0.130052000	-0.020065000
8	-2.776796000	1.170992000	-0.086163000
8	-2.809051000	-1.102295000	0.001201000
1	-2.898407000	-1.356431000	0.932370000
6	2.117486000	-0.021442000	0.004294000
9	3.465094000	-0.054309000	0.013761000

E = –518.642995 au   ZPVE = 0.10607700 au ν_i = 529.37i cm^{-1}
For deuterated acid (***TS*-5b**): ZPVE = 0.102992813 au ν_i = 389.97i cm^{-1}

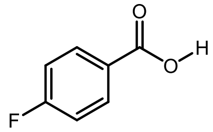

***Z*-1b**

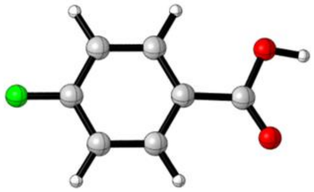

6	1.462691000	1.220017000	0.000201000
6	0.062385000	1.237406000	0.000160000
6	-0.661587000	0.029537000	0.000053000
6	0.021427000	-1.202529000	-0.000122000
6	1.423203000	-1.225996000	-0.000146000
1	2.049319000	2.142924000	0.000332000

1	-0.487841000	2.182419000	0.000256000
1	-0.543748000	-2.137567000	-0.000167000
1	1.980519000	-2.166895000	-0.000447000
6	-2.152128000	0.112435000	-0.000029000
8	-2.789251000	1.149748000	-0.000417000
8	-2.737994000	-1.113154000	0.000370000
1	-3.693658000	-0.925312000	0.000096000
6	2.122652000	-0.014298000	-0.000005000
9	3.471279000	-0.036417000	-0.000040000

E = –518.663283 au ZPVE = 0.107829 au
For deuterated acid (***Z*-5b**): ZPVE = 0.104364382 au

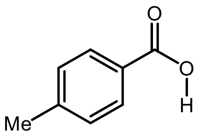

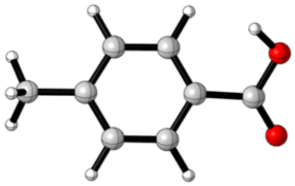

***E*-1c**

6	-1.402316000	-1.203344000	0.124404000
6	-0.002271000	-1.228029000	0.111580000
6	0.725097000	-0.028008000	-0.008660000
6	0.027147000	1.186979000	-0.156719000
6	-1.377110000	1.198952000	-0.155841000
1	-1.957338000	-2.143187000	0.227759000
1	0.549695000	-2.168809000	0.195734000
1	0.565057000	2.128634000	-0.319433000
1	-1.907833000	2.149078000	-0.285893000
6	2.226300000	-0.114763000	-0.026131000
8	2.832073000	-1.127773000	-0.299349000
8	2.895020000	1.031242000	0.287601000
1	2.248273000	1.659636000	0.642009000
6	-2.114372000	0.008400000	-0.008001000
6	-3.624742000	0.024353000	0.033583000
1	-3.988264000	-0.016097000	1.075442000
1	-4.027408000	0.939974000	-0.427974000
1	-4.045315000	-0.844217000	-0.498955000

E = –458.804279 au ZPVE = 0.143061 au
For deuterated acid (***E*-5c**): ZPVE = 0.139601992 au

***TS*-1c**

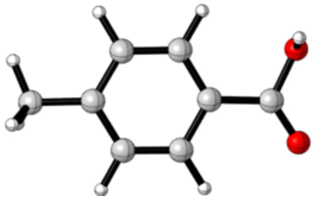

6	1.423438000	1.204259000	0.043647000
6	0.024881000	1.246287000	0.031941000
6	-0.715574000	0.046732000	-0.011607000

6	-0.038735000	-1.186566000	-0.054755000
6	1.365068000	-1.213894000	-0.045505000
1	1.992156000	2.141104000	0.080816000
1	-0.513671000	2.198282000	0.057728000
1	-0.612879000	-2.114978000	-0.109793000
1	1.885939000	-2.177651000	-0.082738000
6	-2.208631000	0.129932000	-0.020221000
8	-2.825090000	1.170529000	-0.079664000
8	-2.857164000	-1.102916000	-0.004304000
1	-2.954221000	-1.354985000	0.926486000
6	2.118055000	-0.025121000	0.005623000
6	3.629054000	-0.048876000	0.011317000
1	4.028366000	0.470798000	0.898901000
1	4.013175000	-1.081017000	0.017424000
1	4.033821000	0.461035000	-0.879728000

E = –458.794799 au   ZPVE = 0.141478 au ν_i = 535.23i cm^{-1}

For deuterated acid (***TS*-5c**): ZPVE = 0.138482629 au ν_i = 393.85i cm^{-1}

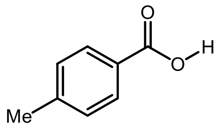

***Z*-1c**

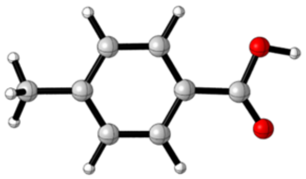

6	1.418209000	1.208433000	-0.010124000
6	0.017537000	1.236149000	-0.004076000
6	-0.707938000	0.029861000	0.000240000
6	-0.021966000	-1.199579000	-0.004186000
6	1.380871000	-1.211357000	-0.010071000
1	1.977235000	2.151522000	-0.017158000
1	-0.531983000	2.182094000	-0.005028000
1	-0.585412000	-2.136212000	-0.005505000
1	1.910735000	-2.171082000	-0.016995000
6	-2.198860000	0.111188000	0.003327000
8	-2.838965000	1.147000000	0.005290000
8	-2.785753000	-1.114808000	0.004442000
1	-3.740816000	-0.924187000	0.006867000
6	2.123603000	-0.013234000	-0.013769000
6	3.634534000	-0.034887000	0.016920000
1	4.006589000	0.011722000	1.055729000
1	4.029524000	-0.957138000	-0.438715000
1	4.055921000	0.826305000	-0.526617000

E = –458.814926 au   ZPVE = 0.143328 au

For deuterated acid (***Z*-5c**): ZPVE = 0.139863474 au

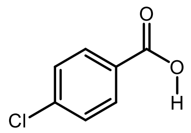

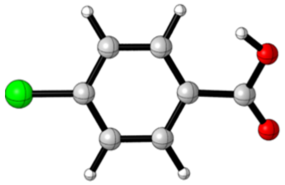

***E*-1d**

6	-1.009816000	-1.221651000	0.131024000
6	0.390226000	-1.229791000	0.114244000
6	1.111997000	-0.026159000	-0.006950000
6	0.411197000	1.188121000	-0.153217000
6	-0.991869000	1.206625000	-0.151408000
1	-1.575109000	-2.151834000	0.237830000
1	0.945716000	-2.168344000	0.198584000
1	0.945941000	2.131790000	-0.311925000
1	-1.540066000	2.144723000	-0.275344000
6	2.613972000	-0.111313000	-0.027470000
8	3.215213000	-1.125745000	-0.304436000
8	3.284022000	1.033232000	0.283274000
1	2.644903000	1.661111000	0.651975000
6	-1.693708000	0.000083000	-0.000440000
17	-3.435721000	0.016892000	0.013734000

E = –878.670972 au ZPVE = 0.105867 au
For deuterated acid (***E*-5d**): ZPVE = 0.102413514 au

***TS*-1d**

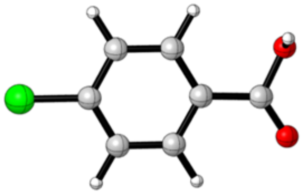

6	1.026834000	1.232241000	0.042195000
6	-0.372778000	1.252987000	0.032561000
6	-1.103406000	0.048058000	-0.011497000
6	-0.419730000	-1.182789000	-0.059694000
6	0.982246000	-1.212438000	-0.054064000
1	1.602908000	2.161185000	0.079681000
1	-0.917814000	2.201100000	0.060385000
1	-0.988691000	-2.114123000	-0.116900000
1	1.523584000	-2.161885000	-0.094966000
6	-2.597719000	0.121956000	-0.018160000
8	-3.217175000	1.159992000	-0.084441000
8	-3.236643000	-1.113878000	0.004790000
1	-3.323129000	-1.369150000	0.936000000
6	1.696252000	-0.003978000	-0.000374000
17	3.439029000	-0.036603000	0.011012000

E = –878.662144 au ZPVE = 0.104417 au $\nu_i = 530.21i\ cm^{-1}$
For deuterated acid (***TS*-5d**): ZPVE = 0.101334518 au $\nu_i = 390.50i\ cm^{-1}$

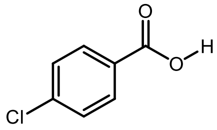

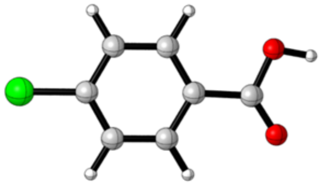

***Z*-1d**

6	1.023860000	1.229122000	0.000225000
6	-0.376626000	1.240162000	0.000183000
6	-1.096457000	0.030103000	0.000064000
6	-0.407731000	-1.198487000	-0.000140000
6	0.994245000	-1.217185000	-0.000145000
1	1.593531000	2.162701000	0.000376000
1	-0.929784000	2.183834000	0.000286000
1	-0.968313000	-2.136651000	-0.000169000
1	1.541609000	-2.164023000	-0.000481000
6	-2.588100000	0.107226000	-0.000034000
8	-3.227777000	1.142869000	-0.000463000
8	-3.168198000	-1.120454000	0.000399000
1	-4.125020000	-0.938111000	0.000121000
6	1.700745000	-0.002765000	-0.000017000
17	3.444480000	-0.024478000	-0.000025000

E = –878.682507 au ZPVE = 0.106176 au

For deuterated acid (***Z*-5d**): ZPVE = 0.102710869 au

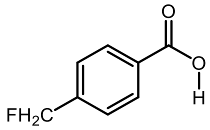

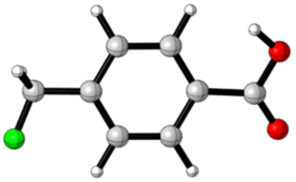

***E*-1e**

6	-1.089508000	-0.988162000	0.099111000
6	0.304317000	-1.138276000	0.091487000
6	1.141150000	-0.011805000	-0.012571000
6	0.563834000	1.267998000	-0.150095000
6	-0.830913000	1.413350000	-0.157504000
1	-1.739372000	-1.861847000	0.192894000
1	0.766222000	-2.126878000	0.170053000
1	1.190647000	2.155473000	-0.296431000
1	-1.269145000	2.411532000	-0.277756000
6	2.628367000	-0.239623000	-0.021874000
8	3.136608000	-1.300072000	-0.312049000
8	3.399649000	0.831841000	0.317834000
1	2.814648000	1.510389000	0.686651000
6	-1.666622000	0.288759000	-0.024158000
6	-3.167701000	0.467680000	-0.022811000
1	-3.477780000	1.103391000	0.825344000
1	-3.497173000	0.951044000	-0.959442000
9	-3.819517000	-0.750753000	0.089211000

E = –557.829474 au   ZPVE = 0.136321 au
For deuterated acid (***E*-5e**): ZPVE = 0.132863681 au

***TS*-1e**

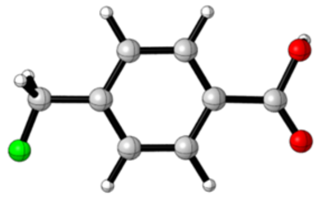

6	-1.105182000	-0.996169000	-0.021101000
6	0.286974000	-1.157798000	-0.017392000
6	1.131690000	-0.031228000	0.014058000
6	0.571352000	1.261784000	0.053832000
6	-0.821522000	1.416860000	0.053164000
1	-1.764186000	-1.867616000	-0.049400000
1	0.738692000	-2.154022000	-0.040140000
1	1.228694000	2.133600000	0.099363000
1	-1.252883000	2.424866000	0.087650000
6	2.612290000	-0.249177000	0.015373000
8	3.131304000	-1.340526000	0.086702000
8	3.368583000	0.919542000	-0.020848000
1	3.477924000	1.153447000	-0.955108000
6	-1.668500000	0.292536000	0.013970000
6	-3.167567000	0.485580000	0.019057000
1	-3.485984000	0.990702000	0.948167000
1	-3.477781000	1.107642000	-0.839106000
9	-3.833420000	-0.728341000	-0.062669000

E = –557.820329 au   ZPVE = 0.134854 au ν_i = 533.43i cm^{-1}
For deuterated acid (***TS*-5e**): ZPVE = 0.131771267 au ν_i = 392.70i cm^{-1}

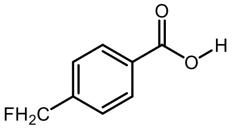

***Z*-1e**

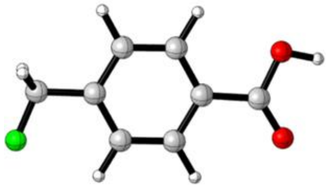

6	-1.102062000	-0.993435000	0.000022000
6	0.291972000	-1.144828000	0.000037000
6	1.125539000	-0.011830000	0.000040000
6	0.559127000	1.278784000	0.000018000
6	-0.834692000	1.422237000	-0.000031000
1	-1.754505000	-1.870211000	0.000053000
1	0.752936000	-2.136946000	0.000019000
1	1.208475000	2.157798000	0.000004000
1	-1.273281000	2.427696000	-0.000071000
6	2.603262000	-0.231536000	0.000002000
8	3.143163000	-1.322530000	-0.000093000
8	3.300034000	0.935096000	0.000064000

1	4.234025000	0.658904000	-0.000047000
6	-1.674131000	0.291007000	-0.000013000
6	-3.174394000	0.474338000	-0.000060000
1	-3.492797000	1.036978000	0.895401000
1	-3.492744000	1.036818000	-0.895640000
9	-3.832824000	-0.746666000	0.000048000

E = –557.840619 au   ZPVE = 0.13661 au

For deuterated acid (***Z*-5e**): ZPVE = 0.133145227 au

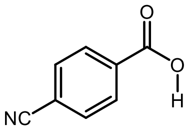

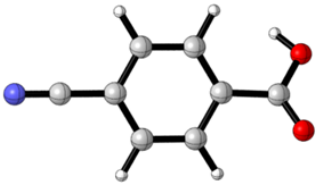

***E*-1f**

6	-1.127363000	1.223633000	0.134553000
6	0.271405000	1.230794000	0.118205000
6	0.989622000	0.024283000	-0.005213000
6	0.290336000	-1.191045000	-0.155854000
6	-1.111126000	-1.208028000	-0.155062000
6	-1.822997000	0.000709000	-0.000847000
1	-1.689737000	2.155850000	0.243868000
1	0.831356000	2.166394000	0.204014000
1	0.828098000	-2.132714000	-0.314451000
1	-1.657991000	-2.147186000	-0.281360000
6	2.493833000	0.109317000	-0.026847000
8	3.089797000	1.123972000	-0.313432000
8	3.165122000	-1.030823000	0.290337000
1	2.530811000	-1.659592000	0.665931000
6	-3.266542000	-0.013519000	0.010563000
7	-4.454985000	-0.026402000	0.021396000

E = –511.625818 au   ZPVE = 0.113246 au

***TS*-1f**

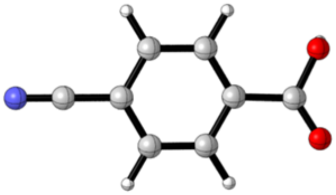

6	-1.144771000	1.233766000	0.044384000
6	0.253632000	1.253737000	0.035357000
6	0.980928000	0.045987000	-0.009713000
6	0.298927000	-1.186362000	-0.061164000
6	-1.101835000	-1.214705000	-0.056324000
6	-1.826442000	-0.004044000	-0.000739000
1	-1.717895000	2.165076000	0.083102000
1	0.803132000	2.199037000	0.064560000

1	0.870201000	-2.116040000	-0.119027000
1	-1.641446000	-2.165643000	-0.099619000
6	2.478470000	0.119160000	-0.017191000
8	3.093056000	1.159522000	-0.089927000
8	3.116569000	-1.113690000	0.009309000
1	3.200885000	-1.372215000	0.940024000
6	-3.269754000	-0.031089000	0.008177000
7	-4.458116000	-0.053654000	0.017026000

E = –511.61723 au ZPVE = 0.111788 au ν_i = 531.30*i* cm^{-1}

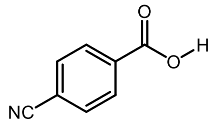

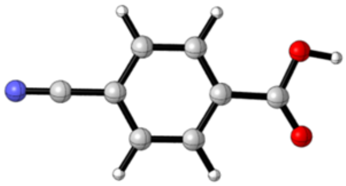

***Z*-1f**

6	1.142067000	1.230826000	-0.000350000
6	-0.257065000	1.241602000	-0.000266000
6	-0.973867000	0.028752000	-0.000078000
6	-0.287168000	-1.201868000	0.000191000
6	1.113414000	-1.219623000	0.000309000
6	1.831137000	-0.002969000	0.000001000
1	1.709135000	2.166596000	-0.000511000
1	-0.814498000	2.182562000	-0.000393000
1	-0.851026000	-2.137850000	0.000137000
1	1.658985000	-2.168114000	0.000499000
6	-2.468045000	0.105857000	0.000031000
8	-3.103658000	1.143622000	0.000685000
8	-3.045442000	-1.121180000	-0.000559000
1	-4.003467000	-0.943996000	-0.000291000
6	3.274696000	-0.020810000	0.000010000
7	4.463237000	-0.035620000	0.000068000

E = –511.637909 au ZPVE = 0.113568 au

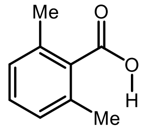

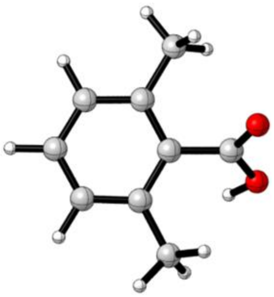

***E*-2a**

6	-1.988381000	1.144792000	-0.103848000
6	-0.584909000	1.230466000	-0.058931000
6	0.157551000	0.026795000	0.022265000
6	-0.480003000	-1.236110000	0.098008000

6	-1.887892000	-1.275890000	0.068709000
6	-2.639783000	-0.097366000	-0.040254000
1	-2.576498000	2.066358000	-0.179101000
1	-2.396205000	-2.244151000	0.141405000
1	-3.733560000	-0.145205000	-0.066227000
6	1.661008000	0.112729000	0.075420000
8	2.288487000	0.716239000	0.916913000
8	2.305552000	-0.551359000	-0.927722000
1	1.619732000	-0.886660000	-1.526246000
6	0.309274000	-2.524695000	0.226665000
1	0.675058000	-2.890679000	-0.748975000
1	1.186100000	-2.402883000	0.882665000
1	-0.327579000	-3.315686000	0.653099000
6	0.107405000	2.574956000	-0.086499000
1	0.624672000	2.766095000	0.866843000
1	0.872095000	2.619399000	-0.880336000
1	-0.621740000	3.380307000	-0.265866000

E = –497.985962 au ZPVE = 0.171019 au

For deuterated acid (***E*-6a**): ZPVE = 0.167541594 au

***TS*-2a**

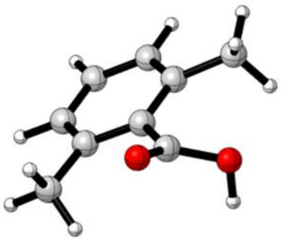

6	1.850743000	1.328456000	-0.058701000
6	0.445142000	1.253788000	-0.092140000
6	-0.160969000	-0.029098000	-0.025683000
6	0.623357000	-1.211228000	0.057941000
6	2.023751000	-1.082385000	0.118030000
6	2.639666000	0.175523000	0.058214000
1	2.330196000	2.311485000	-0.127382000
1	2.636728000	-1.986710000	0.202620000
1	3.731268000	0.256729000	0.094668000
6	-1.653998000	-0.174163000	-0.071406000
8	-2.239102000	-1.020798000	-0.708995000
8	-2.348559000	0.776550000	0.674930000
1	-2.451257000	0.403839000	1.564235000
6	0.009622000	-2.594996000	0.084734000
1	-0.807907000	-2.672525000	0.820050000
1	-0.422981000	-2.854405000	-0.893874000
1	0.777059000	-3.341334000	0.343894000
6	-0.349080000	2.536991000	-0.227921000
1	-1.172974000	2.437912000	-0.951763000
1	-0.801550000	2.833167000	0.731052000
1	0.313295000	3.348501000	-0.569392000

E = –497.972269 au ZPVE = 0.169752 au v_i = 509.05i cm^{-1}
For deuterated acid (***TS*-6a**): ZPVE = 0.166673498 au v_i = 418.54i cm^{-1}

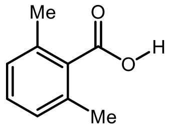

***Z*-2a**

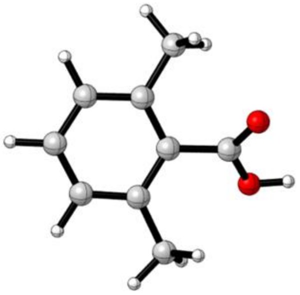

6	2.004996000	1.125115000	0.102225000
6	0.601432000	1.220552000	0.064223000
6	-0.152512000	0.020637000	-0.012977000
6	0.479404000	-1.247447000	-0.084517000
6	1.886865000	-1.290278000	-0.065762000
6	2.649460000	-0.118350000	0.034696000
1	2.597475000	2.043746000	0.178184000
1	2.388151000	-2.262375000	-0.133558000
1	3.743061000	-0.173695000	0.056003000
6	-1.645885000	0.136634000	-0.061637000
8	-2.268409000	0.932190000	-0.741372000
8	-2.261383000	-0.744291000	0.770854000
1	-3.213197000	-0.580317000	0.631940000
6	-0.292305000	-2.546258000	-0.195918000
1	-0.758203000	-2.818498000	0.764098000
1	-1.100760000	-2.480032000	-0.941572000
1	0.386210000	-3.359301000	-0.499325000
6	-0.053181000	2.584536000	0.110185000
1	-0.520549000	2.831580000	-0.855472000
1	-0.850430000	2.630554000	0.869910000
1	0.696930000	3.354300000	0.350830000

E = –497.993031 au ZPVE = 0.171518 au
For deuterated acid (***Z*-6a**): ZPVE = 0.168058167 au

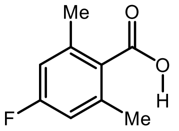

***E*-2b**

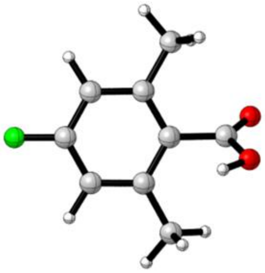

6	-1.591495000	1.210071000	0.089321000
6	-0.187415000	1.248887000	0.055253000
6	0.524539000	0.025755000	-0.021207000
6	-0.151666000	-1.216409000	-0.105822000

6	-1.559048000	-1.222757000	-0.087579000
6	-2.253684000	-0.017829000	0.017653000
1	-2.178489000	2.130887000	0.160058000
1	-2.119795000	-2.159296000	-0.166256000
6	2.029431000	0.073299000	-0.066324000
8	2.673960000	0.699545000	-0.877895000
8	2.655574000	-0.652483000	0.904747000
1	1.963559000	-0.988610000	1.495375000
6	0.591147000	-2.532526000	-0.233856000
1	1.492418000	-2.434410000	-0.858997000
1	0.908685000	-2.927687000	0.747351000
1	-0.062493000	-3.290548000	-0.693009000
6	0.534062000	2.577560000	0.090463000
1	1.299973000	2.598651000	0.883550000
1	1.055332000	2.760054000	-0.862091000
1	-0.176795000	3.397718000	0.274437000
9	-3.603765000	-0.037730000	0.041929000

E = –597.019494 au ZPVE = 0.163116 au

For deuterated acid (***E*-6b**): ZPVE = 0.159637594 au

***TS*-2b**

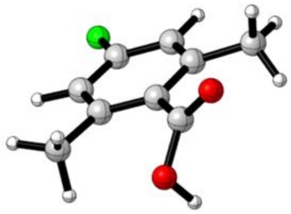

6	-1.602102000	1.197505000	-0.091356000
6	-0.197923000	1.246998000	-0.045640000
6	0.529416000	0.027340000	0.027829000
6	-0.144929000	-1.221798000	0.099259000
6	-1.551044000	-1.230548000	0.079466000
6	-2.256434000	-0.032271000	-0.026847000
1	-2.193579000	2.114874000	-0.167126000
1	-2.105691000	-2.171098000	0.150941000
6	2.026924000	0.099005000	0.058983000
8	2.657985000	0.943161000	0.655070000
8	2.669311000	-0.916061000	-0.647417000
1	2.781433000	-0.590391000	-1.553825000
6	0.572819000	-2.549975000	0.230329000
1	1.003608000	-2.868852000	-0.731100000
1	1.403553000	-2.499165000	0.950523000
1	-0.135100000	-3.321624000	0.571954000
6	0.476763000	2.601773000	-0.079443000
1	0.928808000	2.840002000	0.895506000
1	1.291306000	2.638989000	-0.820475000
1	-0.258112000	3.381678000	-0.333016000
9	-3.606171000	-0.063264000	-0.060009000

E = –597.006293 au ZPVE = 0.161883 au ν_i = 566.84*i* cm^{-1}

For deuterated acid (***TS*-6b**): ZPVE = 0.158808478 au   *v*i = 416.47*i* cm^{-1}

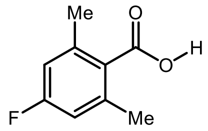

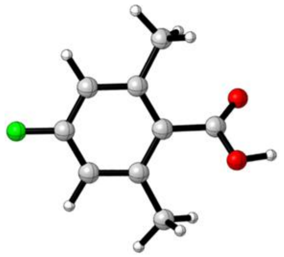

***Z*-2b**

6	-1.600706000	1.202773000	0.085634000
6	-0.195935000	1.243420000	0.056138000
6	0.521296000	0.019710000	-0.014831000
6	-0.157115000	-1.225156000	-0.087769000
6	-1.563664000	-1.226242000	-0.077222000
6	-2.262171000	-0.023083000	0.016596000
1	-2.186833000	2.124007000	0.155607000
1	-2.123225000	-2.164044000	-0.145537000
6	2.016667000	0.089314000	-0.052259000
8	2.667531000	0.904149000	-0.681060000
8	2.601816000	-0.858037000	0.727502000
1	3.558508000	-0.715913000	0.599150000
6	0.558561000	-2.556110000	-0.195559000
1	1.371875000	-2.523644000	-0.937266000
1	1.008991000	-2.844684000	0.766758000
1	-0.153228000	-3.339473000	-0.499820000
6	0.493323000	2.590068000	0.105748000
1	1.291802000	2.611910000	0.864490000
1	0.967493000	2.824999000	-0.859338000
1	-0.236957000	3.378008000	0.347437000
9	-3.612750000	-0.045358000	0.035347000

E = –597.027054 au   ZPVE = 0.163669 au
For deuterated acid (***Z*-6b**): ZPVE = 0.160206167 au

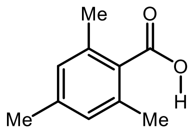

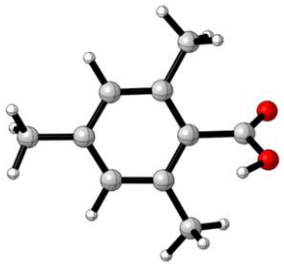

***E*-2c**

6	-1.554765000	1.196528000	0.095392000
6	-0.151139000	1.246880000	0.055138000
6	0.563105000	0.026110000	-0.026020000
6	-0.114918000	-1.214180000	-0.104245000
6	-1.523198000	-1.210071000	-0.081076000
6	-2.261470000	-0.019785000	0.028416000
1	-2.114243000	2.137168000	0.174741000

1	-2.055713000	-2.166641000	-0.154844000
6	2.067681000	0.072525000	-0.069577000
8	2.715940000	0.691838000	-0.883699000
8	2.690764000	-0.647237000	0.908952000
1	1.994046000	-0.983842000	1.493825000
6	0.627110000	-2.531331000	-0.231160000
1	1.521024000	-2.439135000	-0.868370000
1	0.957850000	-2.922345000	0.747667000
1	-0.031995000	-3.292690000	-0.677589000
6	0.570397000	2.575967000	0.091729000
1	1.332125000	2.601138000	0.889241000
1	1.096933000	2.759933000	-0.857865000
1	-0.142369000	3.396084000	0.270817000
6	-3.773104000	-0.033341000	0.026828000
1	-4.177542000	0.703010000	0.740788000
1	-4.168051000	0.221472000	-0.972408000
1	-4.163900000	-1.026779000	0.299420000

E = –537.17139 au ZPVE = 0.19858 au
For deuterated acid (***E*-6c**): ZPVE = 0.195101355 au

***TS*-2c**

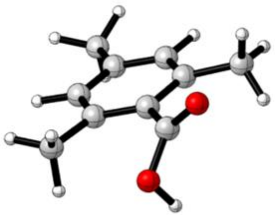

6	-1.562913000	1.190115000	-0.080409000
6	-0.158116000	1.246414000	-0.041086000
6	0.567322000	0.026849000	0.028405000
6	-0.112300000	-1.217698000	0.105459000
6	-1.519147000	-1.211234000	0.093580000
6	-2.265536000	-0.024954000	-0.010639000
1	-2.124649000	2.129839000	-0.151559000
1	-2.048065000	-2.169061000	0.175647000
6	2.065080000	0.092455000	0.053695000
8	2.704057000	0.926494000	0.655690000
8	2.700012000	-0.917427000	-0.669007000
1	2.811255000	-0.577889000	-1.570324000
6	0.603333000	-2.547352000	0.235472000
1	1.031168000	-2.870517000	-0.726117000
1	1.436468000	-2.499762000	0.953836000
1	-0.105534000	-3.317161000	0.580513000
6	0.521095000	2.599260000	-0.075564000
1	0.982285000	2.835630000	0.895810000
1	1.329470000	2.638857000	-0.823859000
1	-0.213887000	3.382274000	-0.320791000
6	-3.775034000	-0.057798000	-0.077088000
1	-4.212384000	0.864982000	0.337532000
1	-4.121623000	-0.151897000	-1.121616000

1	-4.179755000	-0.914161000	0.486519000

E = –537.157774 au ZPVE = 0.197333 au v_i = 568.81*i* cm^{-1}
For deuterated acid (***TS*-6c**): ZPVE = 0.194256813 au v_i = 417.91*i* cm^{-1}

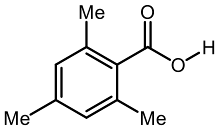

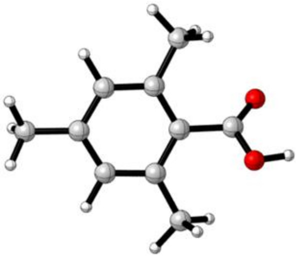

***Z*-2c**

6	-1.562782000	1.191986000	0.094878000
6	-0.157895000	1.241664000	0.059278000
6	0.558717000	0.019432000	-0.016611000
6	-0.123075000	-1.222252000	-0.086237000
6	-1.530263000	-1.210631000	-0.070617000
6	-2.270706000	-0.020580000	0.028740000
1	-2.119929000	2.133790000	0.174285000
1	-2.063195000	-2.167567000	-0.134846000
6	2.054127000	0.085654000	-0.057395000
8	2.706627000	0.886142000	-0.702935000
8	2.639032000	-0.847886000	0.740007000
1	3.595529000	-0.709054000	0.606817000
6	0.591907000	-2.553920000	-0.192893000
1	1.402141000	-2.526003000	-0.938878000
1	1.046867000	-2.842701000	0.767534000
1	-0.121846000	-3.337840000	-0.492469000
6	0.535504000	2.586472000	0.110040000
1	1.332047000	2.608700000	0.871502000
1	1.012907000	2.822524000	-0.853457000
1	-0.193515000	3.376632000	0.350037000
6	-3.782151000	-0.039855000	0.017750000
1	-4.194820000	0.797428000	0.603995000
1	-4.170025000	0.050800000	-1.012484000
1	-4.171726000	-0.980568000	0.439783000

E = –537.178459 au ZPVE = 0.199115 au
For deuterated acid (***Z*-6c**): ZPVE = 0.195652175 au

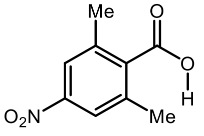

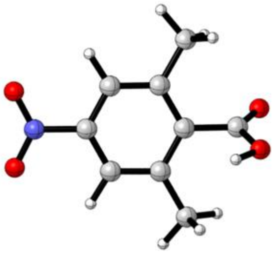

***E*-2d**

6	-0.973760000	1.230323000	0.062022000

6	0.430744000	1.254921000	0.036564000
6	1.131082000	0.023675000	-0.020356000
6	0.450030000	-1.217046000	-0.096939000
6	-0.956418000	-1.217157000	-0.087074000
1	-1.555611000	2.153338000	0.118350000
1	-1.524498000	-2.148065000	-0.155208000
6	2.637883000	0.055244000	-0.065901000
8	3.283016000	0.597395000	-0.934928000
8	3.254924000	-0.582727000	0.967929000
1	2.560786000	-0.866857000	1.583409000
6	1.172300000	2.571543000	0.060577000
1	1.923533000	2.592122000	0.867809000
1	1.713050000	2.724526000	-0.886563000
1	0.473102000	3.407054000	0.215436000
6	1.198895000	-2.530192000	-0.198968000
1	2.073552000	-2.445069000	-0.863453000
1	1.561369000	-2.875679000	0.784632000
1	0.537233000	-3.313209000	-0.600018000
6	-1.638547000	0.000252000	-0.000391000
7	-3.119000000	-0.012797000	0.017844000
8	-3.675558000	-1.111160000	-0.051893000
8	-3.692728000	1.075498000	0.103079000

E = –702.013457 au   ZPVE = 0.173673 au

***TS*-2d**

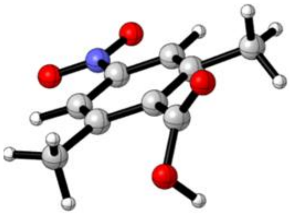

6	-0.978923000	1.225736000	-0.066014000
6	0.425860000	1.256289000	-0.030489000
6	1.136697000	0.026016000	0.030112000
6	0.453141000	-1.218199000	0.101919000
6	-0.952843000	-1.216094000	0.093388000
1	-1.562005000	2.147189000	-0.132032000
1	-1.517592000	-2.148819000	0.162115000
6	2.637991000	0.075434000	0.053560000
8	3.279943000	0.861678000	0.712865000
8	3.255271000	-0.891674000	-0.732262000
1	3.361280000	-0.507071000	-1.616383000
6	1.126178000	2.597073000	-0.057660000
1	1.595625000	2.810840000	0.914781000
1	1.930444000	2.625626000	-0.810517000
1	0.404516000	3.394866000	-0.290746000
6	1.171228000	-2.546379000	0.213592000
1	1.589322000	-2.853572000	-0.757147000
1	2.011044000	-2.498604000	0.923920000
1	0.468700000	-3.321244000	0.557711000
6	-1.641388000	-0.003448000	-0.002188000

7	-3.120859000	-0.020941000	-0.028064000
8	-3.675303000	-1.120113000	0.048820000
8	-3.697782000	1.064960000	-0.125994000

E = –702.000379 au ZPVE = 0.1724 au ν_i = 569.38i cm^{-1}

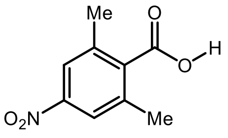

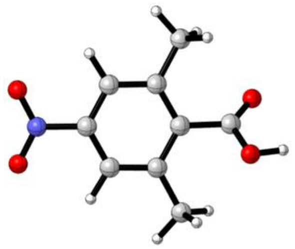

***Z*-2d**

6	-0.979967000	1.225157000	0.068373000
6	0.424939000	1.250909000	0.044652000
6	1.129321000	0.018798000	-0.015455000
6	0.445079000	-1.223223000	-0.086107000
6	-0.961124000	-1.217672000	-0.082167000
1	-1.560214000	2.148618000	0.130511000
1	-1.528399000	-2.149102000	-0.146211000
6	2.627831000	0.071619000	-0.052793000
8	3.282980000	0.827300000	-0.746449000
8	3.194288000	-0.815649000	0.803602000
1	4.155025000	-0.698116000	0.678066000
6	1.136461000	2.584892000	0.087754000
1	1.923808000	2.599282000	0.858555000
1	1.627419000	2.796159000	-0.874445000
1	0.418173000	3.389044000	0.309145000
6	1.163839000	-2.552212000	-0.179454000
1	1.979096000	-2.521607000	-0.919550000
1	1.611330000	-2.827284000	0.788150000
1	0.456100000	-3.340724000	-0.478366000
6	-1.645928000	-0.002227000	0.002045000
7	-3.125512000	-0.015126000	0.016633000
8	-3.682979000	-1.113026000	-0.057262000
8	-3.700098000	1.073046000	0.102187000

E = –702.021397 au ZPVE = 0.174228 au

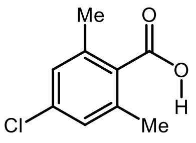

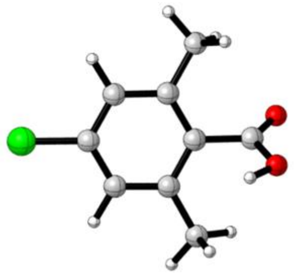

***E*-2e**

6	-1.213104000	1.221123000	0.078755000
6	0.191693000	1.251731000	0.049706000

6	0.897152000	0.025194000	-0.022666000
6	0.214664000	-1.212971000	-0.110026000
6	-1.192891000	-1.212244000	-0.097923000
1	-1.781974000	2.153507000	0.147698000
1	-1.744711000	-2.154053000	-0.178515000
6	2.402852000	0.062836000	-0.062118000
8	3.053240000	0.668847000	-0.884088000
8	3.019565000	-0.647857000	0.925309000
1	2.322971000	-0.970840000	1.518064000
6	0.921957000	2.575562000	0.087051000
1	1.679765000	2.593741000	0.888137000
1	1.453641000	2.750452000	-0.861232000
1	0.215406000	3.401592000	0.260914000
6	0.953442000	-2.531750000	-0.232763000
1	1.848519000	-2.439745000	-0.868025000
1	1.280740000	-2.917684000	0.748743000
1	0.294558000	-3.293014000	-0.678801000
6	-1.893018000	-0.003514000	0.005163000
17	-3.638109000	-0.020452000	0.030716000

E = –957.039175 au   ZPVE = 0.16144161 au
For deuterated acid (***E*-6e**): ZPVE = 0.157966183 au

***TS*-2e**

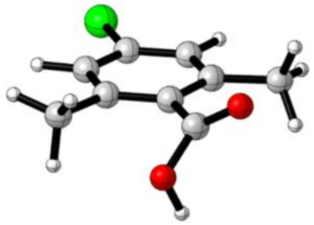

6	-1.189155000	1.213017000	-0.095859000
6	0.217469000	1.216145000	-0.107303000
6	0.902303000	-0.026230000	-0.030047000
6	0.185970000	-1.251886000	0.040189000
6	-1.219193000	-1.215365000	0.079017000
1	-1.738047000	2.156718000	-0.172044000
1	-1.789496000	-2.146323000	0.153324000
6	2.401425000	-0.082723000	-0.052433000
8	3.042946000	-0.910384000	-0.660079000
8	3.027791000	0.926611000	0.674591000
1	3.135800000	0.589022000	1.577219000
6	0.926501000	2.549287000	-0.234431000
1	1.349090000	2.870987000	0.729698000
1	1.761744000	2.503302000	-0.949994000
1	0.215723000	3.316069000	-0.580975000
6	0.874532000	-2.599603000	0.077256000
1	1.682037000	-2.629657000	0.826502000
1	0.145893000	-3.388061000	0.322067000
1	1.338024000	-2.830241000	-0.894164000
6	-1.895776000	0.008672000	0.009577000
17	-3.640654000	0.032969000	0.044498000

E = –957.025984 au ZPVE = 0.16019898 au ν_i = 567.41i cm^{-1}
For deuterated acid (***TS*-6e**): ZPVE = 0.157123076 au ν_i = 417.00i cm^{-1}

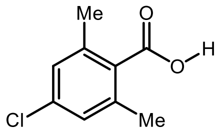

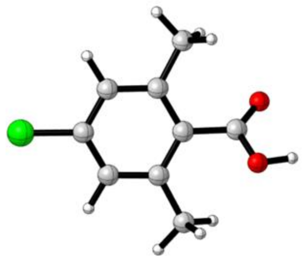

***Z*-2e**

6	-1.220200000	1.215707000	0.079279000
6	0.185212000	1.246794000	0.053510000
6	0.894452000	0.019010000	-0.015449000
6	0.208725000	-1.220915000	-0.092553000
6	-1.198217000	-1.213719000	-0.086950000
1	-1.787375000	2.148913000	0.148826000
1	-1.749889000	-2.156092000	-0.158597000
6	2.391126000	0.077758000	-0.049685000
8	3.047956000	0.875835000	-0.693051000
8	2.965095000	-0.858682000	0.750199000
1	3.923835000	-0.727205000	0.625064000
6	0.885382000	2.587699000	0.105320000
1	1.678629000	2.603667000	0.869811000
1	1.367374000	2.816518000	-0.857375000
1	0.160576000	3.382440000	0.340956000
6	0.918637000	-2.555075000	-0.198073000
1	1.732900000	-2.526054000	-0.939075000
1	1.366130000	-2.844384000	0.765376000
1	0.204228000	-3.335688000	-0.503080000
6	-1.900686000	-0.006150000	0.006317000
17	-3.646317000	-0.023882000	0.025918000

E = –957.046777 au ZPVE = 0.161985179 au
For deuterated acid (***Z*-6e**): ZPVE = 0.15852588 au

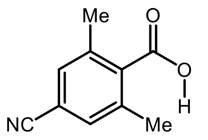

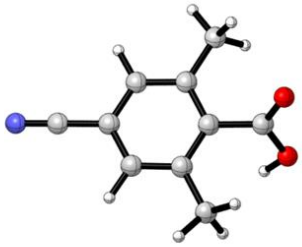

***E*-2f**

6	1.320776000	1.223735000	0.074582000
6	-0.082694000	1.253401000	0.047286000
6	-0.783016000	0.023261000	-0.020670000
6	-0.104084000	-1.217720000	-0.105856000
6	1.301494000	-1.215715000	-0.094281000
6	2.012829000	-0.003357000	0.003875000

1	1.887421000	2.158423000	0.140114000
1	1.852189000	-2.159256000	-0.170219000
6	-2.290133000	0.055780000	-0.065714000
8	-2.935094000	0.599691000	-0.933480000
8	-2.906458000	-0.583862000	0.967470000
1	-2.211734000	-0.869268000	1.581737000
6	-0.855691000	-2.528554000	-0.219674000
1	-1.229699000	-2.877284000	0.758357000
1	-1.723230000	-2.437897000	-0.892875000
1	-0.193528000	-3.312636000	-0.618162000
6	-0.824284000	2.570113000	0.080616000
1	-1.364072000	2.731312000	-0.865709000
1	-1.575852000	2.585242000	0.887690000
1	-0.125674000	3.404974000	0.242658000
6	3.455820000	-0.017568000	0.024927000
7	4.644355000	-0.030069000	0.044851000

E = –589.995049 au   ZPVE = 0.168803108 au

For deuterated acid (***E*-6f**): ZPVE = 0.165334359 au

***TS*-2f**

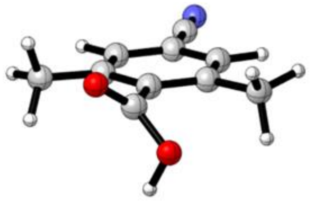

6	1.297031000	1.216250000	-0.098992000
6	-0.108050000	1.219342000	-0.110032000
6	-0.788539000	-0.025484000	-0.028733000
6	-0.076549000	-1.253883000	0.043767000
6	1.327142000	-1.216777000	0.082003000
6	2.015903000	0.009771000	0.009292000
1	1.843779000	2.161855000	-0.176210000
1	1.895671000	-2.149424000	0.158059000
6	-2.290151000	-0.077533000	-0.054095000
8	-2.929374000	-0.869656000	-0.708582000
8	-2.910111000	0.894471000	0.723548000
1	-3.017289000	0.516175000	1.610253000
6	-0.775064000	-2.595670000	0.081204000
1	-1.579414000	-2.619846000	0.834170000
1	-1.243397000	-2.819144000	-0.889554000
1	-0.052885000	-3.391018000	0.321768000
6	-0.829035000	2.545095000	-0.234628000
1	-1.665283000	2.489991000	-0.948735000
1	-1.252295000	2.859618000	0.731455000
1	-0.127666000	3.319576000	-0.582497000
6	3.458404000	0.030012000	0.038547000
7	4.646872000	0.046708000	0.064511000

E = –589.981985 au   ZPVE = 0.167554741 au ν_i = 568.63*i* cm^{-1}

For deuterated acid (***TS*-6f**): ZPVE = 0.164481833 au $\nu_i = 418.32i\ cm^{-1}$

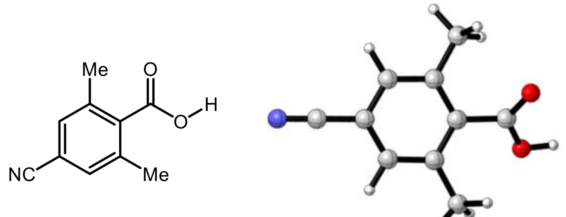

***Z*-2f**

6	1.328098000	1.217781000	0.081654000
6	-0.075827000	1.248976000	0.056178000
6	-0.780418000	0.018212000	-0.014617000
6	-0.098633000	-1.224035000	-0.094378000
6	1.306736000	-1.216679000	-0.088642000
6	2.020949000	-0.006551000	0.006633000
1	1.893184000	2.152866000	0.152528000
1	1.856442000	-2.160889000	-0.160626000
6	-2.279267000	0.072607000	-0.053491000
8	-2.932507000	0.829397000	-0.747331000
8	-2.847105000	-0.814927000	0.801678000
1	-3.807703000	-0.696762000	0.675174000
6	-0.820343000	-2.550774000	-0.199341000
1	-1.272307000	-2.832464000	0.764226000
1	-1.632272000	-2.513225000	-0.942918000
1	-0.114238000	-3.339639000	-0.501828000
6	-0.787134000	2.583002000	0.109022000
1	-1.279283000	2.801854000	-0.850843000
1	-1.573003000	2.592700000	0.881507000
1	-0.069057000	3.386162000	0.335453000
6	3.463800000	-0.021177000	0.022852000
7	4.652482000	-0.033505000	0.036761000

E = –590.002973 au ZPVE = 0.169363283 au
For deuterated acid (***Z*-6f**): ZPVE = 0.165902789 au

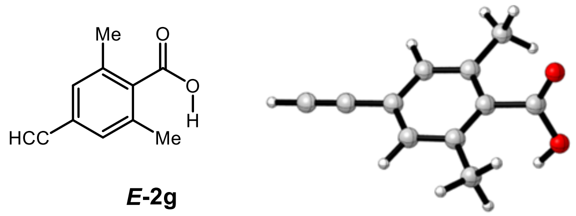

***E*-2g**

6	1.303095000	1.219783000	0.076685000
6	-0.099988000	1.252860000	0.047836000
6	-0.803939000	0.024721000	-0.023605000
6	-0.121778000	-1.214706000	-0.109823000
6	1.283780000	-1.210114000	-0.097939000
6	2.007060000	-0.002987000	0.004482000
1	1.867832000	2.155834000	0.145600000
1	1.832190000	-2.155156000	-0.177692000

6	-2.309771000	0.059406000	-0.061513000
8	-2.963174000	0.646888000	-0.894521000
8	-2.922403000	-0.631462000	0.942680000
1	-2.222252000	-0.944745000	1.536624000
6	-0.865754000	-2.530446000	-0.230322000
1	-1.211559000	-2.904755000	0.749214000
1	-1.750411000	-2.439450000	-0.880618000
1	-0.204465000	-3.299655000	-0.658873000
6	-0.835642000	2.573541000	0.084696000
1	-1.372429000	2.744261000	-0.861606000
1	-1.589708000	2.591553000	0.889508000
1	-0.131505000	3.402909000	0.252784000
6	3.446395000	-0.016806000	0.025046000
6	4.681460000	-0.028077000	0.044581000
1	5.757413000	-0.037255000	0.059047000

E = –573.90906 au   ZPVE = 0.179041641 au

***TS*-2g**

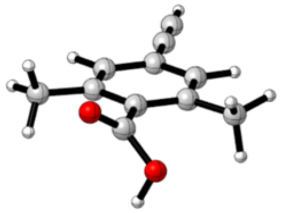

6	1.280649000	1.210687000	-0.097752000
6	-0.124229000	1.217932000	-0.108548000
6	-0.809015000	-0.025100000	-0.030147000
6	-0.094392000	-1.252504000	0.040832000
6	1.309013000	-1.213555000	0.079513000
6	2.010572000	0.008276000	0.009243000
1	1.826126000	2.157440000	-0.175076000
1	1.874856000	-2.148297000	0.155164000
6	-2.308396000	-0.079075000	-0.051861000
8	-2.952291000	-0.893306000	-0.674984000
8	-2.931453000	0.917237000	0.696200000
1	-3.040172000	0.561099000	1.591590000
6	-0.788700000	-2.597131000	0.079026000
1	-1.593569000	-2.625360000	0.831471000
1	-1.256431000	-2.825808000	-0.890879000
1	-0.062298000	-3.388627000	0.320864000
6	-0.837975000	2.548381000	-0.234587000
1	-1.673109000	2.500433000	-0.950533000
1	-1.261800000	2.868242000	0.729641000
1	-0.129837000	3.317781000	-0.580883000
6	3.449214000	0.027399000	0.037299000
6	4.684285000	0.043662000	0.062894000
1	5.760036000	0.057814000	0.083450000

E = –573.89568 au   ZPVE = 0.177773339 au ν_i = 568.63i cm^{-1}

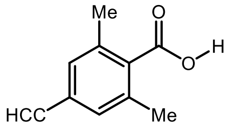

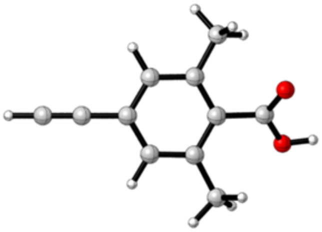

***Z*-2g**

6	1.310802000	1.213801000	0.079887000
6	-0.092847000	1.247722000	0.054292000
6	-0.800761000	0.018458000	-0.014997000
6	-0.115483000	-1.222388000	-0.093207000
6	1.289700000	-1.211680000	-0.088031000
6	2.015801000	-0.006102000	0.006258000
1	1.873872000	2.150577000	0.150445000
1	1.837638000	-2.157321000	-0.160665000
6	-2.297780000	0.074837000	-0.050145000
8	-2.955669000	0.856404000	-0.712546000
8	-2.869776000	-0.843302000	0.772064000
1	-3.828898000	-0.716235000	0.644925000
6	-0.830668000	-2.553484000	-0.198861000
1	-1.280738000	-2.841207000	0.763948000
1	-1.643751000	-2.522127000	-0.941514000
1	-0.118875000	-3.337057000	-0.502673000
6	-0.798866000	2.585341000	0.106891000
1	-1.287158000	2.810634000	-0.853542000
1	-1.587772000	2.599889000	0.876287000
1	-0.076281000	3.383762000	0.337132000
6	3.454842000	-0.020328000	0.022123000
6	4.690170000	-0.031811000	0.036039000
1	5.766058000	-0.041933000	0.048020000

E = –573.916441 au   ZPVE = 0.179556287 au

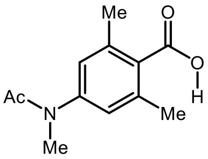

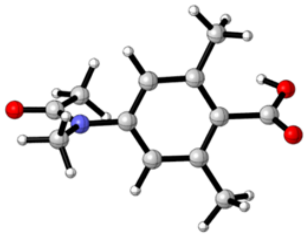

***E*-2h**

6	0.326515000	-1.326410000	0.118912000
6	-1.073721000	-1.264913000	0.220028000
6	-1.713717000	-0.029837000	-0.052171000
6	-0.979383000	1.111104000	-0.460349000
6	0.419116000	1.000177000	-0.574695000
6	1.078073000	-0.201437000	-0.266717000
1	0.852005000	-2.260627000	0.348220000
1	1.010938000	1.856997000	-0.917364000
6	-3.216094000	0.038158000	0.047573000
8	-3.977295000	-0.663758000	-0.579597000

8	-3.689777000	0.969026000	0.924458000
1	-2.919841000	1.350739000	1.374418000
6	-1.659064000	2.423328000	-0.800225000
1	-2.612948000	2.263990000	-1.327851000
1	-1.874974000	3.026114000	0.099510000
1	-1.006960000	3.028679000	-1.449202000
6	-1.870463000	-2.491423000	0.604618000
1	-2.544436000	-2.284782000	1.452934000
1	-2.503599000	-2.820655000	-0.234211000
1	-1.197576000	-3.314349000	0.891036000
7	2.494956000	-0.303223000	-0.399771000
6	3.007366000	-0.940242000	-1.610159000
1	4.103392000	-0.953094000	-1.537520000
1	2.625323000	-1.971473000	-1.694750000
1	2.703036000	-0.375607000	-2.508903000
6	3.381082000	0.267362000	0.505190000
8	4.596479000	0.244549000	0.332628000
6	2.746062000	0.906535000	1.731197000
1	2.285684000	1.873476000	1.470171000
1	1.960719000	0.269853000	2.167443000
1	3.544640000	1.080359000	2.465352000

E = –744.603988 au ZPVE = 0.253497594 au

***TS*-2h**

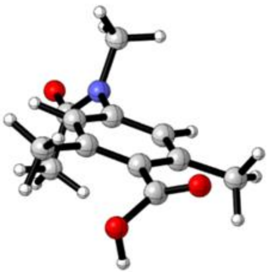

6	-0.421481000	0.998848000	-0.561015000
6	0.975592000	1.117005000	-0.447009000
6	1.719002000	-0.030702000	-0.062034000
6	1.074763000	-1.272481000	0.191941000
6	-0.326911000	-1.331540000	0.100521000
6	-1.081588000	-0.204755000	-0.265195000
1	-1.014864000	1.859902000	-0.889665000
1	-0.849443000	-2.268552000	0.324689000
6	3.214450000	0.028217000	0.056251000
8	3.962614000	-0.837814000	-0.337832000
8	3.699944000	1.194091000	0.642781000
1	3.738183000	1.027541000	1.597361000
6	1.838793000	-2.526291000	0.560439000
1	2.410277000	-2.905694000	-0.300660000
1	2.568074000	-2.344931000	1.366666000
1	1.139656000	-3.307676000	0.897164000
6	1.619749000	2.447126000	-0.780263000
1	1.921061000	2.985683000	0.131431000

1	2.526761000	2.323470000	-1.392085000
1	0.908137000	3.074261000	-1.340527000
7	-2.499314000	-0.304507000	-0.389779000
6	-3.019984000	-0.946681000	-1.593729000
1	-4.115666000	-0.955669000	-1.515337000
1	-2.718653000	-0.387925000	-2.497177000
1	-2.641666000	-1.979585000	-1.674769000
6	-3.379449000	0.275806000	0.514648000
8	-4.595801000	0.256962000	0.346732000
6	-2.737782000	0.918824000	1.735040000
1	-1.945019000	0.287356000	2.165215000
1	-2.285028000	1.888180000	1.469862000
1	-3.531596000	1.089021000	2.475269000

E = –744.590725 au   ZPVE = 0.252251139 au ν_i = 568.36*i* cm^{-1}

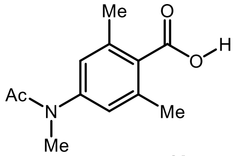

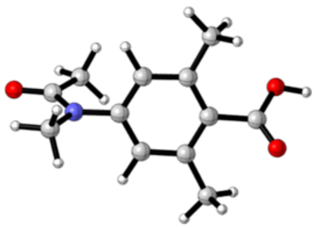

***Z*-2h**

6	0.332660000	-1.323982000	0.089476000
6	-1.068033000	-1.264999000	0.192545000
6	-1.710848000	-0.022671000	-0.051348000
6	-0.972846000	1.129282000	-0.427228000
6	0.424393000	1.014526000	-0.546074000
6	1.084966000	-0.193247000	-0.269683000
1	0.856672000	-2.262982000	0.301522000
1	1.016885000	1.880809000	-0.862273000
6	-3.205235000	0.024256000	0.057624000
8	-3.967627000	-0.812071000	-0.390483000
8	-3.638010000	1.112137000	0.746379000
1	-4.610890000	1.037111000	0.731320000
6	-1.623870000	2.462954000	-0.730044000
1	-2.511243000	2.348020000	-1.372703000
1	-1.954486000	2.963484000	0.193473000
1	-0.907922000	3.119808000	-1.248798000
6	-1.836922000	-2.514350000	0.564459000
1	-2.547211000	-2.327356000	1.386117000
1	-2.427863000	-2.881167000	-0.288849000
1	-1.140009000	-3.305172000	0.883246000
7	2.502283000	-0.291353000	-0.403824000
6	3.016116000	-0.899546000	-1.627843000
1	4.112160000	-0.912876000	-1.554858000
1	2.635226000	-1.929020000	-1.736733000
1	2.711677000	-0.314726000	-2.513651000
6	3.387197000	0.257576000	0.515255000
8	4.603171000	0.238004000	0.343651000
6	2.750584000	0.868490000	1.754428000

1	2.290641000	1.841196000	1.515224000
1	1.963184000	0.223369000	2.174073000
1	3.547948000	1.024675000	2.493883000

E = –744.611648 au   ZPVE = 0.25401702 au

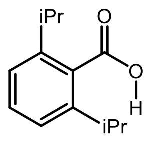

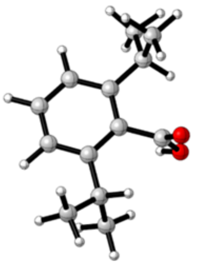

***E*-3a**

6	1.216369000	1.946674000	-0.005737000
6	1.237424000	0.539814000	-0.084664000
6	-0.000134000	-0.145240000	-0.004106000
6	-1.236409000	0.538068000	0.112602000
6	-1.211641000	1.945587000	0.168993000
6	0.004858000	2.643651000	0.121599000
1	2.154153000	2.508321000	-0.054419000
1	-2.148595000	2.504812000	0.250386000
6	-0.014379000	-1.652180000	-0.064352000
8	0.023889000	-2.313429000	-1.078055000
8	-0.076646000	-2.259348000	1.159881000
1	-0.109819000	-1.546099000	1.818334000
1	0.008395000	3.737732000	0.175055000
6	-2.545256000	-0.238120000	0.061399000
1	-2.367642000	-1.232970000	0.510199000
6	2.545246000	-0.231894000	-0.196176000
1	2.327080000	-1.174446000	-0.729201000
6	3.613070000	0.512148000	-1.008099000
1	4.479918000	-0.148024000	-1.179732000
1	3.222051000	0.832052000	-1.987993000
1	3.982244000	1.403968000	-0.472939000
6	3.076713000	-0.584948000	1.204355000
1	3.261624000	0.335130000	1.786813000
1	2.355512000	-1.209200000	1.756352000
1	4.024550000	-1.146545000	1.132652000
6	-2.951140000	-0.453023000	-1.407944000
1	-3.123759000	0.521920000	-1.897332000
1	-2.158612000	-0.984761000	-1.958851000
1	-3.881375000	-1.044325000	-1.472892000
6	-3.679253000	0.423039000	0.853995000
1	-4.555579000	-0.246110000	0.884549000
1	-3.375769000	0.643637000	1.891254000
1	-4.005129000	1.365668000	0.381961000

E = –654.716687 au   ZPVE = 0.285929546 au

***TS*-3a**

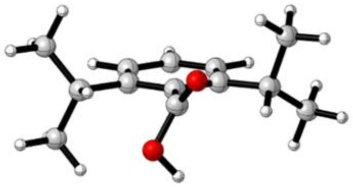

6	-1.190936000	1.918381000	0.011925000
6	-1.220008000	0.510699000	0.055748000
6	0.013750000	-0.184619000	-0.004865000
6	1.255756000	0.496877000	-0.093880000
6	1.233930000	1.905407000	-0.136820000
6	0.023499000	2.612396000	-0.088491000
1	-2.128901000	2.481332000	0.058880000
1	2.174230000	2.460236000	-0.210139000
6	0.012579000	-1.685783000	0.031076000
8	0.556755000	-2.358249000	0.876988000
8	-0.717281000	-2.268748000	-1.006811000
1	-0.092138000	-2.413623000	-1.734772000
1	0.027794000	3.707233000	-0.124173000
6	2.583517000	-0.249279000	-0.098495000
1	2.403028000	-1.280051000	-0.452307000
6	-2.552299000	-0.217468000	0.169379000
1	-2.355168000	-1.275458000	0.412106000
6	-3.420171000	0.350733000	1.302680000
1	-4.331964000	-0.259389000	1.423097000
1	-2.874176000	0.351392000	2.260840000
1	-3.741053000	1.384790000	1.089469000
6	-3.296768000	-0.179844000	-1.173864000
1	-3.510991000	0.863001000	-1.468454000
1	-2.687943000	-0.650336000	-1.961693000
1	-4.256939000	-0.720632000	-1.103532000
6	3.131258000	-0.345230000	1.335262000
1	3.309052000	0.666583000	1.741220000
1	2.412664000	-0.866859000	1.986110000
1	4.087466000	-0.897571000	1.351768000
6	3.619914000	0.378150000	-1.041480000
1	4.508508000	-0.272623000	-1.108086000
1	3.213015000	0.515696000	-2.057460000
1	3.963592000	1.359733000	-0.673338000

E = –654.70149 au   ZPVE = 0.284051378 au ν_i = 571.43i cm^{-1}

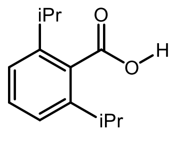

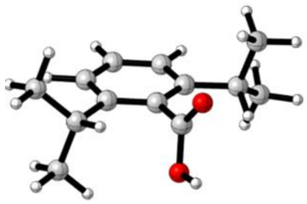

***Z*-3a**

6	-1.210128000	1.934710000	0.052789000

6	-1.227970000	0.525710000	0.078868000
6	0.010021000	-0.153574000	0.003412000
6	1.245197000	0.531653000	-0.088498000
6	1.217973000	1.939764000	-0.102467000
6	0.001235000	2.636554000	-0.034669000
1	-2.151381000	2.491617000	0.103498000
1	2.154422000	2.501811000	-0.171353000
6	0.024079000	-1.652947000	0.050483000
8	0.339133000	-2.330739000	1.010382000
8	-0.358552000	-2.191391000	-1.138231000
1	-0.326017000	-3.156891000	-0.993285000
1	-0.002014000	3.731858000	-0.050984000
6	2.558428000	-0.236219000	-0.117109000
1	2.352348000	-1.238998000	-0.535466000
6	-2.544152000	-0.234934000	0.141024000
1	-2.323630000	-1.275457000	0.440767000
6	-3.504157000	0.340503000	1.191797000
1	-4.398476000	-0.300133000	1.279766000
1	-3.022024000	0.402271000	2.181519000
1	-3.849971000	1.350596000	0.913024000
6	-3.197856000	-0.272001000	-1.249820000
1	-3.425049000	0.753084000	-1.592611000
1	-2.521593000	-0.742030000	-1.981721000
1	-4.142835000	-0.843190000	-1.224046000
6	3.087386000	-0.419887000	1.315539000
1	3.285292000	0.565596000	1.773540000
1	2.349801000	-0.954143000	1.934839000
1	4.029909000	-0.995563000	1.313165000
6	3.616371000	0.418036000	-1.015289000
1	4.495616000	-0.242707000	-1.103171000
1	3.223344000	0.610344000	-2.027522000
1	3.969053000	1.374769000	-0.593530000

E = –654.722497 au   ZPVE = 0.285775139 au

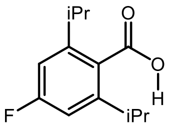

E-**3b**

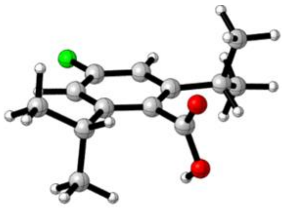

6	1.219535000	1.679565000	-0.053273000
6	1.236514000	0.273031000	-0.104579000
6	0.001850000	-0.415307000	-0.006188000
6	-1.233581000	0.271028000	0.098478000
6	-1.217793000	1.678250000	0.128122000
6	0.002134000	2.352718000	0.063290000
1	2.138935000	2.267151000	-0.114850000
1	-2.138853000	2.262451000	0.197862000
6	-0.010433000	-1.922745000	-0.044142000
8	0.063579000	-2.597416000	-1.046947000

8	-0.115645000	-2.513734000	1.184806000
1	-0.162465000	-1.792784000	1.833993000
6	-2.546093000	-0.499064000	0.057111000
1	-2.374524000	-1.485168000	0.526102000
6	2.548006000	-0.493306000	-0.206355000
1	2.338334000	-1.425572000	-0.760016000
6	3.630005000	0.267156000	-0.982326000
1	4.501945000	-0.388080000	-1.145515000
1	3.260485000	0.602397000	-1.965387000
1	3.985130000	1.150427000	-0.423773000
6	3.052170000	-0.871246000	1.198097000
1	3.228063000	0.038580000	1.799105000
1	2.321273000	-1.504201000	1.726662000
1	4.000539000	-1.432426000	1.132888000
6	-2.944078000	-0.740847000	-1.410405000
1	-3.111924000	0.224585000	-1.919750000
1	-2.150596000	-1.285547000	-1.947184000
1	-3.875440000	-1.330853000	-1.467903000
6	-3.679821000	0.184951000	0.829859000
1	-4.561199000	-0.476980000	0.866187000
1	-3.382060000	0.422002000	1.865072000
1	-3.995547000	1.121067000	0.338230000
9	0.004881000	3.704117000	0.105476000

E = –753.750731 au   ZPVE = 0.277991745 au

***TS*-3b**

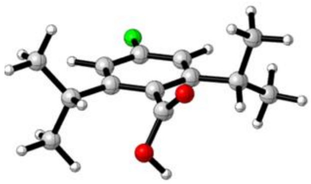

6	-1.189005000	1.652212000	0.044145000
6	-1.218990000	0.245558000	0.067249000
6	0.010047000	-0.458586000	-0.005485000
6	1.254277000	0.221658000	-0.084088000
6	1.245131000	1.629139000	-0.110046000
6	0.033279000	2.316770000	-0.049772000
1	-2.106528000	2.244246000	0.102678000
1	2.171067000	2.205830000	-0.177612000
6	0.004405000	-1.959298000	0.006521000
8	0.589891000	-2.645962000	0.812854000
8	-0.776872000	-2.525833000	-1.001956000
1	-0.190202000	-2.657146000	-1.763698000
6	2.584925000	-0.518766000	-0.097443000
1	2.408203000	-1.544670000	-0.464651000
6	-2.560715000	-0.465569000	0.179307000
1	-2.376008000	-1.526920000	0.413661000
6	-3.417749000	0.107663000	1.318175000
1	-4.336182000	-0.492458000	1.437043000
1	-2.869825000	0.096334000	2.275111000
1	-3.727756000	1.146415000	1.111579000

6	-3.307994000	-0.407371000	-1.161572000
1	-3.512449000	0.640182000	-1.446221000
1	-2.707505000	-0.877698000	-1.955687000
1	-4.273448000	-0.938466000	-1.092130000
6	3.130013000	-0.630487000	1.336308000
1	4.087798000	-1.179938000	1.345854000
1	3.304747000	0.376262000	1.755868000
1	2.412862000	-1.163860000	1.978957000
6	3.619266000	0.127694000	-1.029460000
1	4.511460000	-0.517250000	-1.101776000
1	3.214755000	0.275879000	-2.044845000
1	3.957072000	1.106152000	-0.647539000
9	0.045161000	3.668641000	-0.077424000

E = –753.735794 au   ZPVE = 0.276140159 au ν_i = 568.44i cm^{-1}

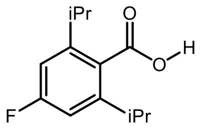

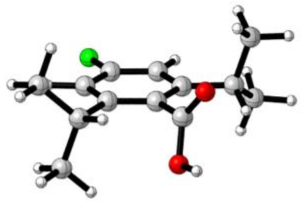

***Z*-3b**

6	1.209115000	1.661065000	-0.065069000
6	1.224672000	0.253231000	-0.080520000
6	-0.010144000	-0.432649000	-0.000450000
6	-1.245986000	0.253800000	0.085294000
6	-1.227741000	1.660693000	0.093615000
6	-0.008403000	2.335998000	0.020807000
1	2.131732000	2.245150000	-0.122170000
1	-2.148539000	2.245883000	0.160056000
6	-0.023763000	-1.931508000	-0.035578000
8	-0.427912000	-2.616418000	-0.956379000
8	0.468395000	-2.461802000	1.115678000
1	0.429654000	-3.428163000	0.978692000
6	-2.566259000	-0.501480000	0.118573000
1	-2.367676000	-1.509944000	0.524611000
6	2.550552000	-0.489802000	-0.149941000
1	2.340744000	-1.537170000	-0.429966000
6	3.485377000	0.086303000	-1.222897000
1	4.388614000	-0.541080000	-1.313847000
1	2.988863000	0.125533000	-2.206625000
1	3.818841000	1.105833000	-0.963947000
6	3.224845000	-0.496019000	1.231347000
1	3.445013000	0.536831000	1.554816000
1	2.566497000	-0.963753000	1.980566000
1	4.176400000	-1.055513000	1.198731000
6	-3.108124000	-0.666523000	-1.311434000
1	-3.299500000	0.324399000	-1.760363000
1	-2.381792000	-1.205396000	-1.939572000
1	-4.056792000	-1.231675000	-1.304634000
6	-3.608631000	0.158486000	1.030694000
1	-4.495327000	-0.492026000	1.118527000

1	-3.206365000	0.336166000	2.041947000
1	-3.952853000	1.123418000	0.620625000
9	-0.008270000	3.688634000	0.033501000

E = –753.756748 au   ZPVE = 0.277848478 au

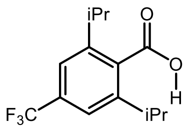

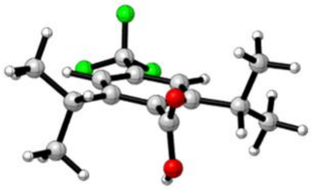

***E*-3c**

6	0.932104000	1.212720000	0.061350000
6	-0.473848000	1.238358000	0.106781000
6	-1.164221000	0.004677000	0.008060000
6	-0.484132000	-1.234970000	-0.091624000
6	0.922329000	-1.220783000	-0.116340000
6	1.620122000	-0.005398000	-0.049591000
1	1.502344000	2.143323000	0.115924000
1	1.483186000	-2.155350000	-0.193984000
6	-2.672949000	-0.001761000	0.036432000
8	-3.348953000	0.046390000	1.039509000
8	-3.257303000	-0.069121000	-1.196859000
1	-2.536047000	-0.108162000	-1.846294000
6	-1.263673000	-2.541654000	-0.064642000
1	-2.245060000	-2.360617000	-0.540308000
6	-1.236863000	2.551939000	0.198447000
1	-2.186367000	2.343361000	0.722632000
6	-0.492073000	3.620700000	1.008104000
1	-1.146879000	4.494481000	1.162620000
1	-0.187410000	3.237728000	1.995952000
1	0.408819000	3.976734000	0.479401000
6	-1.568878000	3.070927000	-1.212101000
1	-0.640588000	3.244786000	-1.784664000
1	-2.192080000	2.349811000	-1.765229000
1	-2.124334000	4.023012000	-1.154744000
6	-1.518035000	-2.945407000	1.399306000
1	-0.557119000	-3.122604000	1.913898000
1	-2.061386000	-2.151670000	1.936960000
1	-2.114300000	-3.873166000	1.447107000
6	-0.580310000	-3.675056000	-0.838725000
1	-1.248515000	-4.551042000	-0.886379000
1	-0.332140000	-3.372614000	-1.869959000
1	0.349044000	-4.000738000	-0.341093000
6	3.124064000	-0.010367000	-0.033353000
9	3.635114000	1.107913000	-0.589504000
9	3.603134000	-0.077881000	1.227056000
9	3.630314000	-1.065473000	-0.704848000

E = –991.03079 au   ZPVE = 0.290931219 au

***TS*-3c**

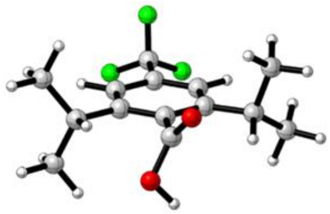

6	-0.877226000	-1.222491000	0.044777000
6	0.529102000	-1.215402000	0.070769000
6	1.194554000	0.034064000	-0.000451000
6	0.482914000	1.259127000	-0.084735000
6	-0.922992000	1.207905000	-0.111249000
6	-1.595058000	-0.021540000	-0.048552000
1	-1.422774000	-2.168953000	0.092005000
1	-1.505478000	2.129199000	-0.189299000
6	2.696898000	0.067363000	0.018719000
8	3.361812000	0.611050000	0.870244000
8	3.281907000	-0.632474000	-1.035221000
1	3.400429000	0.001803000	-1.760296000
6	1.196532000	2.603555000	-0.103839000
1	2.224512000	2.445715000	-0.476354000
6	1.287283000	-2.531292000	0.166431000
1	2.342359000	-2.311138000	0.402010000
6	0.745713000	-3.421095000	1.295579000
1	1.376684000	-4.320006000	1.402061000
1	0.740207000	-2.885102000	2.259273000
1	-0.282249000	-3.762958000	1.085810000
6	1.252178000	-3.261670000	-1.184814000
1	0.211510000	-3.497208000	-1.470399000
1	1.700849000	-2.635175000	-1.971438000
1	1.815392000	-4.209468000	-1.128482000
6	1.303579000	3.151022000	1.329590000
1	0.294897000	3.306732000	1.751784000
1	1.851711000	2.444510000	1.971803000
1	1.834685000	4.118957000	1.336615000
6	0.525294000	3.623173000	-1.034431000
1	1.151897000	4.527853000	-1.110977000
1	0.380069000	3.214311000	-2.048467000
1	-0.457717000	3.941258000	-0.647733000
6	-3.098104000	-0.047876000	-0.021968000
9	-3.590575000	-1.182786000	-0.560827000
9	-3.570354000	0.025393000	1.241221000
9	-3.631375000	0.988727000	-0.701846000

E = –991.015935 au ZPVE = 0.289031108 au ν_i = 571.47*i* cm^{-1}

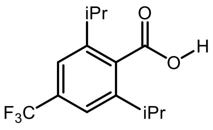

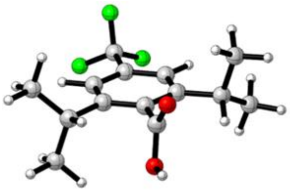

***Z*-3c**

6	0.920412000	1.213140000	0.052706000
6	-0.486347000	1.229793000	0.083415000
6	-1.168032000	-0.006719000	0.008383000
6	-0.484771000	-1.242276000	-0.090420000
6	0.921144000	-1.220807000	-0.110372000
6	1.614389000	-0.002296000	-0.039149000
1	1.484127000	2.148854000	0.095403000
1	1.486495000	-2.152397000	-0.193105000
6	-2.668252000	-0.018168000	0.062156000
8	-3.337493000	-0.290285000	1.040312000
8	-3.208378000	0.312477000	-1.139517000
1	-4.174306000	0.284427000	-0.995324000
6	-1.254386000	-2.553398000	-0.122147000
1	-2.251826000	-2.344590000	-0.551761000
6	-1.243970000	2.547002000	0.138419000
1	-2.277573000	2.330536000	0.464135000
6	-0.644704000	3.525448000	1.158005000
1	-1.284375000	4.420333000	1.243266000
1	-0.559937000	3.062608000	2.155123000
1	0.357867000	3.867265000	0.848987000
6	-1.306863000	3.172238000	-1.264947000
1	-0.288331000	3.392999000	-1.630452000
1	-1.790545000	2.482828000	-1.975327000
1	-1.877581000	4.117308000	-1.245434000
6	-1.450987000	-3.072887000	1.312652000
1	-0.469977000	-3.274315000	1.778344000
1	-1.985823000	-2.330565000	1.925835000
1	-2.031390000	-4.012141000	1.309289000
6	-0.591694000	-3.615784000	-1.008271000
1	-1.252285000	-4.494734000	-1.096694000
1	-0.389965000	-3.229310000	-2.021102000
1	0.360547000	-3.966531000	-0.575041000
6	3.117546000	-0.005046000	-0.008213000
9	3.634410000	1.128314000	-0.527541000
9	3.584785000	-0.107529000	1.255091000
9	3.633131000	-1.040956000	-0.702637000

E = –991.037109 au   ZPVE = 0.29080102 au

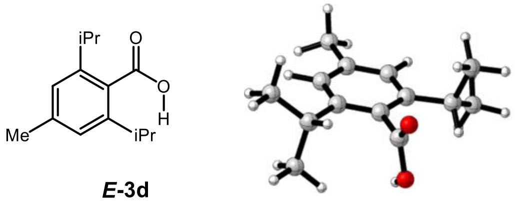

***E*-3d**

6	1.211156000	1.649191000	-0.046487000
6	1.234039000	0.242552000	-0.103401000
6	-0.000962000	-0.444162000	-0.011906000
6	-1.232173000	0.247501000	0.096518000
6	-1.201460000	1.654778000	0.128412000
6	0.008373000	2.370387000	0.070781000
1	2.153189000	2.205766000	-0.100732000
1	-2.140349000	2.213900000	0.205067000
6	-0.016846000	-1.951153000	-0.043811000
8	0.035181000	-2.632297000	-1.043766000
8	-0.097725000	-2.535234000	1.190995000
1	-0.136461000	-1.808729000	1.834509000
6	-2.545954000	-0.521694000	0.060364000
1	-2.374398000	-1.508657000	0.528466000
6	2.544043000	-0.527979000	-0.198922000
1	2.330254000	-1.474977000	-0.725886000
6	3.618383000	0.209430000	-1.008149000
1	4.486397000	-0.452205000	-1.168069000
1	3.235044000	0.522189000	-1.993391000
1	3.983825000	1.105213000	-0.476923000
6	3.065010000	-0.869851000	1.208367000
1	3.245188000	0.055235000	1.784462000
1	2.339533000	-1.489405000	1.760075000
1	4.013526000	-1.431868000	1.148807000
6	-2.952401000	-0.763625000	-1.404646000
1	-3.119669000	0.202425000	-1.913258000
1	-2.162118000	-1.309857000	-1.944623000
1	-3.885540000	-1.351565000	-1.458944000
6	-3.677268000	0.160594000	0.838747000
1	-4.556679000	-0.503794000	0.882875000
1	-3.372865000	0.401374000	1.871253000
1	-3.998879000	1.094786000	0.347282000
6	0.014547000	3.882565000	0.081255000
1	0.924306000	4.273986000	0.565280000
1	-0.014746000	4.283985000	-0.947277000
1	-0.860124000	4.281243000	0.620472000

E = –693.902201 au   ZPVE = 0.313454996 au

***TS*-3d**

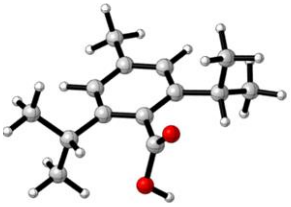

6	-1.177743000	1.627781000	0.035998000
6	-1.217657000	0.221226000	0.065883000
6	0.009470000	-0.484421000	-0.000111000
6	1.251013000	0.197608000	-0.083074000
6	1.231389000	1.605489000	-0.112075000
6	0.032166000	2.337590000	-0.058671000
1	-2.116427000	2.191981000	0.085281000
1	2.176915000	2.153973000	-0.184812000
6	0.003830000	-1.984761000	0.015914000
8	0.571203000	-2.670520000	0.835903000
8	-0.756094000	-2.553865000	-1.008750000
1	-0.150843000	-2.689613000	-1.754969000
6	2.580198000	-0.546379000	-0.099107000
1	2.400579000	-1.573873000	-0.461977000
6	-2.558453000	-0.493073000	0.172869000
1	-2.373186000	-1.555240000	0.405322000
6	-3.420376000	0.073638000	1.311568000
1	-4.337997000	-0.528722000	1.427416000
1	-2.873738000	0.060748000	2.269310000
1	-3.731662000	1.112587000	1.107801000
6	-3.303400000	-0.434845000	-1.169281000
1	-3.506525000	0.613024000	-1.453994000
1	-2.700022000	-0.904288000	-1.961905000
1	-4.269386000	-0.965960000	-1.103795000
6	3.132301000	-0.655628000	1.332043000
1	3.309726000	0.352403000	1.747659000
1	2.416135000	-1.184986000	1.979321000
1	4.089373000	-1.206811000	1.340750000
6	3.613726000	0.091290000	-1.038499000
1	4.501577000	-0.559391000	-1.115881000
1	3.203157000	0.240887000	-2.051328000
1	3.959908000	1.068332000	-0.660519000
6	0.045828000	3.849488000	-0.054266000
1	-0.863760000	4.257364000	-0.524863000
1	0.091513000	4.240615000	0.977706000
1	0.920027000	4.242021000	-0.598889000

E = –693.887042 au   ZPVE = 0.311595108 au v_i = 569.83*i* cm^{-1}

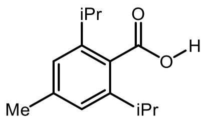

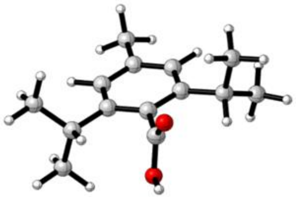

***Z*-3d**

6	1.200180000	1.636443000	-0.055649000

6	1.223478000	0.228361000	-0.079347000
6	-0.010358000	-0.457279000	-0.005147000
6	-1.242770000	0.232644000	0.085322000
6	-1.212152000	1.639463000	0.097722000
6	-0.003559000	2.357697000	0.031128000
1	2.144369000	2.191253000	-0.103572000
1	-2.151865000	2.198170000	0.170285000
6	-0.024905000	-1.955809000	-0.043945000
8	-0.396073000	-2.639286000	-0.979767000
8	0.426245000	-2.489868000	1.122916000
1	0.388803000	-3.455676000	0.981716000
6	-2.561597000	-0.526080000	0.119885000
1	-2.361167000	-1.533027000	0.530202000
6	2.546906000	-0.520198000	-0.143321000
1	2.334998000	-1.567216000	-0.424998000
6	3.489960000	0.050033000	-1.212458000
1	4.390660000	-0.581791000	-1.300367000
1	2.996873000	0.091650000	-2.197882000
1	3.827097000	1.068164000	-0.952755000
6	3.215782000	-0.531112000	1.240541000
1	3.436445000	0.500834000	1.566798000
1	2.551794000	-0.997641000	1.985750000
1	4.166224000	-1.093225000	1.212933000
6	-3.102933000	-0.698464000	-1.309428000
1	-3.295707000	0.290811000	-1.761619000
1	-2.374192000	-1.236666000	-1.935592000
1	-4.050370000	-1.266134000	-1.302904000
6	-3.608426000	0.131043000	1.029246000
1	-4.490886000	-0.524870000	1.121781000
1	-3.205938000	0.317152000	2.038959000
1	-3.959257000	1.091565000	0.614463000
6	-0.004834000	3.869756000	0.012387000
1	0.938947000	4.274166000	0.413189000
1	-0.121917000	4.251864000	-1.017329000
1	-0.834914000	4.274875000	0.614139000

E = –693.907943 au   ZPVE = 0.31330145 au

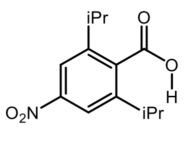

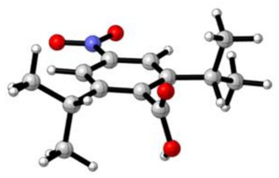

***E*-3e**

6	-1.215075000	1.210871000	-0.079899000
6	0.191231000	1.240631000	-0.110032000
6	0.890550000	0.011236000	-0.003937000
6	0.218252000	-1.234810000	0.082369000
6	-1.188483000	-1.238142000	0.091645000
6	-1.871473000	-0.019972000	0.022022000
1	-1.812176000	2.121703000	-0.146922000
1	-1.763494000	-2.163640000	0.151655000

6	2.399510000	0.015270000	-0.024873000
8	3.077715000	0.083456000	-1.025449000
8	2.980489000	-0.067786000	1.208482000
1	2.258704000	-0.118108000	1.856605000
6	1.005354000	-2.536505000	0.052765000
1	1.983153000	-2.352177000	0.534103000
6	0.944777000	2.559716000	-0.196819000
1	1.891641000	2.360765000	-0.729217000
6	0.185636000	3.630283000	-0.989896000
1	0.832184000	4.511203000	-1.137833000
1	-0.121128000	3.256344000	-1.980509000
1	-0.715465000	3.971284000	-0.451951000
6	1.282559000	3.066186000	1.217135000
1	0.356678000	3.230276000	1.796329000
1	1.913186000	2.343859000	1.760085000
1	1.832447000	4.021637000	1.164597000
6	1.268042000	-2.928301000	-1.413112000
1	0.310390000	-3.108456000	-1.932628000
1	1.809196000	-2.128084000	-1.943384000
1	1.870009000	-3.852066000	-1.463710000
6	0.322065000	-3.676888000	0.816247000
1	0.992221000	-4.551556000	0.858981000
1	0.070695000	-3.382815000	1.849077000
1	-0.605476000	-3.999744000	0.313678000
7	-3.352458000	-0.034500000	0.047301000
8	-3.906314000	-1.132406000	0.143597000
8	-3.931043000	1.052190000	-0.028099000

E = –858.745566 au   ZPVE = 0.288666406 au

***TS*-3e**

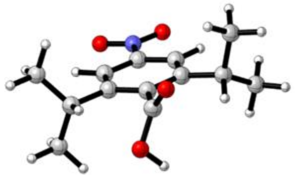

6	-0.967456000	-1.379046000	0.066605000
6	0.423965000	-1.172564000	0.075204000
6	0.911427000	0.157392000	-0.004018000
6	0.036768000	1.274350000	-0.075111000
6	-1.350195000	1.037399000	-0.084267000
6	-1.822219000	-0.276624000	-0.017811000
1	-1.399994000	-2.379733000	0.126575000
1	-2.072454000	1.853226000	-0.143975000
6	2.395162000	0.396076000	-0.001130000
8	2.986654000	1.032630000	0.840371000
8	3.060067000	-0.226130000	-1.055428000
1	3.081775000	0.410052000	-1.788244000
6	0.556252000	2.704684000	-0.094098000
1	1.592650000	2.692575000	-0.475692000
6	1.354714000	-2.372771000	0.165916000

1	2.372501000	-2.010886000	0.389156000
6	0.947895000	-3.323122000	1.302129000
1	1.694653000	-4.129043000	1.402845000
1	0.881308000	-2.790127000	2.265198000
1	-0.027365000	-3.799237000	1.103307000
6	1.402244000	-3.104830000	-1.184084000
1	0.399714000	-3.480343000	-1.455722000
1	1.752727000	-2.426260000	-1.977362000
1	2.089438000	-3.967112000	-1.132556000
6	0.597688000	3.254148000	1.342055000
1	-0.419423000	3.266691000	1.772313000
1	1.244462000	2.628244000	1.976050000
1	0.989141000	4.286341000	1.349813000
6	-0.261462000	3.622504000	-1.013125000
1	0.228356000	4.608099000	-1.086977000
1	-0.353233000	3.203429000	-2.029213000
1	-1.277211000	3.792533000	-0.618008000
7	-3.284480000	-0.508460000	-0.031882000
8	-4.015057000	0.482867000	-0.105002000
8	-3.673461000	-1.677468000	0.029570000

E = –858.730878 au ZPVE = 0.286768908 au ν_{i} = 570.42i cm^{-1}

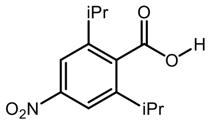

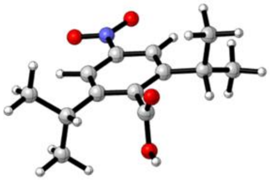

***Z*-3e**

6	-1.204834000	1.205076000	-0.076525000
6	0.202121000	1.231135000	-0.087894000
6	0.897431000	0.001427000	-0.005100000
6	0.226414000	-1.243066000	0.079494000
6	-1.179994000	-1.244229000	0.080462000
6	-1.862693000	-0.025688000	0.004788000
1	-1.798818000	2.119275000	-0.132483000
1	-1.756072000	-2.168868000	0.144156000
6	2.398255000	0.006547000	-0.042325000
8	3.080170000	-0.279038000	-1.007888000
8	2.920885000	0.369033000	1.157337000
1	3.888753000	0.350725000	1.025138000
6	1.006642000	-2.547476000	0.112924000
1	2.000396000	-2.331529000	0.546962000
6	0.942881000	2.557877000	-0.137726000
1	1.984279000	2.354319000	-0.445720000
6	0.343518000	3.522126000	-1.170536000
1	0.969424000	4.427297000	-1.248027000
1	0.283609000	3.054969000	-2.167436000
1	-0.669814000	3.846821000	-0.879050000
6	0.973238000	3.188608000	1.264268000
1	-0.054226000	3.397593000	1.611012000

1	1.454355000	2.508944000	1.985649000
1	1.531617000	4.140951000	1.249684000
6	1.212676000	-3.060849000	-1.322785000
1	0.235162000	-3.268492000	-1.792945000
1	1.745584000	-2.313269000	-1.931151000
1	1.799628000	-3.995860000	-1.318587000
6	0.345374000	-3.615310000	0.993377000
1	1.009391000	-4.491750000	1.080256000
1	0.139832000	-3.233331000	2.007124000
1	-0.604571000	-3.967212000	0.556379000
7	-3.343501000	-0.039995000	0.013280000
8	-3.899566000	-1.138719000	0.085161000
8	-3.921515000	1.047763000	-0.051918000

E = –858.752038 au   ZPVE = 0.288543219 au

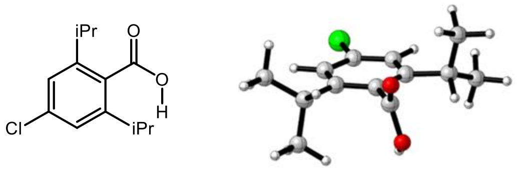

***E*-3f**

6	1.226155000	1.387597000	-0.077265000
6	1.235444000	-0.019657000	-0.113357000
6	-0.002056000	-0.701283000	-0.006561000
6	-1.233776000	-0.007307000	0.089586000
6	-1.211686000	1.400126000	0.104914000
6	0.011965000	2.079329000	0.033788000
1	2.157080000	1.955745000	-0.145817000
1	-2.138482000	1.975597000	0.169632000
6	-0.021836000	-2.209262000	-0.026782000
8	0.047269000	-2.894445000	-1.022666000
8	-0.128184000	-2.785497000	1.208583000
1	-0.171138000	-2.057936000	1.850723000
6	-2.550189000	-0.771755000	0.056459000
1	-2.383200000	-1.752516000	0.538311000
6	2.543337000	-0.793702000	-0.208001000
1	2.328061000	-1.730070000	-0.752640000
6	3.629709000	-0.047243000	-0.991642000
1	4.495550000	-0.711201000	-1.151955000
1	3.260947000	0.283974000	-1.976350000
1	3.993829000	0.836808000	-0.440292000
6	3.045086000	-1.160222000	1.200343000
1	3.225618000	-0.245233000	1.792018000
1	2.311077000	-1.784112000	1.735319000
1	3.990531000	-1.726888000	1.140751000
6	-2.946297000	-1.029922000	-1.408823000
1	-3.108711000	-0.069972000	-1.930100000
1	-2.154514000	-1.584843000	-1.937564000
1	-3.880286000	-1.616248000	-1.460609000
6	-3.682545000	-0.073609000	0.818690000

1	-4.564663000	-0.733888000	0.865280000
1	-3.384376000	0.178893000	1.850115000
1	-3.997358000	0.855067000	0.312717000
17	0.023970000	3.826343000	0.072061000

E = –1113.77052 au ZPVE = 0.276356064 au

***TS*-3f**

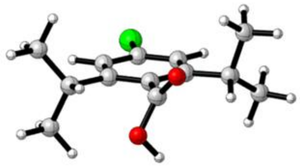

6	-1.160022000	1.397129000	0.061435000
6	-1.222157000	-0.008829000	0.073362000
6	-0.010596000	-0.740960000	-0.004912000
6	1.248650000	-0.089473000	-0.078195000
6	1.273698000	1.318177000	-0.092859000
6	0.078223000	2.044950000	-0.027443000
1	-2.072630000	1.996323000	0.123447000
1	2.221271000	1.858990000	-0.155716000
6	-0.052649000	-2.241702000	-0.003972000
8	0.500947000	-2.946990000	0.808471000
8	-0.830742000	-2.779941000	-1.029348000
1	-0.236041000	-2.920343000	-1.783311000
6	2.560476000	-0.863116000	-0.097558000
1	2.357016000	-1.880711000	-0.474734000
6	-2.579820000	-0.690806000	0.177060000
1	-2.418132000	-1.758462000	0.400722000
6	-3.424942000	-0.111007000	1.321578000
1	-4.355277000	-0.693616000	1.434750000
1	-2.877348000	-0.142756000	2.278230000
1	-3.713904000	0.935684000	1.125115000
6	-3.323269000	-0.602328000	-1.164316000
1	-3.503200000	0.452364000	-1.438831000
1	-2.732429000	-1.078403000	-1.962284000
1	-4.300690000	-1.111855000	-1.101297000
6	3.100155000	-1.002009000	1.335897000
1	4.043771000	-1.575457000	1.341564000
1	3.299388000	-0.003732000	1.764631000
1	2.368814000	-1.522729000	1.972955000
6	3.612241000	-0.235565000	-1.023073000
1	4.485126000	-0.905401000	-1.104010000
1	3.211380000	-0.063569000	-2.036132000
1	3.978849000	0.727867000	-0.629873000
17	0.134263000	3.791441000	-0.050014000

E = –1113.755603 au ZPVE = 0.274464749 au ν_i = 569.07*i* cm^{-1}

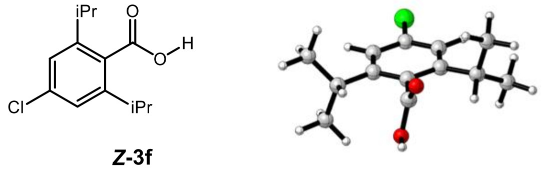

***Z*-3f**

6	1.209600000	1.379480000	-0.073759000
6	1.224878000	-0.028525000	-0.084905000
6	-0.008943000	-0.714681000	-0.001949000
6	-1.244471000	-0.028797000	0.082622000
6	-1.228371000	1.378324000	0.086123000
6	-0.008860000	2.065096000	0.010034000
1	2.140546000	1.949967000	-0.132145000
1	-2.158114000	1.949180000	0.151155000
6	-0.020220000	-2.214213000	-0.034129000
8	-0.388667000	-2.900164000	-0.968712000
8	0.430551000	-2.739636000	1.135524000
1	0.396249000	-3.706799000	1.002408000
6	-2.562228000	-0.788717000	0.118809000
1	-2.359362000	-1.791591000	0.537298000
6	2.549638000	-0.774557000	-0.147055000
1	2.337539000	-1.820927000	-0.430344000
6	3.493704000	-0.200780000	-1.213107000
1	4.393519000	-0.833488000	-1.300585000
1	3.003026000	-0.155955000	-2.199492000
1	3.832112000	0.815827000	-0.949376000
6	3.213395000	-0.784218000	1.239390000
1	3.433474000	0.247781000	1.565518000
1	2.548306000	-1.251000000	1.983313000
1	4.163714000	-1.346101000	1.213139000
6	-3.096444000	-0.971083000	-1.312058000
1	-3.290784000	0.014514000	-1.771220000
1	-2.364688000	-1.511854000	-1.932337000
1	-4.042194000	-1.541038000	-1.303857000
6	-3.611811000	-0.124803000	1.019678000
1	-4.493712000	-0.780961000	1.113227000
1	-3.213742000	0.068606000	2.029676000
1	-3.962244000	0.832050000	0.596317000
17	-0.009289000	3.813237000	0.020861000

E = –1113.776582 au   ZPVE = 0.276176749 au

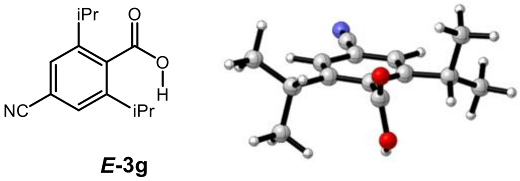

***E*-3g**

6	-1.229721000	1.472710000	0.074802000
6	-1.238801000	0.067113000	0.110227000
6	0.002308000	-0.610551000	0.003018000

6	1.236914000	0.081416000	-0.089664000
6	1.212991000	1.486997000	-0.104780000
6	-0.013689000	2.177721000	-0.034795000
1	-2.163039000	2.038166000	0.143139000
1	2.141248000	2.061236000	-0.169889000
6	0.023814000	-2.119694000	0.025688000
8	-0.029122000	-2.797806000	1.027102000
8	0.111976000	-2.700105000	-1.207506000
1	0.148152000	-1.978135000	-1.856407000
6	2.550553000	-0.686786000	-0.060045000
1	2.379156000	-1.667356000	-0.540601000
6	-2.543488000	-0.711396000	0.204765000
1	-2.323512000	-1.651693000	0.740537000
6	-3.627161000	0.027040000	1.000013000
1	-4.490137000	-0.640585000	1.159610000
1	-3.254241000	0.351112000	1.985505000
1	-3.996697000	0.914132000	0.457424000
6	-3.049756000	-1.066101000	-1.205074000
1	-3.234900000	-0.146632000	-1.788156000
1	-2.317535000	-1.684709000	-1.748507000
1	-3.993780000	-1.634761000	-1.145628000
6	2.948260000	-0.943653000	1.405241000
1	3.115842000	0.016463000	1.924436000
1	2.156357000	-1.495472000	1.936919000
1	3.880294000	-1.532880000	1.454871000
6	3.682231000	0.008687000	-0.825872000
1	4.562049000	-0.654288000	-0.874358000
1	3.382319000	0.261148000	-1.856771000
1	4.001284000	0.936431000	-0.321029000
6	-0.024575000	3.620881000	-0.067250000
7	-0.033567000	4.809258000	-0.096501000

E = –746.726484 au   ZPVE = 0.283805514 au

***TS*-3g**

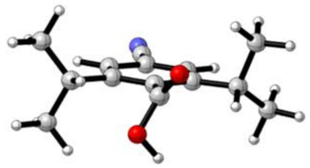

6	-1.170946000	1.476785000	0.062832000
6	-1.222161000	0.071984000	0.074939000
6	-0.001956000	-0.646491000	-0.005017000
6	1.254578000	0.010768000	-0.080160000
6	1.268549000	1.416893000	-0.093093000
6	0.065086000	2.146699000	-0.026030000
1	-2.090307000	2.066867000	0.123691000
1	2.213727000	1.963124000	-0.156178000
6	-0.036002000	-2.149375000	0.000037000
8	0.477052000	-2.845064000	0.845906000
8	-0.762170000	-2.691483000	-1.058024000

1	-0.136198000	-2.829627000	-1.786927000
6	2.565924000	-0.762364000	-0.099718000
1	2.362367000	-1.778470000	-0.482172000
6	-2.568952000	-0.630607000	0.170468000
1	-2.391470000	-1.696037000	0.394777000
6	-3.429240000	-0.063877000	1.310117000
1	-4.351439000	-0.660190000	1.416422000
1	-2.887907000	-0.088632000	2.270483000
1	-3.732312000	0.978404000	1.111846000
6	-3.303304000	-0.551769000	-1.176818000
1	-3.498430000	0.500265000	-1.450822000
1	-2.699625000	-1.017478000	-1.971302000
1	-4.272641000	-1.076875000	-1.120738000
6	3.099999000	-0.906539000	1.335592000
1	3.300128000	0.090111000	1.767412000
1	2.366160000	-1.427516000	1.969640000
1	4.042402000	-1.481563000	1.341868000
6	3.619625000	-0.130451000	-1.019860000
1	4.493183000	-0.799209000	-1.100054000
1	3.222240000	0.044633000	-2.033707000
1	3.983865000	0.831621000	-0.621427000
6	0.100320000	3.589572000	-0.044583000
7	0.129725000	4.777940000	-0.061158000

E = –746.711756 au   ZPVE = 0.281883203 au ν_i = 570.62*i* cm^{-1}

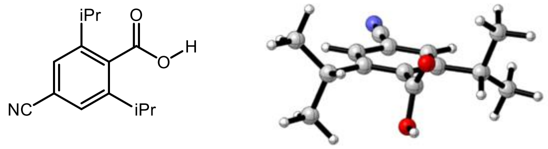

***Z*-3g**

6	-1.229465000	1.459371000	0.077292000
6	-1.229842000	0.053078000	0.091232000
6	0.014047000	-0.615576000	0.006324000
6	1.244584000	0.079344000	-0.082991000
6	1.213849000	1.484508000	-0.086112000
6	-0.015788000	2.170264000	-0.008315000
1	-2.168176000	2.018162000	0.134735000
1	2.139226000	2.063325000	-0.153156000
6	0.039156000	-2.116615000	0.045510000
8	0.311236000	-2.791584000	1.019592000
8	-0.278974000	-2.646977000	-1.163276000
1	-0.242950000	-3.614322000	-1.030176000
6	2.562071000	-0.679519000	-0.117770000
1	2.361133000	-1.673641000	-0.558491000
6	-2.538475000	-0.720055000	0.142493000
1	-2.308338000	-1.752664000	0.461740000
6	-3.523906000	-0.140012000	1.166423000
1	-4.407998000	-0.794565000	1.250191000
1	-3.061034000	-0.052398000	2.163237000

1	-3.883174000	0.857901000	0.862246000
6	-3.162632000	-0.781533000	-1.261517000
1	-3.397834000	0.236243000	-1.619910000
1	-2.467227000	-1.250959000	-1.975557000
1	-4.099627000	-1.365332000	-1.245211000
6	3.076818000	-0.886828000	1.317295000
1	3.270255000	0.090663000	1.793540000
1	2.336140000	-1.433060000	1.922327000
1	4.019900000	-1.460777000	1.311177000
6	3.622794000	-0.000376000	-0.993420000
1	4.505129000	-0.655572000	-1.087195000
1	3.238030000	0.210890000	-2.004923000
1	3.968173000	0.948146000	-0.547924000
6	-0.031414000	3.613805000	-0.018713000
7	-0.044357000	4.802472000	-0.027652000

E = –746.732956 au   ZPVE = 0.283654837 au

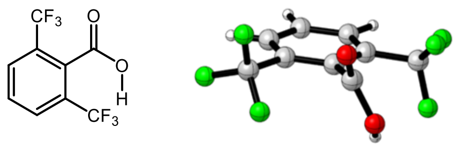

***E*-4a**

6	1.227506000	2.093738000	0.014189000
6	1.220849000	0.689493000	0.004832000
6	0.002695000	-0.026780000	-0.022780000
6	-1.206110000	0.706668000	-0.054935000
6	-1.197460000	2.111712000	-0.062170000
6	0.020078000	2.804231000	-0.033604000
1	2.180299000	2.626539000	0.055860000
1	-2.143681000	2.656326000	-0.098362000
6	0.024492000	-1.542718000	-0.144273000
8	0.488741000	-2.104588000	-1.106599000
8	-0.502583000	-2.223681000	0.898321000
1	-0.824388000	-1.586153000	1.554424000
6	-2.538168000	-0.007132000	-0.034344000
6	2.549408000	-0.038053000	0.051847000
1	0.027826000	3.898250000	-0.038760000
9	2.931096000	-0.461211000	-1.160329000
9	2.492995000	-1.106750000	0.871277000
9	3.524084000	0.769788000	0.520217000
9	-2.787673000	-0.505237000	1.205865000
9	-2.576914000	-1.036784000	-0.893578000
9	-3.555706000	0.816220000	-0.334508000

E = –1092.232597 au   ZPVE = 0.125429801 au
For deuterated acid (***E*-7a**): ZPVE = 0.121953466 au

***TS*-4a**

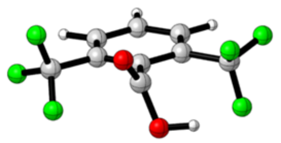

6	-1.236274000	2.094438000	-0.042095000
6	-1.225649000	0.690245000	-0.010026000
6	-0.007140000	-0.023199000	0.027484000
6	1.197973000	0.714979000	0.044286000
6	1.187513000	2.119553000	0.032977000
6	-0.031275000	2.809147000	-0.009352000
1	-2.190820000	2.623364000	-0.091380000
1	2.132804000	2.666688000	0.063078000
6	-0.014695000	-1.537840000	0.119798000
8	-0.484910000	-2.124011000	1.062335000
8	0.472411000	-2.165229000	-1.004608000
1	1.433995000	-2.252932000	-0.918013000
6	2.532770000	0.012780000	0.043113000
6	-2.551312000	-0.042359000	-0.038137000
1	-0.041662000	3.903004000	-0.022776000
9	-2.913949000	-0.459509000	1.184537000
9	-2.501180000	-1.117807000	-0.847132000
9	-3.536934000	0.756679000	-0.496860000
9	2.865818000	-0.411785000	-1.199154000
9	2.522083000	-1.089182000	0.830939000
9	3.525739000	0.804639000	0.472002000

E = –1092.219459 au   ZPVE = 0.123748861 au ν_i = 562.78i cm^{-1}
For deuterated acid (***TS*-7a**): ZPVE = 0.120524191 au ν_i = 416.45i cm^{-1}

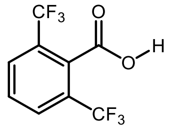

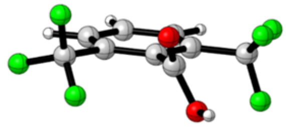

***Z*-4a**

6	1.226308000	2.100601000	0.040482000
6	1.225076000	0.696622000	0.013372000
6	0.009214000	-0.021230000	-0.014885000
6	-1.204628000	0.700128000	-0.028113000
6	-1.199661000	2.105402000	-0.016518000
6	0.014203000	2.804017000	0.019093000
1	2.176739000	2.637509000	0.080274000
1	-2.148141000	2.646851000	-0.042185000
6	0.026211000	-1.528979000	-0.093314000
8	0.473407000	-2.158677000	-1.026731000
8	-0.517920000	-2.071923000	1.015812000
1	-0.497522000	-3.035630000	0.863506000
6	-2.542908000	-0.007148000	-0.041334000
6	2.554250000	-0.028384000	0.026984000
1	0.015772000	3.898079000	0.032469000

9	2.899241000	-0.458758000	-1.195963000
9	2.525977000	-1.096847000	0.849384000
9	3.544119000	0.779133000	0.460805000
9	-2.915424000	-0.385370000	1.193427000
9	-2.522996000	-1.106982000	-0.820133000
9	-3.513043000	0.799027000	-0.518777000

E = –1092.239285 au   ZPVE = 0.125541912 au

For deuterated acid (***Z*-7a**): ZPVE = 0.122089179 au

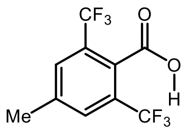

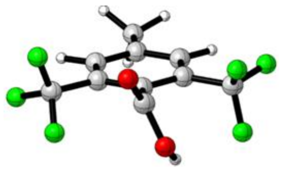

***E*-4b**

6	1.231208000	1.813394000	0.017726000
6	1.218372000	0.410652000	0.007813000
6	-0.002020000	-0.301689000	-0.018899000
6	-1.201355000	0.445485000	-0.051537000
6	-1.178245000	1.850022000	-0.058140000
6	0.036686000	2.554889000	-0.030611000
1	2.191309000	2.335945000	0.056048000
1	-2.123297000	2.398550000	-0.098785000
6	0.008695000	-1.816547000	-0.141760000
8	0.478705000	-2.383963000	-1.098167000
8	-0.535558000	-2.493363000	0.895461000
1	-0.858019000	-1.852363000	1.547795000
6	-2.541196000	-0.252444000	-0.036539000
6	2.544230000	-0.320991000	0.051143000
9	2.922861000	-0.744745000	-1.162229000
9	2.486933000	-1.390278000	0.869903000
9	3.524459000	0.482386000	0.517927000
9	-2.801924000	-0.749652000	1.202363000
9	-2.589561000	-1.281149000	-0.896931000
9	-3.549883000	0.581792000	-0.339177000
6	0.064584000	4.064065000	-0.008811000
1	-0.874115000	4.484746000	-0.401454000
1	0.197476000	4.433771000	1.022686000
1	0.899749000	4.451766000	-0.613655000

E = –1131.419433 au   ZPVE = 0.152943044 au

***TS*-4b**

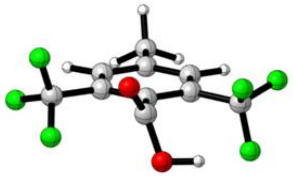

6	-1.242192000	1.811494000	-0.044406000
6	-1.223019000	0.408687000	-0.009322000

6	-0.001200000	-0.298650000	0.030500000
6	1.193244000	0.455344000	0.045200000
6	1.166013000	1.859181000	0.031690000
6	-0.051524000	2.559002000	-0.013257000
1	-2.204388000	2.328369000	-0.100063000
1	2.109513000	2.411939000	0.057642000
6	0.004370000	-1.812185000	0.121864000
8	-0.473112000	-2.404290000	1.057269000
8	0.512267000	-2.436253000	-0.995948000
1	1.473578000	-2.511801000	-0.896917000
6	2.537195000	-0.227726000	0.042305000
6	-2.544602000	-0.330289000	-0.039924000
9	-2.907485000	-0.750821000	1.181936000
9	-2.489222000	-1.404941000	-0.849966000
9	-3.535570000	0.463185000	-0.498850000
9	2.879792000	-0.641665000	-1.200906000
9	2.541124000	-1.334315000	0.825079000
9	3.519775000	0.574674000	0.477606000
6	-0.084980000	4.068135000	0.001808000
1	-0.218648000	4.440969000	1.032128000
1	0.852477000	4.491083000	-0.391781000
1	-0.921317000	4.450770000	-0.604424000

E = –1131.406212 au   ZPVE = 0.151268558 au ν_i = 560.48i cm^{-1}

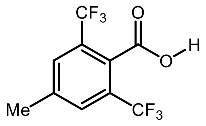

***Z*-4b**

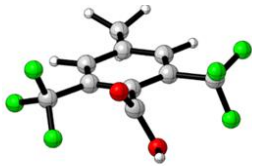

6	-1.225119000	1.825078000	-0.023492000
6	-1.222331000	0.422040000	-0.007624000
6	-0.006890000	-0.296046000	0.013344000
6	1.200351000	0.434921000	0.033842000
6	1.185696000	1.839421000	0.033925000
6	-0.023429000	2.554843000	0.004325000
1	-2.180973000	2.355494000	-0.054684000
1	2.134923000	2.381367000	0.069476000
6	-0.017749000	-1.803155000	0.081154000
8	-0.470462000	-2.443237000	1.005055000
8	0.539013000	-2.336527000	-1.026971000
1	0.522699000	-3.301008000	-0.879518000
6	2.543697000	-0.261538000	0.045953000
6	-2.551167000	-0.302397000	-0.023415000
9	-2.895051000	-0.741423000	1.197146000
9	-2.525137000	-1.365418000	-0.853247000
9	-3.543447000	0.507286000	-0.449899000
9	2.924062000	-0.628006000	-1.190377000
9	2.530660000	-1.367769000	0.816389000
9	3.507813000	0.547438000	0.532533000

6	-0.033701000	4.064001000	-0.036639000
1	0.872579000	4.479820000	0.430825000
1	-0.070422000	4.421865000	-1.080256000
1	-0.913446000	4.468595000	0.488356000

E = –1131.425973 au ZPVE = 0.15305388 au

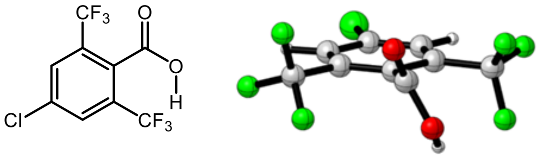

***E*-4c**

6	-1.288172000	1.533961000	-0.029575000
6	-1.220857000	0.133023000	-0.012015000
6	0.022144000	-0.539127000	0.017524000
6	1.199301000	0.243718000	0.047266000
6	1.143407000	1.646066000	0.048576000
1	-2.254700000	2.039786000	-0.074594000
1	2.060885000	2.236765000	0.082743000
6	0.057586000	-2.053694000	0.151216000
8	-0.392301000	-2.623178000	1.115825000
8	0.617649000	-2.722862000	-0.880833000
1	0.914559000	-2.081017000	-1.544462000
6	-2.525123000	-0.641116000	-0.054720000
6	2.561751000	-0.414318000	0.031973000
6	-0.104105000	2.282905000	0.016151000
17	-0.183017000	4.016776000	0.016372000
9	-2.886696000	-1.071441000	1.160007000
9	-3.526673000	0.130847000	-0.524281000
9	-2.429045000	-1.708680000	-0.870177000
9	3.542955000	0.453759000	0.322783000
9	2.827277000	-0.912878000	-1.203421000
9	2.640202000	-1.431708000	0.901722000

E = –1551.282849 au ZPVE = 0.115652845 au

***TS*-4c**

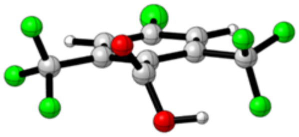

6	-1.321239000	1.511437000	-0.034368000
6	-1.225951000	0.111905000	-0.006899000
6	0.028716000	-0.536236000	0.026858000
6	1.188290000	0.270927000	0.047106000
6	1.106523000	1.671720000	0.042678000
1	-2.297818000	1.997180000	-0.082154000
1	2.012479000	2.280379000	0.075640000

6	0.104292000	-2.049397000	0.113608000
8	-0.343647000	-2.662737000	1.049645000
8	0.633996000	-2.644893000	-1.007756000
1	1.598808000	-2.676495000	-0.918262000
6	-2.513834000	-0.688797000	-0.036901000
6	2.564009000	-0.351487000	0.045245000
6	-0.152784000	2.284090000	0.002177000
17	-0.265276000	4.016512000	-0.010805000
9	-2.846980000	-1.131955000	1.183730000
9	-3.539639000	0.062199000	-0.486080000
9	-2.407605000	-1.752139000	-0.854715000
9	2.917679000	-0.753918000	-1.197256000
9	2.614460000	-1.451315000	0.832879000
9	3.507119000	0.497606000	0.474367000

E = –1551.269738 au   ZPVE = 0.113974996 au ν_{i} = 563.57*i* cm^{-1}

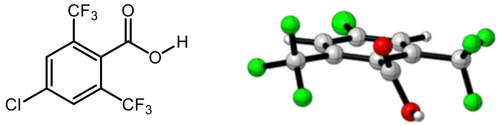

***Z*-4c**

6	-1.263442000	1.561739000	-0.028980000
6	-1.224774000	0.159716000	-0.008354000
6	0.004512000	-0.534822000	0.015040000
6	1.200015000	0.216216000	0.032248000
6	1.171068000	1.619678000	0.029839000
1	-2.218989000	2.088251000	-0.066647000
1	2.100492000	2.191700000	0.059729000
6	0.019921000	-2.042664000	0.085931000
8	-0.426008000	-2.685647000	1.010751000
8	0.590296000	-2.566585000	-1.018410000
1	0.591539000	-3.531524000	-0.872184000
6	-2.542705000	-0.589206000	-0.025813000
6	2.557715000	-0.457134000	0.043849000
6	-0.062261000	2.283138000	-0.002917000
17	-0.103162000	4.019284000	-0.014460000
9	-2.874598000	-1.034184000	1.194256000
9	-3.545951000	0.204704000	-0.451014000
9	-2.493666000	-1.648330000	-0.857473000
9	3.505928000	0.373762000	0.520225000
9	2.935301000	-0.824606000	-1.191295000
9	2.562553000	-1.555608000	0.823205000

E = –1551.2898 au   ZPVE = 0.115778805 au

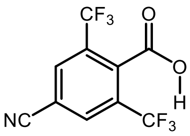

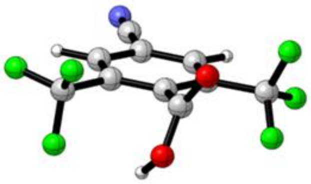

***E*-4d**

6	-1.270246000	1.634511000	0.027268000
6	-1.223261000	0.234251000	0.011910000
6	0.012046000	-0.452838000	-0.016815000
6	1.202462000	0.310285000	-0.047734000
6	1.166524000	1.711589000	-0.051966000
6	-0.073870000	2.374682000	-0.019599000
1	-2.230916000	2.152373000	0.071598000
1	2.094065000	2.287185000	-0.086680000
6	0.025601000	-1.968698000	-0.155725000
8	-0.423376000	-2.520989000	-1.130508000
8	0.563051000	-2.652317000	0.876982000
1	0.866213000	-2.021428000	1.548473000
6	2.554107000	-0.372456000	-0.028616000
6	-2.537048000	-0.525968000	0.056410000
6	-0.118229000	3.817477000	-0.020594000
7	-0.153866000	5.005240000	-0.020087000
9	3.549465000	0.480011000	-0.312754000
9	2.616055000	-1.387608000	-0.901081000
9	2.801423000	-0.877375000	1.206706000
9	-2.902243000	-0.950523000	-1.158086000
9	-2.448758000	-1.593947000	0.871406000
9	-3.526857000	0.257735000	0.528051000

E = –1184.235653 au   ZPVE = 0.123053386 au
For deuterated acid (***E*-7d**): ZPVE = 0.119583745 au

***TS*-4d**

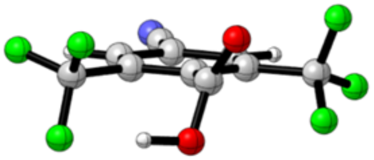

6	1.294472000	1.621788000	-0.034157000
6	1.228854000	0.222113000	-0.007241000
6	-0.013707000	-0.449278000	0.027042000
6	-1.192214000	0.330422000	0.047357000
6	-1.139888000	1.730779000	0.043843000
6	0.108487000	2.378077000	0.002885000
1	2.262172000	2.126044000	-0.081577000
1	-2.060193000	2.318365000	0.076483000
6	-0.058158000	-1.965078000	0.115567000
8	0.383278000	-2.563154000	1.064100000
8	-0.549819000	-2.570921000	-1.015492000
1	-1.515595000	-2.627293000	-0.948123000
6	-2.552795000	-0.327700000	0.043792000
6	2.531257000	-0.556964000	-0.036669000

6	0.170993000	3.820294000	-0.007844000
7	0.220741000	5.007387000	-0.016704000
9	-2.577376000	-1.419888000	0.840599000
9	-2.884044000	-0.749120000	-1.198205000
9	-3.517927000	0.501903000	0.458267000
9	2.868400000	-0.991345000	1.185079000
9	2.440009000	-1.622359000	-0.852625000
9	3.541711000	0.211593000	-0.487133000

E = –1184.223076 au   ZPVE = 0.121248287 au ν_i = 565.38i cm^{-1}
For deuterated acid (***TS*-7d**): ZPVE = 0.118181801 au ν_i = 418.35i cm^{-1}

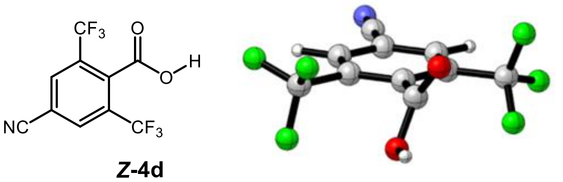

***Z*-4d**

6	-1.252787000	1.654877000	0.029159000
6	-1.226671000	0.254140000	0.008924000
6	-0.001449000	-0.448389000	-0.014155000
6	1.202746000	0.289310000	-0.031994000
6	1.185817000	1.691394000	-0.030751000
6	-0.043589000	2.374304000	0.002206000
1	-2.205243000	2.187938000	0.066568000
1	2.121886000	2.253388000	-0.060149000
6	0.000505000	-1.957847000	-0.088683000
8	-0.442417000	-2.589548000	-1.022280000
8	0.554171000	-2.488309000	1.019428000
1	0.546448000	-3.453731000	0.875549000
6	2.553572000	-0.400931000	-0.041554000
6	-2.549961000	-0.488039000	0.026925000
6	-0.064610000	3.817932000	0.011268000
7	-0.081885000	5.005957000	0.019094000
9	3.510617000	0.420290000	-0.513900000
9	2.544913000	-1.497149000	-0.822784000
9	2.919554000	-0.773781000	1.194384000
9	-2.881561000	-0.929275000	-1.193489000
9	-2.503616000	-1.546746000	0.858218000
9	-3.546060000	0.312555000	0.453028000

E = –1184.243001 au   ZPVE = 0.123208207 au
For deuterated acid (***Z*-7d**): ZPVE = 0.119757769 au

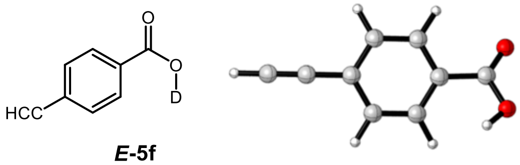

***E*-5f**

6	1.107921000	-1.220389000	0.130461000
6	-0.290740000	-1.231273000	0.113880000
6	-1.011963000	-0.026437000	-0.007792000
6	-0.310890000	1.188677000	-0.154953000
6	1.090593000	1.204061000	-0.152783000
6	1.814934000	-0.000506000	-0.001057000
1	1.669266000	-2.153738000	0.238358000
1	-0.848984000	-2.168109000	0.198981000
1	-0.847713000	2.131225000	-0.314081000
1	1.635090000	2.145171000	-0.277321000
6	-2.514114000	-0.109882000	-0.026362000
8	-3.117730000	-1.123078000	-0.303705000
8	-3.182400000	1.035042000	0.287454000
1(iso=2)	-2.540138000	1.663008000	0.650600000
6	3.253756000	0.013531000	0.010801000
6	4.488624000	0.024461000	0.021674000
1	5.564795000	0.033281000	0.030252000

E = –495.540988 au   ZPVE = 0.119992367 au

***TS*-5f**

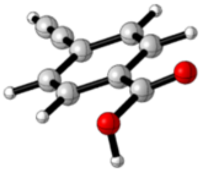

6	-1.124752000	1.230769000	-0.041176000
6	0.273402000	1.253442000	-0.031673000
6	1.002999000	0.046811000	0.011613000
6	0.318528000	-1.184617000	0.059290000
6	-1.082110000	-1.210123000	0.053432000
6	-1.818399000	-0.003459000	0.000468000
1	-1.696262000	2.163505000	-0.078200000
1	0.821671000	2.199678000	-0.059237000
1	0.888676000	-2.115276000	0.115359000
1	-1.620088000	-2.162464000	0.094026000
6	2.497744000	0.119433000	0.017476000
8	3.118364000	1.157275000	0.081514000
8	3.136600000	-1.116319000	-0.004200000
1(iso=2)	3.225491000	-1.371177000	-0.935225000
6	-3.257166000	-0.029702000	-0.008235000
6	-4.492096000	-0.051229000	-0.016620000
1	-5.568092000	-0.069860000	-0.022695000

E = –495.531925 au   ZPVE = 0.118897833 au ν_i = 392.85i cm^{-1}

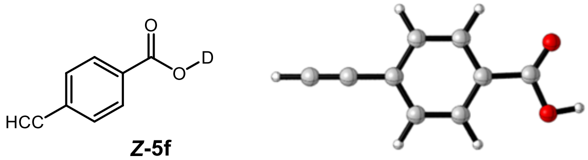

***Z*-5f**

6	-1.122443000	1.227414000	0.000030000
6	0.276543000	1.241022000	0.000007000
6	0.995627000	0.029420000	-0.000026000
6	0.306661000	-1.200073000	-0.000061000
6	-1.093857000	-1.215134000	-0.000043000
6	-1.823230000	-0.002592000	0.000036000
1	-1.687925000	2.164518000	0.000007000
1	0.832718000	2.182906000	0.000011000
1	0.869405000	-2.136940000	-0.000180000
1	-1.637686000	-2.164994000	-0.000075000
6	2.487567000	0.105651000	-0.000004000
8	3.128270000	1.140969000	-0.000066000
8	3.067974000	-1.122111000	0.000094000
1(iso=2)	4.024635000	-0.938956000	0.000175000
6	-3.262356000	-0.020055000	0.000034000
6	-4.497447000	-0.034129000	-0.000008000
1	-5.573490000	-0.046534000	0.000042000

E = –495.55223 au ZPVE = 0.120263713 au

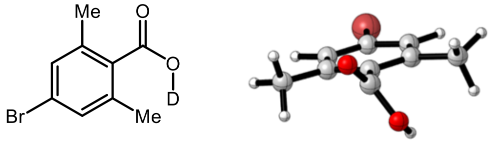

***E*-6d**

6	-0.588773000	1.227298000	0.071283000
6	0.816742000	1.253422000	0.046263000
6	1.518855000	0.024929000	-0.023666000
6	0.833651000	-1.211598000	-0.113083000
6	-0.574379000	-1.207869000	-0.105514000
6	-1.271525000	0.003610000	-0.004498000
1	-1.151172000	2.163721000	0.138863000
1	-1.124203000	-2.150719000	-0.187870000
6	3.024838000	0.058159000	-0.058753000
8	3.679119000	0.658119000	-0.882040000
8	3.636340000	-0.649270000	0.934114000
1(iso=2)	2.937023000	-0.967838000	1.526099000
6	1.570334000	-2.531782000	-0.233049000
1	2.464255000	-2.442456000	-0.870473000
1	1.899308000	-2.914970000	0.748920000
1	0.909655000	-3.293516000	-0.675595000
6	1.551023000	2.574986000	0.085472000
1	2.305763000	2.591310000	0.889522000

1	2.086780000	2.747374000	-0.861031000
1	0.846379000	3.403365000	0.255927000
35	-3.170916000	-0.010114000	0.018066000

E = –3069.914946 au   ZPVE = 0.15730145 au

***TS*-6d**

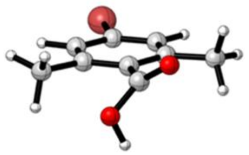

6	0.572001000	-1.205618000	-0.107265000
6	-0.835208000	-1.213850000	-0.112431000
6	-1.524507000	0.025701000	-0.031792000
6	-0.813358000	1.254451000	0.035687000
6	0.592644000	1.224546000	0.068866000
6	1.273920000	0.002489000	-0.004157000
1	1.120194000	-2.149507000	-0.186316000
1	1.155157000	2.160379000	0.141540000
6	-3.024094000	0.075709000	-0.047794000
8	-3.671825000	0.897741000	-0.656326000
8	-3.642320000	-0.933371000	0.686589000
1(iso=2)	-3.747953000	-0.592474000	1.588281000
6	-1.508045000	2.598948000	0.075935000
1	-1.976619000	2.827507000	-0.893571000
1	-2.312448000	2.625095000	0.828709000
1	-0.782017000	3.390703000	0.317819000
6	-1.539907000	-2.549582000	-0.236277000
1	-2.378367000	-2.506579000	-0.948344000
1	-0.827846000	-3.313777000	-0.585876000
1	-1.957087000	-2.872469000	0.729814000
35	3.173128000	-0.016017000	0.026320000

E = –3069.901752 au   ZPVE = 0.156455681 au ν_i = 417.14*i* cm^{-1}

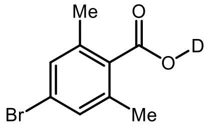

***Z*-6d**

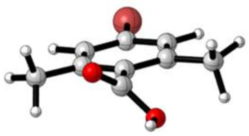

6	-0.594169000	1.222541000	0.073545000
6	0.811973000	1.248821000	0.050955000
6	1.517455000	0.018750000	-0.016362000
6	0.828504000	-1.219241000	-0.095075000
6	-0.578968000	-1.208751000	-0.093298000
6	-1.277831000	0.001818000	-0.001565000
1	-1.154719000	2.159809000	0.142162000
1	-1.129059000	-2.151915000	-0.166535000
6	3.014568000	0.072734000	-0.047316000
8	3.674977000	0.866777000	-0.692076000

8 3.583832000 -0.862760000 0.756798000
1(iso=2) 4.543326000 -0.734505000 0.633751000
6 1.535370000 -2.555165000 -0.198651000
1 2.350575000 -2.528552000 -0.938858000
1 1.980970000 -2.844760000 0.765622000
1 0.819483000 -3.334361000 -0.503922000
6 1.516023000 2.587635000 0.105071000
1 2.306457000 2.601099000 0.872654000
1 2.002229000 2.814805000 -0.855912000
1 0.792683000 3.384500000 0.338083000
35 -3.177713000 -0.011803000 0.015182000

E = –3069.92255 au   ZPVE = 0.157857673 au

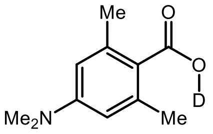

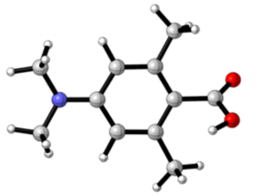

***E*-6h**

6 -0.867985000 1.220874000 -0.023065000
6 0.533948000 1.246914000 -0.003026000
6 1.250543000 0.025767000 -0.037604000
6 0.550724000 -1.201196000 -0.137457000
6 -0.853669000 -1.191755000 -0.178615000
6 -1.598932000 0.010404000 -0.125515000
1 -1.391911000 2.177998000 0.028140000
1 -1.364080000 -2.153253000 -0.271800000
6 2.752223000 0.065198000 -0.011076000
8 3.440783000 0.756637000 -0.729949000
8 3.336438000 -0.748087000 0.919916000
1(iso=2) 2.617260000 -1.119382000 1.453685000
6 1.264447000 -2.538934000 -0.226529000
1 2.197389000 -2.471733000 -0.807567000
1 1.523029000 -2.945254000 0.768367000
1 0.611701000 -3.279960000 -0.714943000
6 1.250145000 2.578894000 0.059765000
1 1.988936000 2.604612000 0.878110000
1 1.804792000 2.767674000 -0.872458000
1 0.528829000 3.395838000 0.220026000
7 -2.993966000 0.006917000 -0.205023000
6 -3.674103000 -1.247599000 0.082626000
1 -4.758855000 -1.085358000 0.003100000
1 -3.402665000 -2.016099000 -0.658364000
1 -3.446360000 -1.644200000 1.094427000
6 -3.690417000 1.208562000 0.231974000
1 -3.422935000 2.065873000 -0.405519000
1 -4.772613000 1.045381000 0.124324000
1 -3.474064000 1.478277000 1.287020000

E = –631.535655 au ZPVE = 0.241485371 au

***TS*-6h**

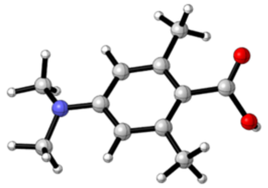

6	0.850067000	-1.192659000	-0.167828000
6	-0.552730000	-1.205962000	-0.126493000
6	-1.256355000	0.026185000	-0.043816000
6	-0.528113000	1.246888000	-0.016105000
6	0.874303000	1.215885000	-0.028012000
6	1.601930000	0.005551000	-0.120655000
1	1.359224000	-2.155639000	-0.248660000
1	1.399962000	2.171777000	0.025182000
6	-2.748525000	0.085808000	-0.006896000
8	-3.416066000	0.971930000	-0.496116000
8	-3.363321000	-0.992260000	0.635939000
1(iso=2)	-3.438585000	-0.740129000	1.568775000
6	-1.203604000	2.601479000	0.046940000
1	-1.713171000	2.835024000	-0.900426000
1	-1.975008000	2.643718000	0.832674000
1	-0.455750000	3.384249000	0.252391000
6	-1.236084000	-2.557955000	-0.210599000
1	-1.642407000	-2.869082000	0.764099000
1	-2.080366000	-2.550430000	-0.916471000
1	-0.511312000	-3.317490000	-0.546010000
7	2.995734000	-0.005512000	-0.193498000
6	3.697755000	1.201064000	0.219082000
1	4.779056000	1.032795000	0.110144000
1	3.429354000	2.047604000	-0.432353000
1	3.486461000	1.490161000	1.269971000
6	3.672560000	-1.258640000	0.108821000
1	3.398863000	-2.035241000	-0.622684000
1	4.757720000	-1.099862000	0.027469000
1	3.443690000	-1.642087000	1.125148000

E = –631.522534 au ZPVE = 0.240684239 au ν_i = 413.28*i* cm^{-1}

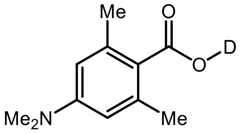

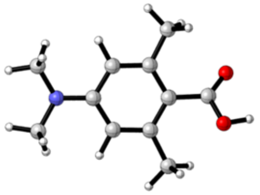

***Z*-6h**

6	-0.875754000	1.213803000	-0.027520000

6 0.526748000 1.241156000 -0.003176000
6 1.248364000 0.018850000 -0.030628000
6 0.544798000 -1.211239000 -0.113174000
6 -0.858253000 -1.194878000 -0.159849000
6 -1.606232000 0.005668000 -0.123847000
1 -1.398615000 2.171391000 0.021828000
1 -1.369397000 -2.157225000 -0.234620000
6 2.739347000 0.080417000 -0.001800000
8 3.423614000 0.950125000 -0.514182000
8 3.301812000 -0.945521000 0.696809000
1(iso=2) 4.259978000 -0.786044000 0.610562000
6 1.229337000 -2.563271000 -0.172721000
1 2.056027000 -2.572580000 -0.900058000
1 1.658068000 -2.840636000 0.802742000
1 0.501819000 -3.335815000 -0.469365000
6 1.209855000 2.591233000 0.071901000
1 1.972334000 2.622463000 0.866464000
1 1.730126000 2.825606000 -0.869355000
1 0.464747000 3.377597000 0.273629000
7 -3.001047000 -0.000964000 -0.208631000
6 -3.681799000 -1.250477000 0.099992000
1 -4.766200000 -1.090838000 0.009491000
1 -3.404242000 -2.033529000 -0.623099000
1 -3.460728000 -1.625834000 1.121181000
6 -3.699915000 1.206594000 0.206963000
1 -3.424647000 2.055305000 -0.438675000
1 -4.781416000 1.043565000 0.091324000
1 -3.492903000 1.489359000 1.260511000

E = –631.542934 au ZPVE = 0.242065657 au

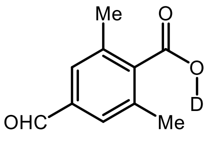

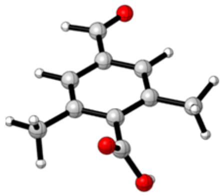

***E*-6i**

6 1.125311000 1.417450000 -0.084281000
6 -0.275646000 1.310139000 -0.049933000
6 -0.842886000 0.013516000 0.021615000
6 -0.038208000 -1.153877000 0.101019000
6 1.357331000 -1.003489000 0.080750000
6 1.939845000 0.272918000 -0.018893000
1 1.590478000 2.409148000 -0.151940000
1 2.017043000 -1.875297000 0.151459000
6 -2.344771000 -0.108895000 0.070953000
8 -3.043238000 0.372657000 0.934507000
8 -2.893670000 -0.816402000 -0.956895000
1(iso=2) -2.171604000 -1.038329000 -1.565779000
6 -0.653575000 -2.533823000 0.219824000

1	-1.521244000	-2.532967000	0.899200000
1	-0.997191000	-2.920918000	-0.755117000
1	0.089192000	-3.245176000	0.612702000
6	-1.150060000	2.543140000	-0.078430000
1	-1.906187000	2.481256000	-0.879117000
1	-1.696404000	2.651724000	0.871686000
1	-0.541718000	3.444980000	-0.247417000
6	3.423179000	0.408276000	-0.045533000
8	4.199510000	-0.534921000	0.007617000
1	3.793696000	1.462780000	-0.120056000

E = –611.025892 au   ZPVE = 0.176619793 au

***TS*-6i**

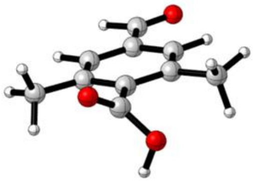

6	-1.130185000	1.412738000	0.093252000
6	0.271406000	1.310257000	0.047091000
6	0.847745000	0.012966000	-0.029431000
6	0.039403000	-1.156513000	-0.105039000
6	-1.355351000	-1.002759000	-0.084588000
6	-1.943532000	0.269733000	0.025760000
1	-1.595738000	2.403403000	0.171814000
1	-2.012681000	-1.876132000	-0.156389000
6	2.345911000	-0.092057000	-0.061450000
8	3.063294000	0.625288000	-0.721617000
8	2.865304000	-1.119074000	0.720820000
1(iso=2)	3.017956000	-0.745861000	1.602877000
6	0.622140000	-2.548299000	-0.233277000
1	-0.157493000	-3.246112000	-0.576839000
1	1.456086000	-2.579598000	-0.951879000
1	1.016261000	-2.905780000	0.730330000
6	1.108809000	2.570443000	0.079154000
1	1.917548000	2.510717000	0.825670000
1	1.590889000	2.746163000	-0.894825000
1	0.476090000	3.437291000	0.325920000
6	-3.426145000	0.402498000	0.061437000
8	-4.202683000	-0.540489000	0.003011000
1	-3.797431000	1.456063000	0.148155000

E = –611.012413 au   ZPVE = 0.175732637 au ν_i = 419.87i cm^{-1}

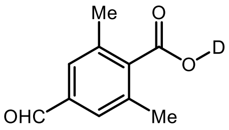

***Z*-6i**

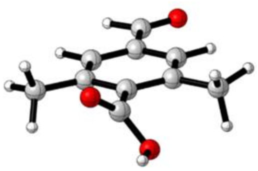

6	-1.133889000	1.411542000	0.091177000
6	0.267608000	1.306140000	0.058775000
6	0.838967000	0.008526000	-0.014069000
6	0.032304000	-1.160044000	-0.088320000
6	-1.362582000	-1.005164000	-0.074906000
6	-1.948676000	0.269344000	0.021482000
1	-1.598162000	2.403271000	0.163397000
1	-2.020893000	-1.878109000	-0.141834000
6	2.334809000	-0.091934000	-0.059984000
8	3.060053000	0.582834000	-0.767306000
8	2.814290000	-1.020496000	0.806462000
1(iso=2)	3.780810000	-1.002931000	0.672122000
6	0.620004000	-2.551246000	-0.195380000
1	1.426563000	-2.596078000	-0.944831000
1	1.048876000	-2.876123000	0.765306000
1	-0.163703000	-3.266014000	-0.490922000
6	1.115229000	2.558476000	0.105179000
1	1.902392000	2.488840000	0.873542000
1	1.622626000	2.725253000	-0.857357000
1	0.485871000	3.432563000	0.334227000
6	-3.431060000	0.404909000	0.044327000
8	-4.209316000	-0.536528000	-0.017626000
1	-3.800887000	1.459539000	0.124423000

E = –611.033372 au   ZPVE = 0.177138343 au

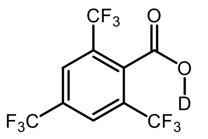

***E*-7b**

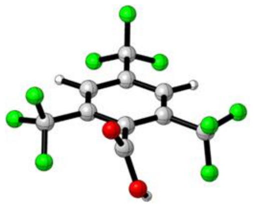

6	1.171649000	1.195615000	-0.058017000
6	-0.229597000	1.217304000	-0.021983000
6	-0.972773000	0.015778000	0.018378000
6	-0.265236000	-1.208036000	0.037892000
6	1.137172000	-1.233697000	0.020480000
6	1.850650000	-0.029476000	-0.017796000
1	1.730066000	2.131831000	-0.117967000
1	1.666834000	-2.187961000	0.039232000
6	-2.485788000	0.072266000	0.172391000
8	-3.008994000	0.545498000	1.151775000
8	-3.203143000	-0.432449000	-0.854590000
1(iso=2)	-2.592244000	-0.765543000	-1.530296000
6	-1.008584000	-2.526066000	0.022887000
6	-0.927812000	2.563922000	-0.062157000
6	3.359165000	-0.048268000	0.014050000
9	3.848911000	-1.178344000	-0.527791000
9	3.810968000	0.023544000	1.279557000

9	3.876741000	0.993943000	-0.660911000
9	-1.321468000	2.953254000	1.155929000
9	-0.104497000	3.515667000	-0.545922000
9	-2.009046000	2.525174000	-0.864275000
9	-1.536607000	-2.750031000	-1.208058000
9	-2.018364000	-2.544728000	0.904297000
9	-0.200040000	-3.560565000	0.297708000

E = –1428.54178 au   ZPVE = 0.126805418 au

***TS*-7b**

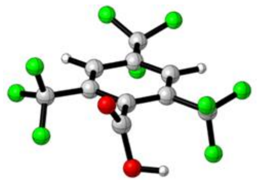

6	-1.179372000	-1.196831000	-0.004325000
6	0.222664000	-1.221752000	0.003228000
6	0.970242000	-0.023889000	0.028429000
6	0.265413000	1.200223000	0.058385000
6	-1.136334000	1.231359000	0.072978000
6	-1.854453000	0.029711000	0.037287000
1	-1.740718000	-2.132626000	-0.036635000
1	-1.662670000	2.186753000	0.120370000
6	2.486494000	-0.072503000	0.096882000
8	3.070895000	-0.550592000	1.036179000
8	3.105858000	0.384928000	-1.041960000
1(iso=2)	3.223262000	1.344881000	-0.970805000
6	1.005169000	2.516912000	0.052055000
6	0.917555000	-2.569502000	-0.029093000
6	-3.361946000	0.058364000	-0.008054000
9	-3.852116000	1.141304000	0.622594000
9	-3.892492000	-1.034392000	0.569255000
9	-3.801403000	0.097854000	-1.280186000
9	1.343910000	-2.931560000	1.188982000
9	0.082182000	-3.531787000	-0.467529000
9	1.978309000	-2.549659000	-0.856703000
9	1.430214000	2.830239000	-1.193934000
9	2.108512000	2.472240000	0.833670000
9	0.242162000	3.529512000	0.482372000

E = –1428.529096 au   ZPVE = 0.125393211 au ν_i = 418.29*i* cm^{-1}

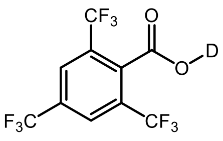

***Z*-7b**

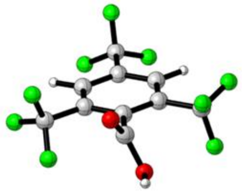

6	1.167264000	1.208636000	-0.052467000
6	-0.234029000	1.222657000	-0.016427000
6	-0.968024000	0.016769000	0.015780000
6	-0.261745000	-1.205475000	0.025201000
6	1.141148000	-1.221774000	0.006813000
6	1.849762000	-0.014703000	-0.029744000
1	1.721744000	2.147543000	-0.103628000
1	1.675967000	-2.173284000	0.022060000
6	-2.475915000	0.054188000	0.103645000
8	-3.088758000	0.509600000	1.043872000
8	-3.031210000	-0.480754000	-1.001998000
1(iso=2)	-3.994476000	-0.447419000	-0.848247000
6	-0.985424000	-2.537234000	0.035770000
6	-0.941041000	2.563749000	-0.032273000
6	3.357924000	-0.027864000	-0.003140000
9	3.851723000	-1.150682000	-0.556158000
9	3.814834000	0.034752000	1.261857000
9	3.870179000	1.022739000	-0.669857000
9	-1.362690000	2.912185000	1.191099000
9	-0.119274000	3.538131000	-0.469385000
9	-2.009601000	2.544216000	-0.852891000
9	-1.375782000	-2.892780000	-1.198520000
9	-2.077096000	-2.504268000	0.823547000
9	-0.185960000	-3.516659000	0.500960000

E = –1428.548931 au   ZPVE = 0.126957578 au

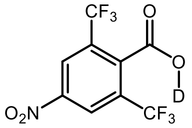

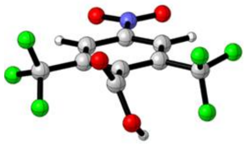

***E*-7c**

6	-1.486159000	1.132762000	0.029138000
6	-0.086812000	1.212556000	0.012327000
6	0.711343000	0.045753000	-0.015754000
6	0.061670000	-1.210223000	-0.044249000
6	-1.337240000	-1.306939000	-0.046770000
1	-2.107799000	2.027983000	0.072073000
1	-1.843742000	-2.272288000	-0.078721000
6	2.221543000	0.172181000	-0.157615000
8	2.728103000	0.668849000	-1.134321000
8	2.954210000	-0.298291000	0.874069000
1(iso=2)	2.355581000	-0.658192000	1.547084000
6	0.548061000	2.591883000	0.053926000
6	0.866133000	-2.493751000	-0.024445000
6	-2.083281000	-0.128188000	-0.015957000
7	-3.565460000	-0.220148000	-0.014739000
8	-4.053181000	-1.349880000	-0.057527000
8	-4.189153000	0.840430000	0.028672000
9	1.621932000	2.603576000	0.865939000

9	0.933458000	2.992493000	-1.162443000
9	-0.323339000	3.503758000	0.526684000
9	0.108636000	-3.563008000	-0.305410000
9	1.393735000	-2.690168000	1.210571000
9	1.881445000	-2.462377000	-0.898452000

E = –1296.253682 au   ZPVE = 0.124399522 au

***TS*-7c**

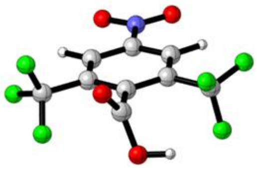

6	-1.511935000	-1.106188000	-0.031121000
6	-0.114118000	-1.212694000	-0.005163000
6	0.706620000	-0.063214000	0.027381000
6	0.080224000	1.203380000	0.046453000
6	-1.315982000	1.328947000	0.043497000
1	-2.149826000	-1.989727000	-0.076426000
1	-1.803586000	2.304193000	0.074279000
6	2.216059000	-0.207810000	0.115036000
8	2.755332000	-0.717981000	1.064529000
8	2.877544000	0.201938000	-1.017375000
1(iso=2)	3.054730000	1.153131000	-0.951678000
6	0.496199000	-2.602668000	-0.032785000
6	0.902353000	2.472172000	0.040390000
6	-2.085157000	0.165497000	0.004950000
7	-3.564828000	0.286842000	-0.005965000
8	-4.030572000	1.425871000	0.036017000
8	-4.209625000	-0.760665000	-0.055706000
9	1.564971000	-2.645552000	-0.848472000
9	0.885075000	-2.988524000	1.189840000
9	-0.392428000	-3.508832000	-0.481895000
9	0.199526000	3.532336000	0.454574000
9	1.359748000	2.745856000	-1.202733000
9	1.990381000	2.361012000	0.835800000

E = –1296.241082 au   ZPVE = 0.125393211 au ν_i = 418.36*i* cm^{-1}

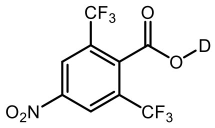

***Z*-7c**

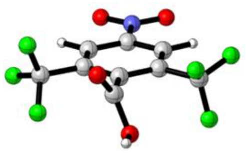

6	-1.456402000	1.180005000	0.026028000
6	-0.055408000	1.221145000	0.007312000
6	0.707521000	0.032566000	-0.014612000
6	0.031319000	-1.207184000	-0.032001000

6	-1.370298000	-1.263502000	-0.031292000
1	-2.053937000	2.091540000	0.061830000
1	-1.903044000	-2.214728000	-0.059305000
6	2.215095000	0.106180000	-0.088287000
8	2.824477000	0.579230000	-1.022093000
8	2.772028000	-0.418930000	1.020741000
1(iso=2)	3.735904000	-0.362653000	0.877605000
6	0.619041000	2.580690000	0.024237000
6	0.787790000	-2.522567000	-0.039318000
6	-2.085181000	-0.065791000	-0.000290000
7	-3.569284000	-0.118655000	0.008324000
8	-4.087947000	-1.234919000	-0.021328000
8	-4.165408000	0.958056000	0.044328000
9	1.678740000	2.588174000	0.855636000
9	1.043224000	2.931580000	-1.196812000
9	-0.230908000	3.534499000	0.448959000
9	0.015595000	-3.518629000	-0.511439000
9	1.176528000	-2.866937000	1.197862000
9	1.883490000	-2.459833000	-0.818899000

E = –1296.261004 au   ZPVE = 0.124576016 au

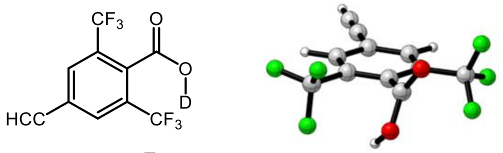

***E*-7e**

6	-1.282826000	1.610270000	0.028149000
6	-1.224262000	0.210585000	0.011040000
6	0.016751000	-0.466695000	-0.018732000
6	1.198158000	0.311159000	-0.047896000
6	1.145416000	1.711959000	-0.049797000
6	-0.098329000	2.375086000	-0.017221000
1	-2.249614000	2.116686000	0.073233000
1	2.068139000	2.295423000	-0.083736000
6	0.046102000	-1.981135000	-0.151884000
8	-0.404128000	-2.549622000	-1.117131000
8	0.601246000	-2.652646000	0.881692000
1(iso=2)	0.902238000	-2.011097000	1.543819000
6	2.556039000	-0.354842000	-0.031991000
6	-2.529497000	-0.560593000	0.054493000
6	-0.157680000	3.811432000	-0.017904000
6	-0.209363000	5.044673000	-0.019028000
1	-0.254859000	6.120377000	-0.020907000
9	-3.530406000	0.212534000	0.523810000
9	-2.892943000	-0.991883000	-1.159729000
9	-2.436193000	-1.628546000	0.870858000
9	3.543609000	0.506006000	-0.323157000

9 2.819004000 -0.854590000 1.204091000
9 2.629606000 -1.374039000 -0.900791000

E = –1168.153744 au ZPVE = 0.129923825 au

***TS*-7e**

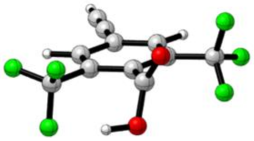

6 1.314876000 1.590484000 -0.033571000
6 1.230820000 0.191927000 -0.006548000
6 -0.020678000 -0.463566000 0.027366000
6 -1.186014000 0.336552000 0.047064000
6 -1.109980000 1.735884000 0.043533000
6 0.144926000 2.377007000 0.003025000
1 2.290895000 2.078520000 -0.081478000
1 -2.022487000 2.335688000 0.076569000
6 -0.087962000 -1.976657000 0.114709000
8 0.358395000 -2.587847000 1.053055000
8 -0.610500000 -2.575394000 -1.008842000
1(iso=2) -1.575068000 -2.615183000 -0.919793000
6 -2.556208000 -0.296657000 0.044215000
6 2.520903000 -0.603914000 -0.037481000
6 0.229610000 3.812259000 -0.006979000
6 0.302551000 5.044348000 -0.015159000
1 0.366965000 6.118994000 -0.022177000
9 2.857146000 -1.046482000 1.183018000
9 2.418257000 -1.668678000 -0.854959000
9 3.544679000 0.149252000 -0.487719000
9 -2.599751000 -1.397377000 0.832050000
9 -3.507487000 0.543999000 0.472078000
9 -2.906237000 -0.702725000 -1.198676000

E = –1168.140605 au ZPVE = 0.128616861 au ν_i = 416.70i cm^{-1}

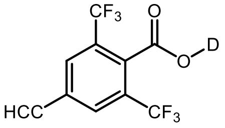

***Z*-7e**

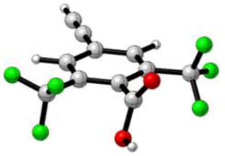

6 -1.257561000 1.637629000 0.028772000
6 -1.226788000 0.237090000 0.008315000
6 0.000850000 -0.461779000 -0.014901000
6 1.200008000 0.284737000 -0.032129000
6 1.173386000 1.686558000 -0.030043000
6 -0.056048000 2.375736000 0.002765000
1 -2.213712000 2.164189000 0.066232000
1 2.107618000 2.251772000 -0.059461000

6	0.010875000	-1.969523000	-0.086331000
8	-0.433506000	-2.610628000	-1.013438000
8	0.574602000	-2.497185000	1.019913000
1(iso=2)	0.571633000	-3.461908000	0.872433000
6	2.553266000	-0.396405000	-0.043045000
6	-2.545124000	-0.509964000	0.026079000
6	-0.084574000	3.813437000	0.011851000
6	-0.109415000	5.047493000	0.019489000
1	-0.131148000	6.123803000	0.025588000
9	-3.548352000	0.284056000	0.451494000
9	-2.878292000	-0.955382000	-1.193940000
9	-2.497789000	-1.569961000	0.857759000
9	3.507700000	0.427514000	-0.519359000
9	2.928650000	-0.766397000	1.192465000
9	2.552926000	-1.495986000	-0.821920000

E = –1168.160489 au   ZPVE = 0.130066853 au

2. Tunneling Computations

Computational tunneling half-lives were determined at the CVT/SCT//MP2/cc-pVDZ level of theory using Polyrate Version 2017-c.[3]

Table S2.1. Computed half-lives from reactions of *E* → *Z* isomerization for selected protium carboxylic acids (ArCOOH). (CVT/SCT//MP2/cc-pVDZ).

Compound	R=	X=	$t_{1/2(comp)}$ (min)
1a	H	H	2.0×10^{-6}
1f	H	CN	1.3×10^{-7}

2a	Me	H	4.9×10^{-2}
2e	Me	Cl	2.1×10^{-2}
2f	Me	CN	4.7×10^{-3}
4a	CF3	H	1.3×10^{-3}
4d	CF3	CN	7.5×10^{-4}

Table S2.2. Rate constants of the isomerization reaction ***E-1a*** → ***Z*-1a** in s^{-1} computed at the CVT/SCT//MP2/cc-pVDZ level of theory using polyrate.

T(K)	TST	CVT	CVT/ZCT	CVT/SCT
0.10	NaN	NaN	NaN	NaN
1.00	NaN	NaN	NaN	NaN
5.00	3.82-207	3.81-207	1.83E+02	5.86E+03
10.00	3.99E-98	3.99E-98	1.83E+02	5.86E+03
11.00	3.36E-88	3.36E-88	1.83E+02	5.86E+03
15.00	1.04E-61	1.04E-61	1.83E+02	5.86E+03
20.00	1.82E-43	1.82E-43	1.83E+02	5.85E+03
25.00	1.69E-32	1.69E-32	1.83E+02	5.83E+03
30.00	3.58E-25	3.58E-25	1.82E+02	5.81E+03
35.00	6.26E-20	6.26E-20	1.81E+02	5.79E+03
40.00	5.44E-16	5.44E-16	1.80E+02	5.76E+03
45.00	6.38E-13	6.38E-13	1.80E+02	5.74E+03
50.00	1.84E-10	1.84E-10	1.80E+02	5.72E+03
60.00	9.20E-07	9.20E-07	1.86E+02	5.76E+03
70.00	4.13E-04	4.13E-04	2.26E+02	6.15E+03
80.00	4.09E-02	4.09E-02	3.71E+02	7.56E+03
90.00	1.48E+00	1.48E+00	8.30E+02	1.14E+04
100.00	2.64E+01	2.63E+01	2.34E+03	2.11E+04
110.00	2.80E+02	2.80E+02	7.54E+03	4.53E+04
120.00	2.02E+03	2.02E+03	2.52E+04	1.07E+05
130.00	1.08E+04	1.08E+04	8.04E+04	2.63E+05
140.00	4.59E+04	4.59E+04	2.35E+05	6.39E+05
150.00	1.61E+05	1.61E+05	6.27E+05	1.49E+06
160.00	4.83E+05	4.83E+05	1.52E+06	3.26E+06
170.00	1.28E+06	1.28E+06	3.40E+06	6.74E+06
180.00	3.05E+06	3.05E+06	7.04E+06	1.31E+07
190.00	6.63E+06	6.63E+06	1.37E+07	2.42E+07
200.00	1.34E+07	1.34E+07	2.51E+07	4.24E+07
220.00	4.52E+07	4.52E+07	7.29E+07	1.15E+08
240.00	1.25E+08	1.25E+08	1.81E+08	2.68E+08
260.00	2.97E+08	2.97E+08	3.96E+08	5.59E+08
280.00	6.25E+08	6.25E+08	7.84E+08	1.06E+09
298.00	1.12E+09	1.12E+09	1.35E+09	1.77E+09

Table S2.3. Rate constants of the isomerization reaction ***E-1f*** → ***Z*-1f** in s^{-1} computed at the CVT/SCT//MP2/cc-pVDZ level of theory using polyrate.

T(K)	TST	CVT	CVT/ZCT	CVT/SCT
0.10	NaN	NaN	NaN	NaN
1.00	NaN	NaN	NaN	NaN
5.00	3.00-185	3.00-185	4.44E+03	8.95E+04
10.00	3.54E-87	3.54E-87	4.44E+03	8.95E+04
11.00	3.01E-78	3.01E-78	4.44E+03	8.95E+04

15.00	2.07E-54	2.07E-54	4.44E+03	8.94E+04
20.00	5.43E-38	5.43E-38	4.43E+03	8.93E+04
25.00	4.05E-28	4.05E-28	4.41E+03	8.89E+04
30.00	1.59E-21	1.59E-21	4.39E+03	8.85E+04
35.00	8.37E-17	8.37E-17	4.37E+03	8.80E+04
40.00	2.95E-13	2.95E-13	4.34E+03	8.75E+04
45.00	1.72E-10	1.72E-10	4.32E+03	8.71E+04
50.00	2.83E-08	2.83E-08	4.31E+03	8.67E+04
60.00	6.09E-05	6.09E-05	4.36E+03	8.67E+04
70.00	1.50E-02	1.50E-02	4.79E+03	8.99E+04
80.00	9.46E-01	9.46E-01	6.32E+03	1.02E+05
90.00	2.41E+01	2.41E+01	1.09E+04	1.33E+05
100.00	3.24E+02	3.24E+02	2.48E+04	2.08E+05
110.00	2.74E+03	2.74E+03	6.83E+04	3.82E+05
120.00	1.63E+04	1.63E+04	2.02E+05	7.86E+05
130.00	7.43E+04	7.43E+04	5.87E+05	1.71E+06
140.00	2.74E+05	2.74E+05	1.59E+06	3.75E+06
150.00	8.50E+05	8.50E+05	3.94E+06	8.01E+06
160.00	2.30E+06	2.30E+06	8.97E+06	1.64E+07
170.00	5.54E+06	5.54E+06	1.89E+07	3.18E+07
180.00	1.22E+07	1.21E+07	3.70E+07	5.88E+07
190.00	2.46E+07	2.46E+07	6.80E+07	1.03E+08
200.00	4.64E+07	4.64E+07	1.18E+08	1.73E+08
220.00	1.39E+08	1.39E+08	3.13E+08	4.33E+08
240.00	3.51E+08	3.51E+08	7.09E+08	9.46E+08
260.00	7.67E+08	7.67E+08	1.43E+09	1.85E+09
280.00	1.51E+09	1.51E+09	2.62E+09	3.32E+09
298.00	2.56E+09	2.56E+09	4.24E+09	5.26E+09

Table S2.4. Rate constants of the isomerization reaction ***E-2a*** → ***Z*-2a** in s^{-1} computed at the CVT/SCT//MP2/cc-pVDZ level of theory using polyrate.

T(K)	TST	CVT	CVT/ZCT	CVT/SCT
0.10	NaN	NaN	NaN	NaN
1.00	NaN	NaN	NaN	NaN
5.00	0.00E+00	0.00E+00	NaN	NaN
10.00	4.13-156	2.47-156	2.32E-03	2.33E-01
11.00	6.49-141	3.89-141	2.35E-03	2.36E-01
15.00	2.27-100	1.38-100	2.45E-03	2.46E-01
20.00	1.80E-72	1.11E-72	2.50E-03	2.52E-01
25.00	1.02E-55	6.40E-56	2.50E-03	2.51E-01
30.00	1.52E-44	9.71E-45	2.46E-03	2.47E-01
35.00	1.47E-36	9.50E-37	2.40E-03	2.41E-01
40.00	1.44E-30	9.39E-31	2.32E-03	2.33E-01
45.00	6.55E-26	4.33E-26	2.23E-03	2.25E-01
50.00	3.49E-22	2.33E-22	2.15E-03	2.16E-01
60.00	1.35E-16	9.16E-17	2.01E-03	1.99E-01
70.00	1.32E-12	9.05E-13	2.11E-03	1.89E-01
80.00	1.28E-09	8.92E-10	2.89E-03	1.98E-01
90.00	2.69E-07	1.89E-07	5.65E-03	2.51E-01
100.00	1.93E-05	1.37E-05	1.52E-02	4.02E-01
110.00	6.34E-04	4.54E-04	5.47E-02	8.19E-01
120.00	1.16E-02	8.37E-03	2.38E-01	2.07E+00
130.00	1.35E-01	9.83E-02	1.09E+00	6.10E+00
140.00	1.11E+00	8.12E-01	4.71E+00	1.93E+01
150.00	6.86E+00	5.05E+00	1.84E+01	6.02E+01
160.00	3.37E+01	2.49E+01	6.44E+01	1.78E+02
170.00	1.37E+02	1.02E+02	2.03E+02	4.90E+02
180.00	4.77E+02	3.56E+02	5.85E+02	1.25E+03
190.00	1.45E+03	1.09E+03	1.54E+03	2.99E+03

200.00	3.95E+03	2.98E+03	3.77E+03	6.69E+03
220.00	2.22E+04	1.69E+04	1.83E+04	2.82E+04
240.00	9.32E+04	7.13E+04	7.09E+04	9.76E+04
260.00	3.13E+05	2.41E+05	2.27E+05	2.88E+05
280.00	8.80E+05	6.82E+05	6.21E+05	7.45E+05
298.00	1.98E+06	1.54E+06	1.38E+06	1.59E+06

Table S2.5. Rate constants of the isomerization reaction ***E*-2*e*** → ***Z*-2e** in s^{-1} computed at the CVT/SCT//MP2/cc-pVDZ level of theory using polyrate.

T(K)	TST	CVT	CVT/ZCT	CVT/SCT
0.10	NaN	NaN	NaN	NaN
1.00	NaN	NaN	NaN	NaN
5.00	0.00E+00	0.00E+00	NaN	NaN
10.00	1.07-149	7.07-150	4.34E-03	5.30E-01
11.00	4.41-135	2.90-135	4.38E-03	5.36E-01
15.00	4.31E-96	2.85E-96	4.52E-03	5.53E-01
20.00	2.92E-69	1.95E-69	4.58E-03	5.61E-01
25.00	3.78E-53	2.56E-53	4.56E-03	5.57E-01
30.00	2.12E-42	1.45E-42	4.47E-03	5.47E-01
35.00	1.01E-34	6.98E-35	4.34E-03	5.31E-01
40.00	5.85E-29	4.07E-29	4.19E-03	5.13E-01
45.00	1.77E-24	1.24E-24	4.03E-03	4.93E-01
50.00	6.81E-21	4.80E-21	3.88E-03	4.72E-01
60.00	1.61E-15	1.15E-15	3.72E-03	4.35E-01
70.00	1.11E-11	7.97E-12	4.37E-03	4.20E-01
80.00	8.27E-09	6.01E-09	7.52E-03	4.66E-01
90.00	1.41E-06	1.04E-06	1.78E-02	6.47E-01
100.00	8.60E-05	6.35E-05	5.21E-02	1.14E+00
110.00	2.47E-03	1.83E-03	1.90E-01	2.45E+00
120.00	4.04E-02	3.02E-02	8.22E-01	6.29E+00
130.00	4.29E-01	3.22E-01	3.71E+00	1.85E+01
140.00	3.24E+00	2.44E+00	1.58E+01	5.76E+01
150.00	1.86E+01	1.41E+01	6.01E+01	1.77E+02
160.00	8.61E+01	6.55E+01	2.04E+02	5.18E+02
170.00	3.31E+02	2.53E+02	6.21E+02	1.40E+03
180.00	1.10E+03	8.41E+02	1.71E+03	3.52E+03
190.00	3.20E+03	2.46E+03	4.32E+03	8.21E+03
200.00	8.36E+03	6.46E+03	1.01E+04	1.79E+04
220.00	4.39E+04	3.41E+04	4.47E+04	7.10E+04
240.00	1.74E+05	1.36E+05	1.59E+05	2.31E+05
260.00	5.56E+05	4.37E+05	4.74E+05	6.43E+05
280.00	1.50E+06	1.19E+06	1.22E+06	1.57E+06
298.00	3.27E+06	2.59E+06	2.58E+06	3.18E+06

Table S2.6. Rate constants of the isomerization reaction ***E*-2*f*** → ***Z*-2f** in s^{-1} computed at the CVT/SCT//MP2/cc-pVDZ level of theory using polyrate.

T(K)	TST	CVT	CVT/ZCT	CVT/SCT
0.10	NaN	NaN	NaN	NaN
1.00	NaN	NaN	NaN	NaN
5.00	0.00E+00	0.00E+00	NaN	NaN
10.00	7.22-148	1.32-148	4.13E-02	2.40E+00
11.00	2.02-133	4.25-134	4.22E-02	2.45E+00
15.00	7.05E-95	2.16E-95	4.42E-02	2.57E+00
20.00	2.34E-68	9.17E-69	4.47E-02	2.60E+00

25.00	1.96E-52	8.88E-53	4.41E-02	2.56E+00
30.00	8.20E-42	4.08E-42	4.27E-02	2.48E+00
35.00	3.18E-34	1.69E-34	4.11E-02	2.39E+00
40.00	1.56E-28	8.74E-29	3.93E-02	2.28E+00
45.00	4.18E-24	2.43E-24	3.74E-02	2.17E+00
50.00	1.45E-20	8.69E-21	3.55E-02	2.06E+00
60.00	2.95E-15	1.85E-15	3.23E-02	1.86E+00
70.00	1.81E-11	1.18E-11	3.07E-02	1.72E+00
80.00	1.25E-08	8.31E-09	3.32E-02	1.67E+00
90.00	2.00E-06	1.36E-06	4.63E-02	1.84E+00
100.00	1.16E-04	7.99E-05	9.41E-02	2.44E+00
110.00	3.18E-03	2.23E-03	2.90E-01	4.15E+00
120.00	5.03E-02	3.56E-02	1.17E+00	9.27E+00
130.00	5.18E-01	3.71E-01	4.98E+00	2.54E+01
140.00	3.81E+00	2.76E+00	1.98E+01	7.58E+01
150.00	2.15E+01	1.56E+01	7.03E+01	2.24E+02
160.00	9.73E+01	7.14E+01	2.22E+02	6.24E+02
170.00	3.68E+02	2.72E+02	6.33E+02	1.61E+03
180.00	1.20E+03	8.92E+02	1.64E+03	3.85E+03
190.00	3.46E+03	2.58E+03	3.93E+03	8.57E+03
200.00	8.93E+03	6.70E+03	8.76E+03	1.79E+04
220.00	4.59E+04	3.47E+04	3.64E+04	6.58E+04
240.00	1.79E+05	1.36E+05	1.24E+05	2.02E+05
260.00	5.64E+05	4.33E+05	3.59E+05	5.36E+05
280.00	1.50E+06	1.16E+06	9.08E+05	1.26E+06
298.00	3.24E+06	2.51E+06	1.90E+06	2.50E+06

Table S2.7. Rate constants of the isomerization reaction ***E*-*4a*** ⟶ ***Z*-4a** in s^{-1} computed at the CVT/SCT//MP2/cc-pVDZ level of theory using polyrate.

T(K)	TST	CVT	CVT/ZCT	CVT/SCT
0.10	NaN	NaN	NaN	NaN
1.00	NaN	NaN	NaN	NaN
5.00	1.56-295	1.55-295	1.81E-01	8.81E+00
10.00	2.55-142	2.52-142	1.81E-01	8.81E+00
11.00	2.23-128	2.21-128	1.81E-01	8.82E+00
15.00	3.59E-91	3.55E-91	1.82E-01	8.85E+00
20.00	1.47E-65	1.45E-65	1.83E-01	8.91E+00
25.00	3.62E-50	3.58E-50	1.84E-01	8.97E+00
30.00	6.81E-40	6.74E-40	1.85E-01	9.01E+00
35.00	1.52E-32	1.50E-32	1.85E-01	9.01E+00
40.00	5.00E-27	4.95E-27	1.84E-01	8.98E+00
45.00	9.89E-23	9.79E-23	1.83E-01	8.93E+00
50.00	2.72E-19	2.69E-19	1.82E-01	8.85E+00
60.00	3.98E-14	3.95E-14	1.80E-01	8.69E+00
70.00	1.97E-10	1.95E-10	1.86E-01	8.66E+00
80.00	1.17E-07	1.16E-07	2.21E-01	9.12E+00
90.00	1.69E-05	1.68E-05	3.36E-01	1.07E+01
100.00	9.05E-04	8.97E-04	7.20E-01	1.50E+01
110.00	2.35E-02	2.33E-02	2.20E+00	2.65E+01
120.00	3.56E-01	3.53E-01	8.48E+00	5.91E+01
130.00	3.54E+00	3.51E+00	3.49E+01	1.58E+02
140.00	2.54E+01	2.52E+01	1.37E+02	4.59E+02
150.00	1.40E+02	1.39E+02	4.90E+02	1.33E+03
160.00	6.24E+02	6.20E+02	1.59E+03	3.69E+03
170.00	2.33E+03	2.31E+03	4.67E+03	9.61E+03
180.00	7.52E+03	7.46E+03	1.26E+04	2.34E+04
190.00	2.14E+04	2.13E+04	3.11E+04	5.32E+04
200.00	5.49E+04	5.45E+04	7.17E+04	1.14E+05

220.00	2.79E+05	2.77E+05	3.14E+05	4.45E+05
240.00	1.08E+06	1.07E+06	1.11E+06	1.44E+06
260.00	3.37E+06	3.34E+06	3.27E+06	4.00E+06
280.00	8.93E+06	8.87E+06	8.37E+06	9.78E+06
298.00	1.92E+07	1.90E+07	1.76E+07	1.99E+07

Table S2.8. Rate constants of the isomerization reaction ***E-4d*** → ***Z*-4d** in s^{-1} computed at the CVT/SCT//MP2/cc-pVDZ level of theory using polyrate.

T(K)	TST	CVT	CVT/ZCT	CVT/SCT
0.10	NaN	NaN	NaN	NaN
1.00	NaN	NaN	NaN	NaN
5.00	1.27-287	1.26-287	2.99E-01	1.54E+01
10.00	2.30-138	2.30-138	2.99E-01	1.54E+01
11.00	8.81-125	8.80-125	2.99E-01	1.54E+01
15.00	1.55E-88	1.55E-88	2.99E-01	1.54E+01
20.00	1.40E-63	1.40E-63	3.01E-01	1.55E+01
25.00	1.38E-48	1.38E-48	3.02E-01	1.56E+01
30.00	1.42E-38	1.42E-38	3.03E-01	1.56E+01
35.00	2.05E-31	2.05E-31	3.03E-01	1.56E+01
40.00	4.86E-26	4.86E-26	3.02E-01	1.55E+01
45.00	7.46E-22	7.46E-22	3.00E-01	1.54E+01
50.00	1.68E-18	1.68E-18	2.97E-01	1.53E+01
60.00	1.81E-13	1.81E-13	2.93E-01	1.50E+01
70.00	7.22E-10	7.21E-10	3.02E-01	1.49E+01
80.00	3.64E-07	3.64E-07	3.54E-01	1.57E+01
90.00	4.63E-05	4.63E-05	5.54E-01	1.85E+01
100.00	2.24E-03	2.24E-03	1.35E+00	2.68E+01
110.00	5.36E-02	5.36E-02	4.87E+00	5.19E+01
120.00	7.57E-01	7.56E-01	2.07E+01	1.31E+02
130.00	7.11E+00	7.11E+00	8.69E+01	3.85E+02
140.00	4.85E+01	4.85E+01	3.34E+02	1.16E+03
150.00	2.56E+02	2.56E+02	1.15E+03	3.38E+03
160.00	1.10E+03	1.10E+03	3.56E+03	9.17E+03
170.00	3.96E+03	3.96E+03	9.98E+03	2.31E+04
180.00	1.24E+04	1.24E+04	2.56E+04	5.41E+04
190.00	3.44E+04	3.44E+04	6.06E+04	1.18E+05
200.00	8.61E+04	8.61E+04	1.34E+05	2.43E+05
220.00	4.19E+05	4.19E+05	5.43E+05	8.76E+05
240.00	1.56E+06	1.56E+06	1.80E+06	2.64E+06
260.00	4.75E+06	4.75E+06	5.06E+06	6.88E+06
280.00	1.23E+07	1.23E+07	1.24E+07	1.59E+07
298.00	2.58E+07	2.58E+07	2.53E+07	3.11E+07

Table S2.9. Computed half-lives from reactions of *E* → *Z* isomerization for selected deuterated carboxylic acids (ArCOOD). (CVT/SCT//MP2/cc-pVDZ).

Compound	R=	X=	$t_{1/2(comp)}$ (min)
5a	H	H	0.036
5c	H	Me	0.047
5d	H	Cl	0.0056
6a	Me	H	38100
6b	Me	F	53000
6c	Me	Me	17600
6e	Me	Cl	18000

7a	CF3	H	1100

Table S2.10. Rate constants of the isomerization reaction ***E-5a*** → ***Z*-5a** in s^{-1} computed at the CVT/SCT//MP2/cc-pVDZ level of theory using polyrate.

T(K)	TST	CVT	CVT/ZCT	CVT/SCT
0.10	NaN	NaN	NaN	NaN
1.00	NaN	NaN	NaN	NaN
5.00	1.88-217	1.88-217	7.18E-03	3.20E-01
10.00	2.81-103	2.81-103	7.18E-03	3.20E-01
11.00	6.95E-93	6.95E-93	7.18E-03	3.20E-01
15.00	3.82E-65	3.82E-65	7.18E-03	3.20E-01
20.00	4.85E-46	4.85E-46	7.17E-03	3.19E-01
25.00	1.47E-34	1.47E-34	7.15E-03	3.18E-01
30.00	6.88E-27	6.88E-27	7.12E-03	3.17E-01
35.00	2.11E-21	2.11E-21	7.11E-03	3.16E-01
40.00	2.81E-17	2.81E-17	7.16E-03	3.16E-01
45.00	4.58E-14	4.58E-14	7.45E-03	3.21E-01
50.00	1.72E-11	1.72E-11	8.36E-03	3.35E-01
60.00	1.28E-07	1.28E-07	1.61E-02	4.54E-01
70.00	7.60E-05	7.60E-05	6.32E-02	9.66E-01
80.00	9.30E-03	9.30E-03	4.90E-01	3.47E+00
90.00	3.96E-01	3.96E-01	5.49E+00	1.93E+01
100.00	8.03E+00	8.03E+00	5.81E+01	1.29E+02
110.00	9.49E+01	9.49E+01	4.80E+02	8.22E+02
120.00	7.48E+02	7.48E+02	3.02E+03	4.44E+03
130.00	4.30E+03	4.30E+03	1.49E+04	1.99E+04
140.00	1.94E+04	1.94E+04	5.95E+04	7.50E+04
150.00	7.15E+04	7.15E+04	2.00E+05	2.42E+05
160.00	2.25E+05	2.25E+05	5.83E+05	6.86E+05
170.00	6.19E+05	6.19E+05	1.51E+06	1.73E+06
180.00	1.53E+06	1.53E+06	3.51E+06	3.98E+06
190.00	3.42E+06	3.42E+06	7.51E+06	8.40E+06
200.00	7.09E+06	7.09E+06	1.49E+07	1.65E+07
220.00	2.50E+07	2.50E+07	4.90E+07	5.34E+07
240.00	7.19E+07	7.19E+07	1.33E+08	1.43E+08
260.00	1.76E+08	1.76E+08	3.08E+08	3.29E+08
280.00	3.79E+08	3.79E+08	6.38E+08	6.76E+08
298.00	6.94E+08	6.94E+08	1.13E+09	1.19E+09

Table S2.11. Rate constants of the isomerization reaction ***E-5c*** → ***Z*-5c** in s^{-1} computed at the CVT/SCT//MP2/cc-pVDZ level of theory using polyrate.

T(K)	TST	CVT	CVT/ZCT	CVT/SCT
0.10	NaN	NaN	NaN	NaN
1.00	NaN	NaN	NaN	NaN
5.00	1.00-218	1.00-218	5.40E-03	2.43E-01
10.00	6.51-104	6.51-104	5.43E-03	2.44E-01
11.00	1.85E-93	1.84E-93	5.44E-03	2.45E-01
15.00	1.46E-65	1.46E-65	5.49E-03	2.47E-01
20.00	2.40E-46	2.40E-46	5.56E-03	2.50E-01
25.00	8.56E-35	8.55E-35	5.61E-03	2.53E-01
30.00	4.45E-27	4.45E-27	5.66E-03	2.55E-01
35.00	1.48E-21	1.48E-21	5.69E-03	2.56E-01
40.00	2.09E-17	2.09E-17	5.78E-03	2.58E-01

45.00	3.57E-14	3.57E-14	6.06E-03	2.63E-01
50.00	1.39E-11	1.39E-11	6.87E-03	2.77E-01
60.00	1.09E-07	1.09E-07	1.38E-02	3.86E-01
70.00	6.79E-05	6.79E-05	5.59E-02	8.58E-01
80.00	8.58E-03	8.58E-03	4.16E-01	3.15E+00
90.00	3.74E-01	3.74E-01	3.96E+00	1.69E+01
100.00	7.75E+00	7.75E+00	3.40E+01	1.02E+02
110.00	9.31E+01	9.31E+01	2.35E+02	5.58E+02
120.00	7.44E+02	7.44E+02	1.32E+03	2.62E+03
130.00	4.33E+03	4.33E+03	6.06E+03	1.05E+04
140.00	1.97E+04	1.97E+04	2.36E+04	3.65E+04
150.00	7.34E+04	7.34E+04	7.92E+04	1.12E+05
160.00	2.32E+05	2.32E+05	2.34E+05	3.08E+05
170.00	6.44E+05	6.44E+05	6.19E+05	7.68E+05
180.00	1.60E+06	1.60E+06	1.48E+06	1.76E+06
190.00	3.61E+06	3.61E+06	3.27E+06	3.76E+06
200.00	7.51E+06	7.51E+06	6.71E+06	7.50E+06
220.00	2.67E+07	2.67E+07	2.35E+07	2.52E+07
240.00	7.73E+07	7.73E+07	6.74E+07	7.05E+07
260.00	1.90E+08	1.90E+08	1.65E+08	1.70E+08
280.00	4.13E+08	4.13E+08	3.59E+08	3.65E+08
298.00	7.59E+08	7.59E+08	6.62E+08	6.68E+08

Table S2.12. Rate constants of the isomerization reaction ***E-5d*** → ***Z*-5d** in s^{-1} computed at the CVT/SCT//MP2/cc-pVDZ level of theory using polyrate.

T(K)	TST	CVT	CVT/ZCT	CVT/SCT
0.10	NaN	NaN	NaN	NaN
1.00	NaN	NaN	NaN	NaN
5.00	2.96-202	2.96-202	5.61E-02	2.05E+00
10.00	1.11E-95	1.11E-95	5.61E-02	2.05E+00
11.00	5.62E-86	5.61E-86	5.61E-02	2.05E+00
15.00	4.44E-60	4.44E-60	5.61E-02	2.05E+00
20.00	3.05E-42	3.05E-42	5.60E-02	2.05E+00
25.00	1.61E-31	1.61E-31	5.58E-02	2.04E+00
30.00	2.34E-24	2.34E-24	5.55E-02	2.03E+00
35.00	3.12E-19	3.12E-19	5.53E-02	2.02E+00
40.00	2.21E-15	2.21E-15	5.56E-02	2.02E+00
45.00	2.22E-12	2.22E-12	5.78E-02	2.04E+00
50.00	5.63E-10	5.63E-10	6.47E-02	2.14E+00
60.00	2.33E-06	2.33E-06	1.25E-01	2.91E+00
70.00	9.13E-04	9.13E-04	4.83E-01	6.24E+00
80.00	8.17E-02	8.17E-02	3.39E+00	2.18E+01
90.00	2.72E+00	2.72E+00	3.02E+01	1.10E+02
100.00	4.54E+01	4.54E+01	2.40E+02	6.22E+02
110.00	4.58E+02	4.58E+02	1.51E+03	3.21E+03
120.00	3.16E+03	3.15E+03	7.56E+03	1.41E+04
130.00	1.62E+04	1.62E+04	3.10E+04	5.23E+04
140.00	6.63E+04	6.63E+04	1.07E+05	1.68E+05
150.00	2.25E+05	2.25E+05	3.21E+05	4.73E+05
160.00	6.58E+05	6.58E+05	8.55E+05	1.19E+06
170.00	1.70E+06	1.70E+06	2.06E+06	2.74E+06
180.00	3.95E+06	3.95E+06	4.53E+06	5.82E+06
190.00	8.41E+06	8.41E+06	9.24E+06	1.15E+07
200.00	1.66E+07	1.66E+07	1.77E+07	2.14E+07
220.00	5.42E+07	5.42E+07	5.49E+07	6.38E+07
240.00	1.46E+08	1.46E+08	1.43E+08	1.61E+08
260.00	3.36E+08	3.36E+08	3.24E+08	3.56E+08
280.00	6.91E+08	6.91E+08	6.57E+08	7.09E+08

298.00	1.22E+09	1.22E+09	1.15E+09	1.22E+09

Table S2.13. Rate constants of the isomerization reaction ***E-6a*** → ***Z*-6a** in s^{-1} computed at the CVT/SCT//MP2/cc-pVDZ level of theory using polyrate.

T(K)	TST	CVT	CVT/ZCT	CVT/SCT
0.10	NaN	NaN	NaN	NaN
1.00	NaN	NaN	NaN	NaN
5.00	0.00E+00	0.00E+00	NaN	NaN
10.00	8.81-162	6.54-162	2.14E-09	3.01E-07
11.00	4.54-146	3.38-146	2.15E-09	3.03E-07
15.00	3.77-104	2.84-104	2.19E-09	3.09E-07
20.00	2.63E-75	2.01E-75	2.19E-09	3.08E-07
25.00	5.48E-58	4.25E-58	2.14E-09	3.02E-07
30.00	1.95E-46	1.53E-46	2.08E-09	2.93E-07
35.00	3.51E-38	2.77E-38	2.01E-09	2.83E-07
40.00	5.46E-32	4.35E-32	1.93E-09	2.71E-07
45.00	3.57E-27	2.86E-27	1.89E-09	2.62E-07
50.00	2.54E-23	2.05E-23	1.95E-09	2.58E-07
60.00	1.52E-17	1.24E-17	2.98E-09	2.97E-07
70.00	2.02E-13	1.66E-13	1.01E-08	5.42E-07
80.00	2.48E-10	2.05E-10	8.80E-08	1.87E-06
90.00	6.22E-08	5.17E-08	1.81E-06	1.30E-05
100.00	5.14E-06	4.30E-06	4.87E-05	1.55E-04
110.00	1.90E-04	1.60E-04	1.05E-03	2.12E-03
120.00	3.82E-03	3.23E-03	1.56E-02	2.47E-02
130.00	4.83E-02	4.10E-02	1.63E-01	2.25E-01
140.00	4.24E-01	3.61E-01	1.25E+00	1.59E+00
150.00	2.78E+00	2.37E+00	7.44E+00	8.91E+00
160.00	1.44E+01	1.23E+01	3.55E+01	4.10E+01
170.00	6.10E+01	5.24E+01	1.41E+02	1.59E+02
180.00	2.20E+02	1.90E+02	4.84E+02	5.34E+02
190.00	6.94E+02	5.99E+02	1.45E+03	1.58E+03
200.00	1.94E+03	1.68E+03	3.91E+03	4.20E+03
220.00	1.15E+04	9.99E+03	2.15E+04	2.28E+04
240.00	5.02E+04	4.38E+04	8.90E+04	9.31E+04
260.00	1.74E+05	1.53E+05	2.94E+05	3.05E+05
280.00	5.04E+05	4.43E+05	8.18E+05	8.44E+05
298.00	1.16E+06	1.02E+06	1.82E+06	1.87E+06

Table S2.14. Rate constants of the isomerization reaction ***E-6b*** → ***Z*-6b** in s^{-1} computed at the CVT/SCT//MP2/cc-pVDZ level of theory using polyrate.

T(K)	TST	CVT	CVT/ZCT	CVT/SCT
0.10	NaN	NaN	NaN	NaN
1.00	NaN	NaN	NaN	NaN
5.00	0.00E+00	0.00E+00	NaN	NaN
10.00	1.29-155	7.27-156	4.73E-09	6.30E-07
11.00	1.83-140	1.04-140	4.80E-09	6.39E-07
15.00	4.88-100	2.84-100	4.98E-09	6.63E-07
20.00	3.22E-72	1.92E-72	5.08E-09	6.77E-07
25.00	1.63E-55	9.93E-56	5.08E-09	6.76E-07
30.00	2.27E-44	1.41E-44	5.00E-09	6.66E-07
35.00	2.09E-36	1.31E-36	4.88E-09	6.50E-07
40.00	1.96E-30	1.25E-30	4.77E-09	6.31E-07

45.00	8.70E-26	5.60E-26	4.78E-09	6.18E-07
50.00	4.53E-22	2.95E-22	5.15E-09	6.22E-07
60.00	1.70E-16	1.12E-16	9.59E-09	7.93E-07
70.00	1.61E-12	1.08E-12	3.84E-08	1.69E-06
80.00	1.54E-09	1.04E-09	3.47E-07	6.44E-06
90.00	3.17E-07	2.17E-07	6.48E-06	4.50E-05
100.00	2.24E-05	1.55E-05	1.39E-04	4.93E-04
110.00	7.27E-04	5.08E-04	2.29E-03	5.70E-03
120.00	1.31E-02	9.25E-03	2.69E-02	5.48E-02
130.00	1.52E-01	1.08E-01	2.30E-01	4.12E-01
140.00	1.23E+00	8.78E-01	1.52E+00	2.46E+00
150.00	7.54E+00	5.41E+00	7.98E+00	1.20E+01
160.00	3.67E+01	2.65E+01	3.48E+01	4.91E+01
170.00	1.48E+02	1.07E+02	1.29E+02	1.73E+02
180.00	5.10E+02	3.71E+02	4.20E+02	5.39E+02
190.00	1.54E+03	1.13E+03	1.21E+03	1.50E+03
200.00	4.15E+03	3.05E+03	3.16E+03	3.80E+03
220.00	2.30E+04	1.70E+04	1.67E+04	1.92E+04
240.00	9.51E+04	7.08E+04	6.75E+04	7.50E+04
260.00	3.15E+05	2.36E+05	2.21E+05	2.39E+05
280.00	8.75E+05	6.60E+05	6.09E+05	6.49E+05
298.00	1.95E+06	1.48E+06	1.35E+06	1.42E+06

Table S2.15. Rate constants of the isomerization reaction ***E-6c*** ⟶ ***Z*-6c** in s^{-1} computed at the CVT/SCT//MP2/cc-pVDZ level of theory using polyrate.

T(K)	TST	CVT	CVT/ZCT	CVT/SCT
0.10	NaN	NaN	NaN	NaN
1.00	NaN	NaN	NaN	NaN
5.00	0.00E+00	0.00E+00	NaN	NaN
10.00	4.38-161	2.26-161	1.47E-09	2.15E-07
11.00	1.95-145	1.01-145	1.49E-09	2.18E-07
15.00	1.09-103	5.82-104	1.54E-09	2.25E-07
20.00	5.77E-75	3.18E-75	1.56E-09	2.28E-07
25.00	1.02E-57	5.75E-58	1.55E-09	2.26E-07
30.00	3.25E-46	1.87E-46	1.51E-09	2.21E-07
35.00	5.39E-38	3.16E-38	1.47E-09	2.14E-07
40.00	7.89E-32	4.69E-32	1.42E-09	2.07E-07
45.00	4.93E-27	2.97E-27	1.40E-09	2.01E-07
50.00	3.38E-23	2.06E-23	1.46E-09	1.99E-07
60.00	1.91E-17	1.19E-17	2.35E-09	2.34E-07
70.00	2.43E-13	1.54E-13	8.41E-09	4.41E-07
80.00	2.90E-10	1.85E-10	7.51E-08	1.58E-06
90.00	7.11E-08	4.60E-08	1.43E-06	1.10E-05
100.00	5.77E-06	3.77E-06	3.14E-05	1.23E-04
110.00	2.09E-04	1.38E-04	5.36E-04	1.46E-03
120.00	4.16E-03	2.77E-03	6.60E-03	1.44E-02
130.00	5.21E-02	3.50E-02	6.03E-02	1.12E-01
140.00	4.52E-01	3.06E-01	4.25E-01	6.96E-01
150.00	2.94E+00	2.00E+00	2.40E+00	3.56E+00
160.00	1.51E+01	1.03E+01	1.12E+01	1.53E+01
170.00	6.36E+01	4.38E+01	4.44E+01	5.71E+01
180.00	2.28E+02	1.58E+02	1.53E+02	1.87E+02
190.00	7.15E+02	4.98E+02	4.65E+02	5.46E+02
200.00	1.99E+03	1.39E+03	1.27E+03	1.45E+03
220.00	1.17E+04	8.24E+03	7.31E+03	7.96E+03
240.00	5.07E+04	3.60E+04	3.16E+04	3.34E+04
260.00	1.75E+05	1.25E+05	1.09E+05	1.13E+05
280.00	5.03E+05	3.63E+05	3.15E+05	3.23E+05

298.00	1.15E+06	8.34E+05	7.25E+05	7.36E+05

Table S2.16. Rate constants of the isomerization reaction ***E*-6e** ⟶ ***Z*-6e** in s^{-1} computed at the CVT/SCT//MP2/cc-pVDZ level of theory using polyrate.

T(K)	TST	CVT	CVT/ZCT	CVT/SCT
0.10	NaN	NaN	NaN	NaN
1.00	NaN	NaN	NaN	NaN
5.00	0.00E+00	0.00E+00	NaN	NaN
10.00	2.41-155	1.29-155	5.42E-09	6.47E-07
11.00	3.23-140	1.74-140	5.49E-09	6.56E-07
15.00	7.39-100	4.08-100	5.71E-09	6.83E-07
20.00	4.37E-72	2.49E-72	5.82E-09	6.96E-07
25.00	2.08E-55	1.21E-55	5.81E-09	6.94E-07
30.00	2.76E-44	1.64E-44	5.71E-09	6.82E-07
35.00	2.45E-36	1.48E-36	5.58E-09	6.65E-07
40.00	2.25E-30	1.37E-30	5.51E-09	6.49E-07
45.00	9.78E-26	6.04E-26	5.65E-09	6.45E-07
50.00	5.01E-22	3.13E-22	6.27E-09	6.67E-07
60.00	1.83E-16	1.16E-16	1.16E-08	8.99E-07
70.00	1.71E-12	1.10E-12	4.32E-08	1.90E-06
80.00	1.61E-09	1.05E-09	3.71E-07	6.95E-06
90.00	3.28E-07	2.17E-07	6.90E-06	4.70E-05
100.00	2.30E-05	1.54E-05	1.58E-04	5.16E-04
110.00	7.42E-04	5.00E-04	2.82E-03	6.24E-03
120.00	1.34E-02	9.07E-03	3.53E-02	6.38E-02
130.00	1.53E-01	1.05E-01	3.17E-01	5.08E-01
140.00	1.24E+00	8.54E-01	2.14E+00	3.17E+00
150.00	7.56E+00	5.24E+00	1.13E+01	1.60E+01
160.00	3.67E+01	2.56E+01	4.94E+01	6.67E+01
170.00	1.48E+02	1.03E+02	1.82E+02	2.38E+02
180.00	5.08E+02	3.58E+02	5.85E+02	7.43E+02
190.00	1.53E+03	1.08E+03	1.66E+03	2.07E+03
200.00	4.12E+03	2.93E+03	4.27E+03	5.21E+03
220.00	2.27E+04	1.63E+04	2.19E+04	2.59E+04
240.00	9.38E+04	6.78E+04	8.57E+04	9.89E+04
260.00	3.10E+05	2.25E+05	2.73E+05	3.08E+05
280.00	8.61E+05	6.30E+05	7.35E+05	8.19E+05
298.00	1.91E+06	1.41E+06	1.60E+06	1.76E+06

Table S2.17. Rate constants of the isomerization reaction ***E*-7a** ⟶ ***Z*-7a** in s^{-1} computed at the CVT/SCT//MP2/cc-pVDZ level of theory using polyrate.

T(K)	TST	CVT	CVT/ZCT	CVT/SCT
0.10	NaN	NaN	NaN	NaN
1.00	NaN	NaN	NaN	NaN
5.00	0.00E+00	0.00E+00	NaN	NaN
10.00	1.50-147	9.63-148	1.31E-07	1.04E-05
11.00	3.92-133	2.54-133	1.32E-07	1.05E-05
15.00	1.17E-94	7.73E-95	1.37E-07	1.09E-05
20.00	3.56E-68	2.41E-68	1.42E-07	1.12E-05
25.00	2.92E-52	2.01E-52	1.45E-07	1.15E-05
30.00	1.23E-41	8.55E-42	1.48E-07	1.18E-05
35.00	4.85E-34	3.42E-34	1.50E-07	1.19E-05
40.00	2.45E-28	1.75E-28	1.52E-07	1.20E-05
45.00	6.77E-24	4.86E-24	1.56E-07	1.22E-05

50.00	2.43E-20	1.76E-20	1.67E-07	1.26E-05
60.00	5.31E-15	3.90E-15	2.52E-07	1.54E-05
70.00	3.49E-11	2.59E-11	7.57E-07	2.76E-05
80.00	2.57E-08	1.92E-08	5.66E-06	8.78E-05
90.00	4.37E-06	3.30E-06	9.62E-05	5.45E-04
100.00	2.67E-04	2.03E-04	2.08E-03	5.65E-03
110.00	7.70E-03	5.90E-03	3.60E-02	6.60E-02
120.00	1.27E-01	9.79E-02	4.44E-01	6.62E-01
130.00	1.36E+00	1.05E+00	3.94E+00	5.24E+00
140.00	1.04E+01	8.08E+00	2.63E+01	3.26E+01
150.00	6.04E+01	4.72E+01	1.38E+02	1.64E+02
160.00	2.81E+02	2.21E+02	5.96E+02	6.84E+02
170.00	1.09E+03	8.61E+02	2.17E+03	2.44E+03
180.00	3.64E+03	2.88E+03	6.85E+03	7.57E+03
190.00	1.07E+04	8.49E+03	1.92E+04	2.09E+04
200.00	2.82E+04	2.24E+04	4.85E+04	5.24E+04
220.00	1.50E+05	1.20E+05	2.41E+05	2.56E+05
240.00	5.99E+05	4.82E+05	9.12E+05	9.60E+05
260.00	1.93E+06	1.56E+06	2.81E+06	2.94E+06
280.00	5.25E+06	4.26E+06	7.35E+06	7.64E+06
298.00	1.15E+07	9.36E+06	1.56E+07	1.62E+07

3. Computed Linear Free Energy Relationships

The half-lives computation using Polyrate described in Section 2 yielded the intrinsic reaction coordinates (IRCs) that have been augmented with the zero-point vibrational energies ZPVEs. For example, **Figure S3.1** illustrates the barrier widths (w_p) of the rotamerization of ***E-6a*** → ***Z*-6a**. "Collision energy" is given by half of the ZPVE (v_E) of the vibrational "reaction" mode (C–O bond rotations in the carboxylic motif) of the *E*-isomer towards the transition structure. The barrier widths of eight compounds, four for each series, are listed in Table S3.1, and the Bell−Evans−Polanyi (BEP) plot of the width against the computed reaction free energy is presented in Figure S3.2(a).

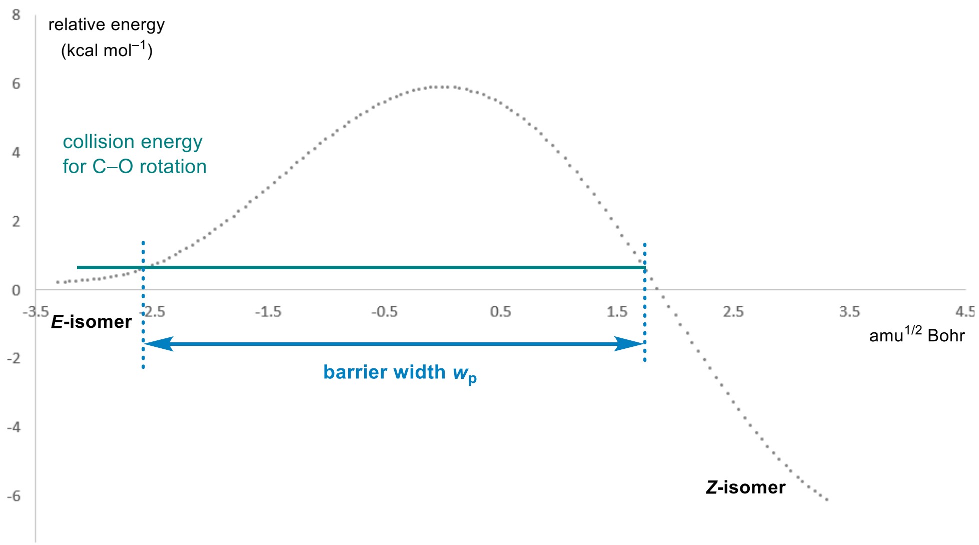

Figure S3.1. ZPVE-corrected IRC of the reaction ***E-6a*** ⟶ ***Z*-6a** computed at the CVT/SCT//MP2/cc-pVDZ level of theory using Polyrate Version 2017-c.[3]

Table S3.1. Computed (MP2/cc-pVDZ) free energy change (ΔG), free energy barrier height ($\Delta^{\ddagger}G$) and width (w_p) of $E \longrightarrow Z$ isomerization for deuterated carboxylic acids (ArCOOD). The widths were obtained from the ZPVE-corrected IRCs.

Compound	ΔG (kcal mol^{-1})	ν_E (cm^{-1})	w_p (u$^{1/2}$ Bohr)
5a. R=H, X=H	-6.6	357.6	4.421
5c. R=H, X=Me	-6.5	368.6	4.436
5d. R=H, X=Cl	-7.1	363.1	4.269
5e. R=H, X=CH_2F	-6.8	362.5	4.358
6a. R=Me, X=Me	-4.1	393.2	5.159
6c. R=Me, X=F	-4.4	393.2	5.089
6e. R=Me, X=Cl	-4.4	389.9	5.075
6f. R=Me, X=CN	-4.6	383.8	5.024

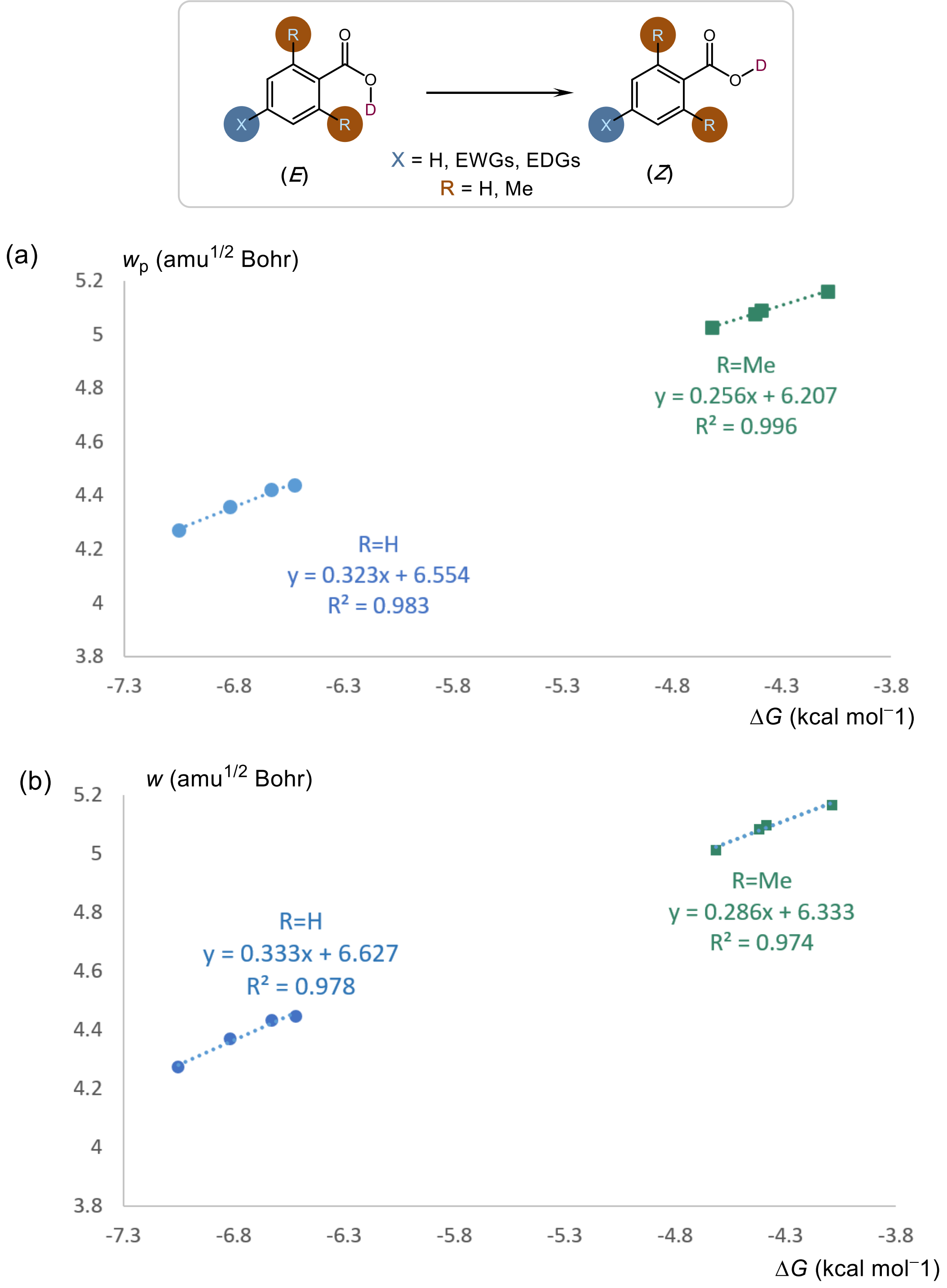

Figure S3.2. (a) BEP correlations for the data in **Table S3.1.** For R=H, X= Cl, CFH_2, H, Me; for R=Me, X= CN, Cl, F, H, Me; both in the ascending order of free energy change of isomerization. (b) BEP correlations for the same compounds as in (a), where the barrier widths (*w*) were obtained via the approximation presented in **Figure S3.3**.

To significantly expand the data set to map out multiple LFERs of the barrier width at a reasonable computational cost, we approximated the barrier width (*w*) obtained from the IRSs that have not been ZPVE-corrected (**Figure S3.3**). The BEP correlation of the approximated width for the same eight

compounds is shown in **Figure S3.2(b)**. The barrier widths obtained *via* the two method agree well (compare **Table S3.1** with **Table S3.3** below). The two plots in Figure **S3.2(a)** and **(b)** are also in good agreement: in both, the *ortho*-Me series display a smaller slope and a smaller intercept than the *ortho*-H series.

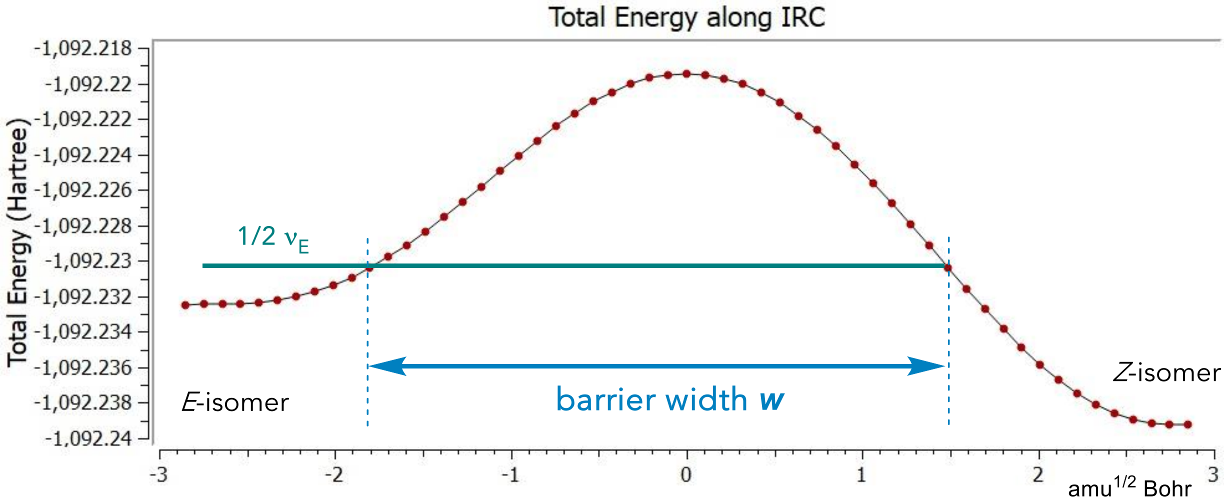

Figure S3.3. IRC of the reaction ***E*-4a** → ***Z*-4a** computed by connecting the rotamerization transition structure with the *E* and *Z* conformers at the MP2/cc-pVDZ level of theory. Note that only the provided relative energies of the stationary points are ZPVE corrected, while the IRC curve is not. The barrier widths (*w*) listed in **Tables S3.2** and **S3.3** were obtained from this approximation.

Table S3.2. Computed (MP2/cc-pVDZ) free energy change (Δ*G*), free energy barrier height ($\Delta^{\ddagger}G$) and width (*w*) of $E \rightarrow Z$ isomerization for protium carboxylic acids (ArCOOH). The widths were obtained from the approximation shown in **Figure S3.3**.

Compound	Δ*G* (kcal mol^{-1})	$\Delta^{\ddagger}G$ (kcal mol^{-1})	ν_E (cm^{-1})	*w* (u$^{1/2}$ Bohr)
**1a**. R=H, X=H	−6.6	5.0	496.4	3.188
**1b**. R=H, X=F	−7.0	4.6	492.0	3.077
**1c**. R=H, X=Me	−6.5	5.0	495.7	3.203
**1d**. R=H, X=Cl	−7.0	4.6	485.2	3.097
**1e**. R=H, X=CH_2F	−6.8	4.8	500.3	3.141
**1f**. R=H, X=CN	−7.4	4.5	480.9	3.036
**2a**. R=Me, X=H	−4.1	7.8	540.6	3.686
**2b**. R=Me, X=F	−4.4	7.5	547.7	3.632
**2c**. R=Me, X=Me	−4.1	7.8	544.7	3.678
**2d**. R=Me, X=NO_2	−4.6	7.4	532.4	3.588
**2e**. R=Me, X=Cl	−4.4	7.5	541.2	3.625
**2f**. R=Me, X=CN	−4.6	7.4	536.6	3.603
**2g**. R=Me, X=CCH	−4.3	7.6	544.4	3.638
**2h**. R=Me, X=AcNMe	−4.5	7.5	544.4	3.613
**3a**. R=iPr, X=H	−3.7	8.4	559.4	3.967
**3b**. R=iPr, X=F	−3.9	8.2	557.7	3.973
**3c**. R=iPr, X=CF_3	−4.1	8.1	561.6	3.928
**3d**. R=iPr, X=Me	−3.7	8.4	560.6	3.968
**3e**. R=iPr, X=NO_2	−4.1	8.0	559.1	3.915
**3f**. R=iPr, X=Cl	−3.9	8.2	559.9	3.965
**3g**. R=iPr, X=CN	−4.2	8.0	557.9	3.930
**4a**. R=CF_3, X=H	−4.1	7.2	498.5	3.608
**4b**. R=CF_3, X=Me	−4.0	7.2	493.9	3.630
**4c**. R=CF_3, X=Cl	−4.3	7.1	495.9	3.599
**4d**. R=CF_3, X=CN	−4.5	6.8	489.3	3.552

Table S3.3. Computed (MP2/cc-pVDZ) free energy change (Δ*G*), free energy barrier height ($\Delta^{‡}G$) and width (w) of $E \rightarrow Z$ isomerization for deuterated carboxylic acids (ArCOOD). The widths were obtained from the approximation shown in **Figure S3.3.**

Compound	Δ*G* (kcal mol^{-1})	$\Delta^{‡}G$ (kcal mol^{-1})	ν_E (cm^{-1})	*w* (u$^{1/2}$ Bohr)
**5a**. R=H, X=H	−6.6	5.2	357.6	4.433
**5b**. R=H, X=F	−7.0	4.9	362.4	4.276
**5c**. R=H, X=Me	−6.5	5.2	368.6	4.446
**5d**. R=H, X=Cl	−7.1	4.9	363.1	4.275
**5e**. R=H, X=CH_2F	−6.8	5.1	362.5	4.369
**5f**. R=H, X=CCH	−6.9	5.0	371.6	4.309
**6a**. R=Me, X=H	−4.1	8.1	391.4	5.162
**6b**. R=Me, X=F	−4.4	7.8	392.8	5.098
**6c**. R=Me, X=Me	−4.1	8.0	393.2	5.166
**6d**. R=Me, X=Br	−4.4	7.8	389.0	5.082
**6e**. R=Me, X=Cl	−4.4	7.8	389.9	5.084
**6f**. R=Me, X=CN	−4.6	7.7	383.8	5.012
**6h**. R=Me, X=NMe_2	−4.2	7.7	397.2	5.144
**6i**. R=Me, X=CHO	−4.4	7.9	386.2	5.093
**7a**. R=CF_3, X=H	−4.1	7.3	357.9	5.061
**7b**. R=CF_3, X=CF_3	−4.4	7.1	354.6	4.997
**7c**. R=CF_3, X=NO_2	−4.5	7.0	354.7	4.979
**7d**. R=CF_3, X=CN	−4.5	7.0	353.9	4.976
**7e**. R=CF_3, X=CCH	−4.1	7.4	357.1	5.060

4. Matrix Isolation and Computed IR Spectra

Equipment used in matrix isolation, photoexcitation, and IR measurement:

- Sumitomo cryostat system consisting of an RDK 408D2 closed-cycle refrigerator cold head and an F-70 compressor unit.
- Bruker Vertex 70 FTIR spectrometer
- 150 W Hamamatsu Xe(Hg) high-pressure lamp

A polished CsI window was mounted in the cold head's sample holder. The sample holder, connected with silicon diodes for temperature measurements, was covered by the vacuum shroud, which was equipped with KBr windows to allow for IR measurements. The sample and the host gas (argon, purity of 99.999%) were deposited at 15 K. All spectral data were collected at 11 K, except for one kinetic experiment, which was performed at 20 K. Argon was stored in a 2 L gas balloon, which was evacuated and filled three times before every experiment. All monodeuterated aryl carboxylic acids (ArCOOD) were obtained (from the respective commercial ArCOOH without further purification) and were purified with five re-crystallizations from D_2O (co-solvated with 1/3 volume of HPLC grade acetone to increase solubility).

Five carboxylic acids were studied, each was evaporated at r.t., mixed with Ar in the gas phase, condensed on the cold window of the matrix apparatus (CsI, 15 K) ca. 50 mm away from the substance reservoir. The evaporation time for each compound is shown below:

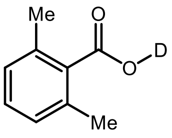

(6a) 8 h

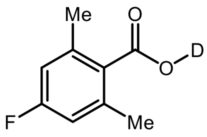

(6b) 8 h

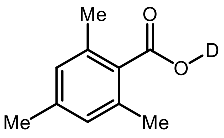

(6c) 20 h

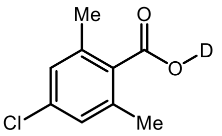

(6e) 36 h

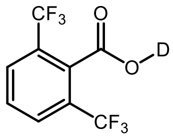

(7a) 3 h

The higher-lying *E*-isomers were generated photochemically by irradiation of the *Z*-isomer at 254 nm, for 30 min.

Table S4.1. Computed and experimental vibrational frequencies in Ar solid at 11 K for *E*-**6a** and *Z*-**6a** (wavenumbers [cm^{-1}], intensities [$km\ mol^{-1}$]).

MP2/cc-pVDZ (*Z*-6a) / cm^{-1}	Intensity / $km\ mol^{-1}$	Approximate Assignment	MP2/cc-pVDZ (*E*-6a) / cm^{-1}	Intensity / $km\ mol^{-1}$	Approximate Assignment	Ar matrix[a] / cm^{-1}
900.6	< 0.1	H-C-C-C torsion	900.8	< 0.1	H-C-C-C torsion	not observed
940.6	7.3	C-C-C bend + H-C-C bend	939.2	5.3	C-C-C bend + H-C-C bend	not observed
942.6	12.5	H-C-C-C torsion	941.9	6.5	H-C-C-C torsion	932.1 vw
1011.7	5.5	ring-CH_3 wag	1007.2	4.3	ring-CH_3 wag	not observed
1019.0	203.9	C-O-H bend	951.9	63.1	C-O-H bend	995.2 m (Z), 932.1 m (E)
1042.7	17.8	ring-CH_3 wag	1041.5	6.8	ring-CH_3 wag	1036.1 vw
1052.3	0.6	ring-CH_3 wag	1051.3	1.8	ring-CH_3 wag	not observed
1066.1	3.7	ring-CH_3 wag	1064.2	6.4	ring-CH_3 wag	1041.4 vw
1090.7	156.8	in-plane C-C ring stretch	1087.9	199.8	in-plane C-C ring stretch	1070.2 s
1139.5	59.6	H-C-C in-plane bend	1135.2	138.8	H-C-C in-plane bend	1120.3 m
1184.7	2.4	H-C-C in-plane bend	1184.3	1.7	H-C-C in-plane bend	1173.3 w
1269.8	7.9	H-C-C in-plane bend	1270.4	19.4	H-C-C in-plane bend	1276.3 w (Z), 1253.7 m (E)
1287.6	10.2	ring C-C stretch	1286.6	57.6	ring C-C stretch	1282.2 w (Z), 1266.3 m (E)
1341.2	588.3	C-O stretch	1318.0	719.9	C-O stretch	1298.3 vs (Z), 1274.2 vs (E)
1408.5	1.8	C-H wag (CH_3)	1409.0	2.1	C-H wag (CH_3)	not observed
1413.5	5.2	C-H wag (CH_3)	1415.0	4.5	C-H wag (CH_3)	1383.6 w
1454.1	2.7	C-H twist (CH_3)	1457.5	4.8	C-H twist (CH_3)	1386.3 w
1465.8	121.4	C-H sciss (CH_3)	1462.0	81.1	C-H sciss (CH_3)	1426.4 m
1481.4	22.0	C-H sciss (CH_3)	1479.6	17.8	C-H sciss (CH_3)	1431.7 w/m
1484.6	6.4	C-H sciss (CH_3)	1484.4	6.6	C-H sciss (CH_3)	1456.7 w
1486.5	26.9	C-H sciss (CH_3)	1491.4	20.9	C-H sciss (CH_3)	1489.3 w
1505.7	52.8	C-H sciss (CH_3) + C-H rock	1506.2	35.7	C-H sciss (CH_3) + C-H rock	1452 m
1507.1	26.0	C-H sciss (CH_3) + C-H rock	1506.7	40.6	C-H sciss (CH_3) + C-H rock	1473.8 m
1641.4	6.0	C=C stretch	1639.2	5.9	C=C stretch	1601.1 w
1651.5	19.9	C=C stretch	1650.9	13.5	C=C stretch	1603.9 w
1815.2	493.7	C=O stretch	1843.0	469.7	C=O stretch	1748.2 vs (Z), 1785.1 vs (E)
2721.6	76.2	O-D stretch	2777.4	41.0	O-D stretch	2619.1 m/s (Z), 2660.1 m/s (E)

3088.5	52.9	C-H stretch (CH_3, sym)	3077.9	27.9	C-H stretch (CH_3, sym)	2939.5 w
3089.0	4.5	C-H stretch (CH_3, sym)	3085.5	22.2	C-H stretch (CH_3, sym)	2949.8 w
3181.6	14.2	C-H stretch (CH_3, asym)	3164.5	14.0	C-H stretch (CH_3, asym)	2978.6 w
3184.1	14.8	C-H stretch (CH_3, asym)	3176.6	11.1	C-H stretch (CH_3, asym)	2993.6 w
3189.6	11.5	C-H stretch (CH_3, asym)	3190.1	9.2	C-H stretch (CH_3, asym)	3038.2 w
3193.8	9.9	C-H stretch (CH_3, asym)	3195.1	9.6	C-H stretch (CH_3, asym)	3045.2 w
3209.1	0.1	C-H in-plane stretch, asym)	3209.7	0.2	C-H in-plane stretch, asym)	not observed
3218.8	21.5	C-H in-plane stretch, asym)	3218.5	18.4	C-H in-plane stretch, asym)	3057.7 w
3235.7	20.0	C-H in-plane stretch, sym)	3236.7	17.3	C-H in-plane stretch, sym)	3084.7 w

a: rel. experimental intensities (vw = very weak, w = weak, m = middle, s = strong, vs = very strong)

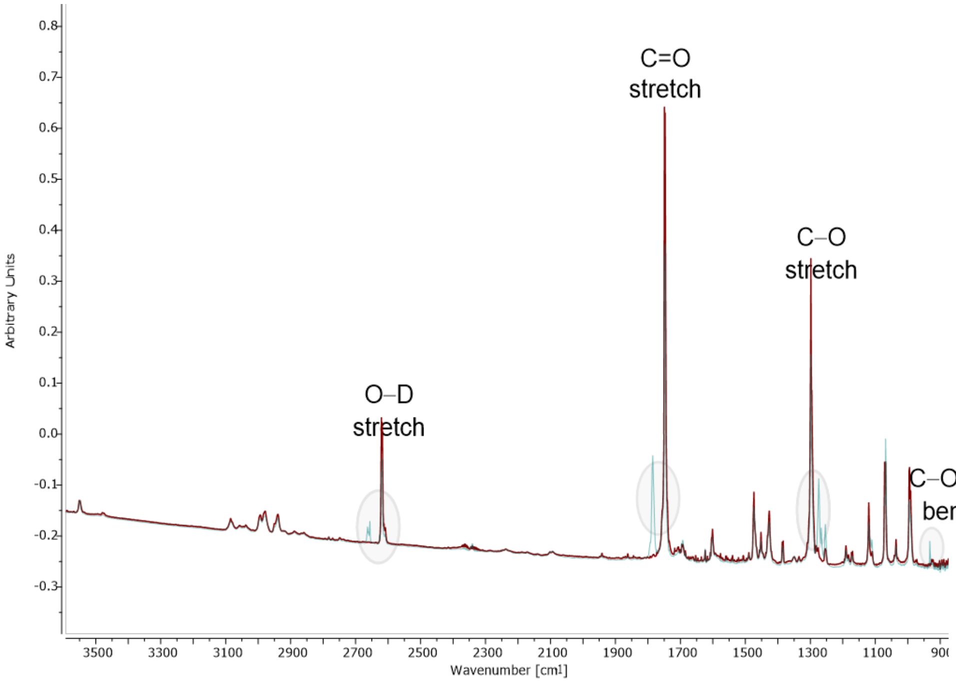

Figure S4.1: Matrix IR spectrum of acid *Z*-**6a** in Ar before (red) and after irradiation at 254 nm (green).

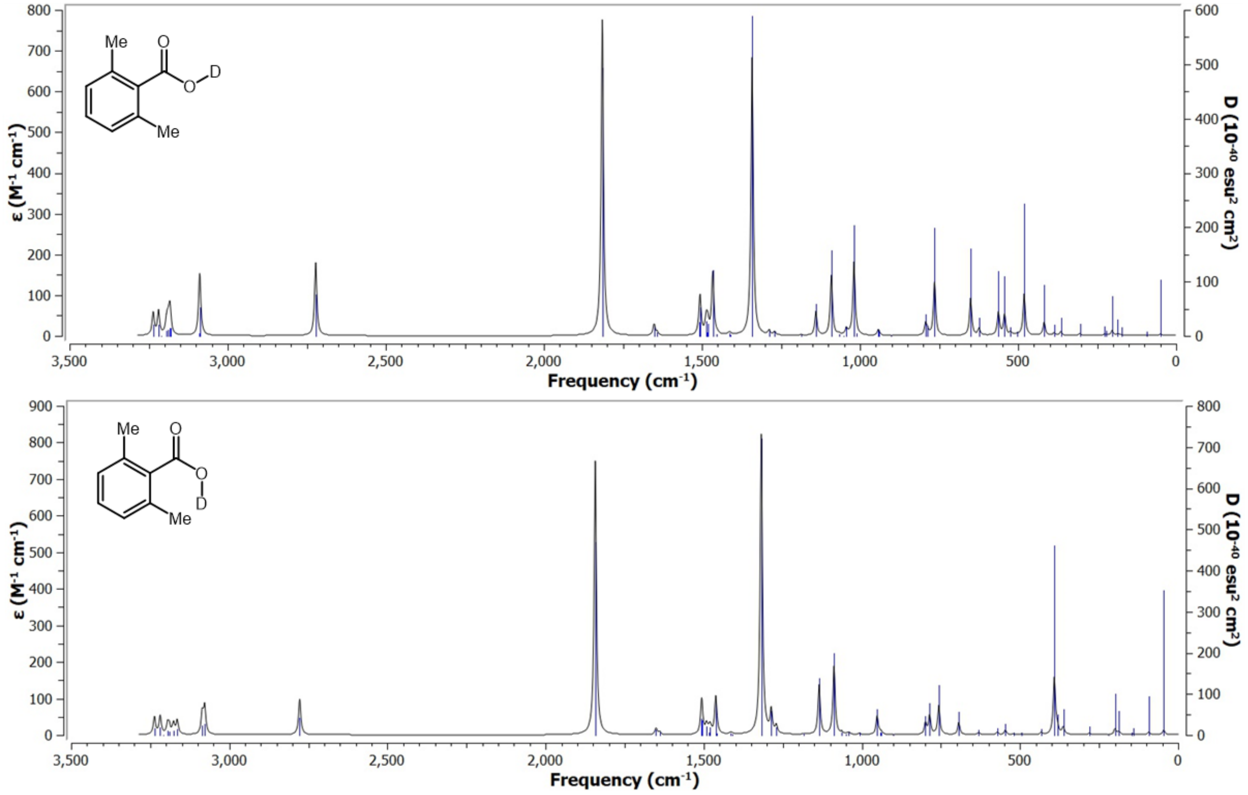

Figure S4.2. MP2/cc-pVDZ Harmonic IR-spectra of *E*- and *Z*-isomers of **6a**.

Table S4.2. Computed and experimental vibrational frequencies in Ar solid at 11 K for *E*-**6b** and *Z*-**6b** (wavenumbers [cm^{-1}], intensities [km mol^{-1}]).

MP2/cc-pVDZ (*Z*-6b) / cm^{-1}	Intensity / km mol^{-1}	Approximate Assignment	MP2/cc-pVDZ (*E*-6b) / cm^{-1}	Intensity / km mol^{-1}	Approximate Assignment	Ar matrix / cm^{-1}
869.2	138.4	H-C-C-C torsion	866.5	125.5	H-C-C-C torsion	862.9 s, 870.7 m
877.9	0.3	H-C-C-C torsion	878.6	4.8	H-C-C-C torsion	not observed
979.4	30.3	ring-CH_3 wag + H-C-C bend	978.5	9.9	ring-CH_3 wag + H-C-C bend	979.5w
993.8	41.4	ring-CH_3 wag + H-C-C bend	988.7	27.4	ring-CH_3 wag + H-C-C bend	993.6 m
1019.2	205.4	C-O-H bend	949.5	73.8	C-O-H bend	977.1 m (Z), 932.5 (E)
1042.5	14.4	ring-CH_3 wag	1041.2	4.8	ring-CH_3 wag	not observed
1050.3	64.1	ring-CH_3 wag	1048.1	88.5	ring-CH_3 wag	1031.3 m
1054.2	38.4	ring-CH_3 wag	1052.1	12.6	ring-CH_3 wag	1035.6 w
1066.2	1.7	ring-CH_3 wag	1063.8	9.0	ring-CH_3 wag	1039.8 w
1104.9	130.5	in-plane C-C ring stretch	1103.7	229.1	in-plane C-C ring stretch	1078.5 w/m
1169.7	194.2	H-C-C in-plane bend	1167.1	234.7	H-C-C in-plane bend	1144.5 s (Z), 1137.9 s (E)
1226.2	0.4	H-C-C in-plane bend	1224.6	2.7	H-C-C in-plane bend	1219.7 vw
1334.8	376.7	C-O stretch	1313.4	690.4	C-O stretch	1285.9 vs (Z), 1267.2 vs (E)
1365.3	532.9	F-C-C in-plane bend + C-C stretch	1363.5	371.3	F-C-C in-plane bend + C-C stretch	1312.9 vs
1408.8	5.7	C-H wag (CH_3)	1409.0	0.4	C-H wag (CH_3)	not observed
1415.4	2.4	C-H wag (CH_3)	1416.9	3.5	C-H wag (CH_3)	1341.1 w
1452.6	9.8	C-H twist (CH_3)	1457.5	13.7	C-H twist (CH_3)	1384.3 w/m
1458.8	1.2	F-C-C bend + C-H sciss (CH_3)	1458.5	1.9	F-C-C bend + C-H sciss (CH_3)	1398.6 w
1470.0	113.1	C-H sciss (CH_3)	1466.6	57.6	C-H sciss (CH_3)	1432.8 m
1481.3	22.3	C-H sciss (CH_3)	1479.6	16.8	C-H sciss (CH_3)	1446.2 w/m
1485.5	17.4	C-H sciss (CH_3)	1491.6	23.0	C-H sciss (CH_3)	1459.7 m
1501.9	25.4	C-H sciss (CH_3)	1503.5	20.1	C-H sciss (CH_3)	1472.1 w
1522.4	79.1	C-H sciss (CH_3) + H-C-C bend	1521.5	111.8	C-H sciss (CH_3) + H-C-C bend	1483.1 m/s
1656.9	37.2	C=C stretch	1653.8	39.4	C=C stretch	1596.7 m
1671.0	267.3	C=C stretch	1669.7	234.7	C=C stretch	1609.6 s
1814.2	518.1	C=O stretch	1841.6	482.1	C=O stretch	1747.5 vs (Z), 1786.5 vs (E)
2722.9	79.9	O-D stretch	2779.2	40.7	O-D stretch	2608.2 s (Z, site 1), 2619.9 (Z. site 2), 2655.2 (E, site 1), 2665.3 (E, site 2)
3092.3	46.7	C-H stretch (CH_3, sym)	3079.7	24.0	C-H stretch (CH_3, sym)	2937.2 w
3092.6	3.5	C-H stretch (CH_3, sym)	3088.9	19.2	C-H stretch (CH_3, sym)	2947.6 w

3187.2	12.5	C-H stretch (CH_3, asym)	3166.9	12.9	C-H stretch (CH_3, asym)	2983.4 w
3190.1	13.5	C-H stretch (CH_3, asym)	3180.9	9.8	C-H stretch (CH_3, asym)	2992.4 w
3192.7	11.1	C-H stretch (CH_3, asym)	3194.8	8.2	C-H stretch (CH_3, asym)	3061.9 vw
3196.4	10.3	C-H stretch (CH_3, asym)	3198.6	9.8	C-H stretch (CH_3, asym)	3100.2 vw
3241.6	3.2	C-H in-plane stretch, asym)	3240.0	2.2	C-H in-plane stretch, asym)	3202.3 vw
3242.9	2.2	C-H in-plane stretch, sym)	3242.0	1.7	C-H in-plane stretch, sym)	not observed

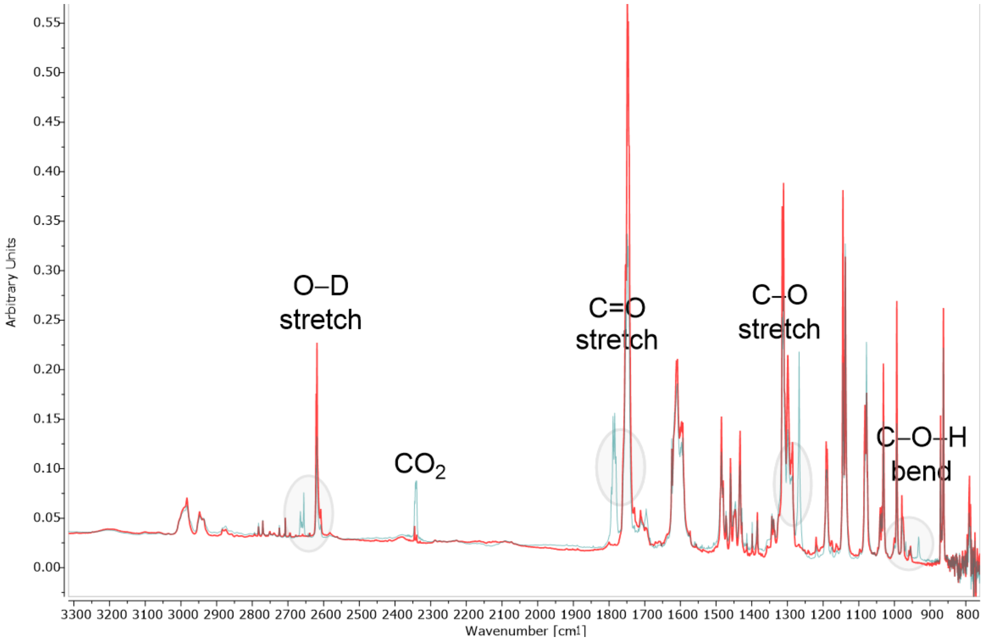

Figure S4.3: Matrix IR spectrum of acid *Z*-**6b** in Ar before (red) and after irradiation at 254 nm (green).

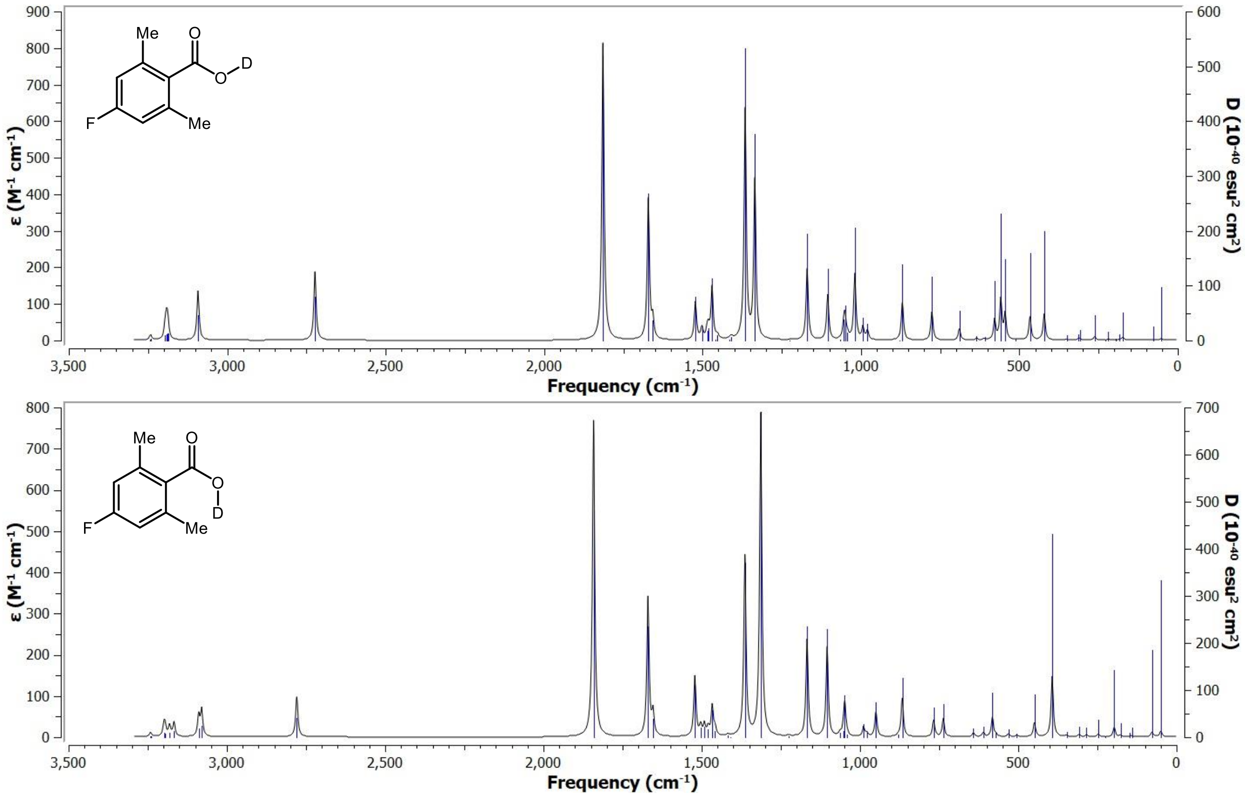

Figure S4.4. MP2/cc-pVDZ Harmonic IR-spectra of *E*- and *Z*-isomers of **6b**.

Table S4.3. Computed and experimental vibrational frequencies in Ar solid at 11 K for *E*-**6c** and *Z*-**6c** (wavenumbers [cm^{-1}], intensities [$km\ mol^{-1}$]).

MP2/cc-pVDZ (*Z*-6c) / cm^{-1}	Intensity / $km\ mol^{-1}$	Approximate Assignment	MP2/cc-pVDZ (*E*-6c) / cm^{-1}	Intensity / $km\ mol^{-1}$	Approximate Assignment	Ar matrix / cm^{-1}
853.6	49.9	H-C-C-C torsion	851.3	48.8	H-C-C-C torsion	out of range
892.0	0.1	H-C-C-C torsion	892.4	0.2	H-C-C-C torsion	out of range
957.3	12.2	ring-CH_3 wag + C-O-H bend	949.0	63.2	C-O-H bend	998.9 m/s (Z), 940.9 (E)
977.9	1.5	ring-CH_3 wag + C-C-C bend	957.5	11.2	ring-CH_3 wag + C-O-H bend	not observed
1018.5	222.5	C-O-H bend	973.4	1.3	ring-CH_3 wag + C-C-C bend	not observed
1033.7	16.0	ring-CH_3 wag	1032.7	12.1	ring-CH_3 wag	1035.6 w
1037.9	9.6	ring-CH_3 wag	1037.4	4.1	ring-CH_3 wag	1040.5 w
1042.6	10.8	ring-CH_3 wag	1039.9	14.2	ring-CH_3 wag	1045.5 w
1051.7	0.3	ring-CH_3 wag	1050.0	5.4	ring-CH_3 wag	not observed
1052.2	9.7	C-H wag (CH_3)	1051.7	8.0	C-H wag (CH_3)	not observed
1066.4	3.0	C-H wag (CH_3)	1064.3	7.9	C-H wag (CH_3)	not observed
1099.4	205.2	in-plane H-C-C bend + C-C bend	1098.2	303.0	in-plane H-C-C bend + C-C bend	1079.3 s
1201.2	40.9	H-C-C in-plane bend	1196.8	84.9	H-C-C in-plane bend	1160.8 m
1261.4	2.5	H-C-C in-plane bend	1259.9	6.8	H-C-C in-plane bend	1177.9 m
1332.9	284.5	C-O stretch + CH_3 wag	1314.6	708.2	C-O stretch	1301.9 vs (Z), 1275.6 (E)
1349.0	348.5	C-O stretch + CH_3 wag	1343.9	93.0	CH_3 wag + C-C stretch	1285.4 m
1404.9	1.8	C-H wag (CH_3)	1405.3	2.1	C-H wag (CH_3)	1381.5 vw
1408.0	4.0	C-H wag (CH_3)	1408.5	0.8	C-H wag (CH_3)	1387.3 vw
1416.5	0.4	C-H wag (CH_3)	1417.9	0.6	C-H wag (CH_3)	not observed
1440.3	5.4	C-H wag (CH_3)	1439.4	2.4	C-H wag (CH_3)	not observed
1453.6	1.7	C-H sciss (CH_3)	1458.1	3.5	C-H sciss (CH_3)	not observed
1469.0	134.2	C-H sciss (CH_3)	1466.2	87.2	C-H sciss (CH_3)	1404.6 m
1481.0	21.5	C-H twist (CH_3)	1479.3	18.7	C-H twist (CH_3)	1431.4 m
1483.5	21.9	C-H sciss (CH_3)	1482.6	14.7	C-H sciss (CH_3)	1446.0 w
1485.1	9.5	C-H sciss (CH_3)	1490.2	18.1	C-H sciss (CH_3)	1456.9 w
1492.0	12.9	C-H sciss (CH_3)	1494.0	16.7	C-H sciss (CH_3)	1464.9 w
1501.0	75.4	C-H sciss (CH_3)	1502.1	63.0	C-H sciss (CH_3)	1488.8 w
1521.6	11.8	C-H sciss (CH_3) + H-C-C bend	1520.5	14.9	C-H sciss (CH_3) + H-C-C bend	1576.4 w
1633.4	9.1	C=C stretch	1631.2	7.3	C=C stretch	not observed
1668.1	57.7	C=C stretch	1666.8	46.7	C=C stretch	1597.3 m
1814.1	516.6	C=O stretch	1842.1	490.1	C=O stretch	1741.5 vs (Z), 1780.8 (E)

2722.3	78.5	O-D stretch	2778.4	39.2	O-D stretch	2605.5 s (Z site 1), 2614.8 (Z site 2), 2641.9 (E site 1), 2653.2 (E site 2)
3075.4	36.1	C-H stretch (CH_3, sym)	3075.9	34.0	C-H stretch (CH_3, sym)	2936.7 w/m
3088.1	55.9	C-H stretch (CH_3, sym)	3077.3	30.3	C-H stretch (CH_3, sym)	2965.3 w/m
3088.5	4.0	C-H stretch (CH_3, sym)	3085.2	23.5	C-H stretch (CH_3, sym)	2990.4 w/m
3163.6	15.5	C-H stretch (CH_3, asym)	3162.1	14.4	C-H stretch (CH_3, asym)	3015.7 vw
3181.1	13.8	C-H stretch (CH_3, asym)	3165.8	14.6	C-H stretch (CH_3, asym)	3029.5 vw
3183.6	12.5	C-H stretch (CH_3, asym)	3176.1	11.5	C-H stretch (CH_3, asym)	3042.2 vw
3184.0	12.9	C-H stretch (CH_3, asym)	3184.8	11.0	C-H stretch (CH_3, asym)	3082.7 vw
3188.2	9.4	C-H stretch (CH_3, asym)	3189.7	6.4	C-H stretch (CH_3, asym)	3105.5 vw
3192.4	6.2	C-H stretch	3194.0	4.2	C-H stretch	not observed
3199.8	26.8	C-H stretch	3199.6	26.3	C-H stretch	3157.9 w/m
3201.3	17.3	C-H stretch	3200.5	13.7	C-H stretch	3194.4 w

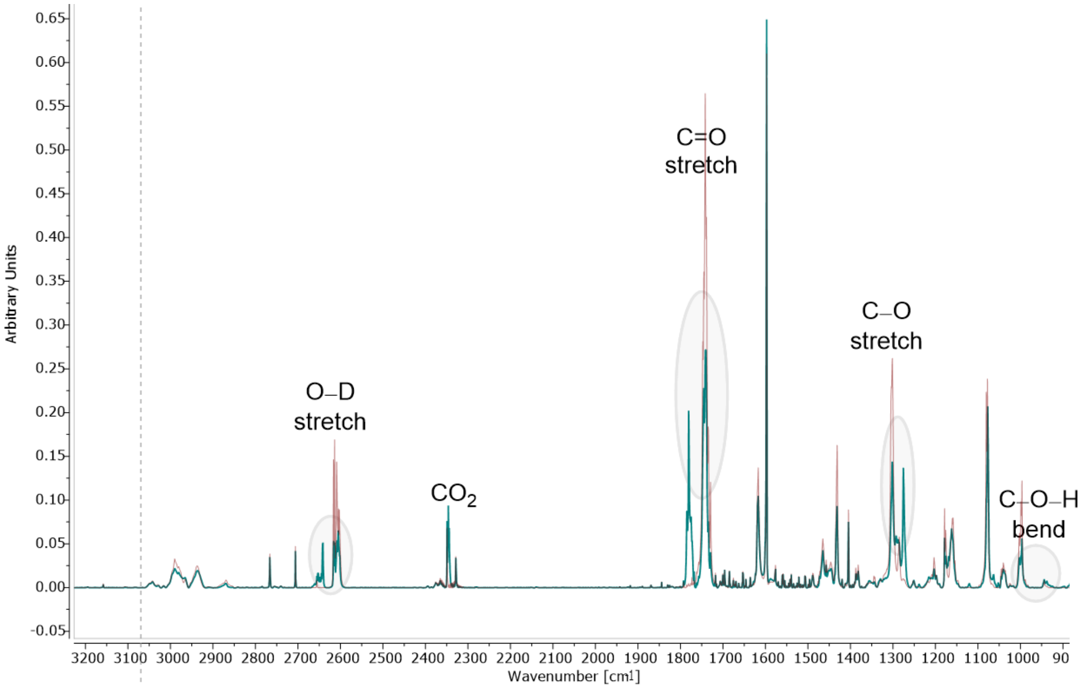

Figure S4.5: Matrix IR spectrum of acid *Z*-**6c** in Ar before (red) and after irradiation at 254 nm (green).

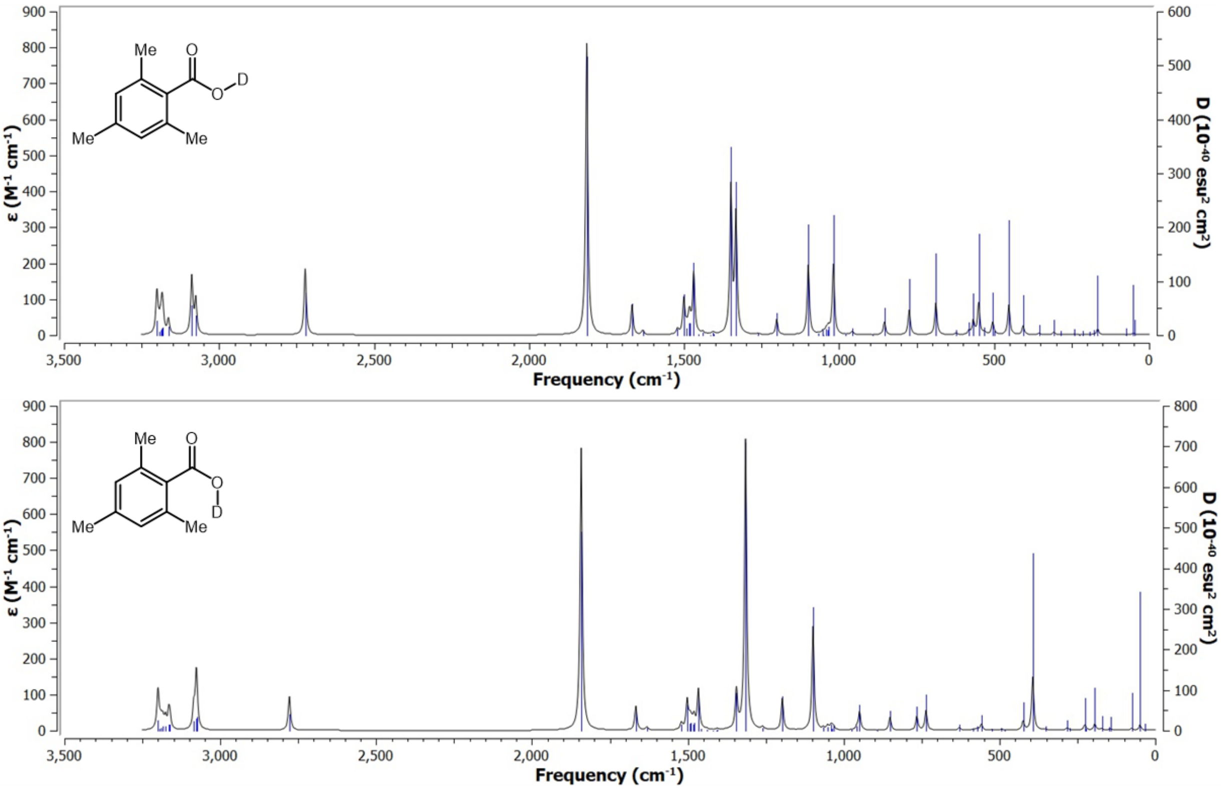

Figure S4.6. MP2/cc-pVDZ Harmonic IR-spectra of *E*- and *Z*-isomers of **6c**.

Table S4.4. Computed and experimental vibrational frequencies in Ar solid at 11 K for *E*-**6e** and *Z*-**6e** (wavenumbers [cm^{-1}], intensities [km mol^{-1}]).

**MP2/cc-pVDZ (*Z*-6e) / cm^{-1}**	**Intensity / km mol^{-1}**	**Approximate Assignment**	**MP2/cc-pVDZ (*E*-6e) / cm^{-1}**	**Intensity / km mol^{-1}**	**Approximate Assignment**	**Ar matrix / cm^{-1}**
853.7	78.6	H-C-C-C torsion	851.2	75.0	H-C-C-C torsion	861.5 s
876.8	0.5	H-C-C-C torsion	877.4	0.5	H-C-C-C torsion	not observed
897.1	216.2	ring-CH_3 wag + Cl-C-C bend	897.0	185.7	ring-CH_3 wag + Cl-C-C bend	882.5 s
974.7	17.7	ring-CH_3 wag + H-C-C bend	973.8	4.5	ring-CH_3 wag + H-C-C bend	870.2 vw
1019.0	199.8	C-O-H bend	949.2	77.7	C-O-H bend	993.3 w/m
1024.1	28.9	ring-CH_3 wag	1020.0	41.2	ring-CH_3 wag	1008.9 w
1041.3	15.7	ring-CH_3 wag	1040.2	5.5	ring-CH_3 wag	1034.2 vw
1051.8	0.3	ring-CH_3 wag	1050.7	1.4	ring-CH_3 wag	1036.8 vw
1066.8	3.7	ring-CH_3 wag	1064.8	7.7	ring-CH_3 wag	1040.9 vw
1098.0	166.3	ring C-C bend	1096.0	234.5	ring C-C bend	1073.2 s
1154.0	142.4	H-C-C in-plane bend	1148.9	206.3	H-C-C in-plane bend	1122.2 s
1245.2	1.1	H-C-C in-plane bend	1243.6	4.7	H-C-C in-plane bend	1195.1 w
1301.2	32.4	Cl-C-C in-plane bend + C-C stretch	1299.8	69.2	Cl-C-C in-plane bend + C-C stretch	1185.3 m/s
1340.6	660.8	C-O stretch	1319.1	800.1	C-O stretch	1296.4 s (Z) 1271.0 (E)
1408.2	3.8	C-H wag (CH_3)	1408.2	1.2	C-H wag (CH_3)	not observed
1413.2	0.8	C-H wag (CH_3)	1414.6	0.6	C-H wag (CH_3)	not observed
1442.0	12.0	C-H twist (CH_3)	1442.0	12.2	C-H twist (CH_3)	1335.7 w
1457.2	10.4	C-H sciss (CH_3)	1459.4	11.1	C-H sciss (CH_3)	1361.6 w
1466.8	144.6	C-H sciss (CH_3)	1462.9	77.8	C-H sciss (CH_3)	1384.2 m
1481.1	22.1	C-H sciss (CH_3)	1479.1	18.2	C-H sciss (CH_3)	1398.5 w
1485.1	17.4	C-H sciss (CH_3)	1490.8	23.2	C-H sciss (CH_3)	1427.8 w
1499.0	38.6	C-H sciss (CH_3)	1500.6	33.5	C-H sciss (CH_3)	1456.9 w
1506.0	34.0	C-H sciss (CH_3) + H-C-C bend	1505.7	53.7	C-H sciss (CH_3) + H-C-C bend	1586.9 w/m
1631.1	21.8	C=C stretch	1628.9	23.8	C=C stretch	1589.5 w
1642.8	170.4	C=C stretch	1642.4	141.4	C=C stretch	1593.3 m
1814.6	516.6	C=O stretch	1841.7	485.6	C=O stretch	1751.4 s (Z), 1788.7 (E)
2722.1	84.3	O-D stretch	2778.2	41.0	O-D stretch	2619.3 s (Z, site 1), 2639.2 (Z, site 2), 2655.9 (E, site 1), 2666.5 (Z, site 2)
3091.7	45.1	C-H stretch (CH_3, sym)	3080.0	23.9	C-H stretch (CH_3, sym)	2942.9 w
3092.1	4.2	C-H stretch (CH_3, sym)	3088.2	19.0	C-H stretch (CH_3, sym)	2989.7 w

3186.4	12.1	C-H stretch (CH_3, asym)	3167.6	12.1	C-H stretch (CH_3, asym)	2996.1 w
3189.1	13.0	C-H stretch (CH_3, asym)	3180.3	9.3	C-H stretch (CH_3, asym)	not observed
3193.4	10.4	C-H stretch (CH_3, asym)	3194.0	8.0	C-H stretch (CH_3, asym)	not observed
3197.1	9.5	C-H stretch (CH_3, asym)	3198.8	9.1	C-H stretch (CH_3, asym)	not observed
3235.9	1.9	C-H in-plane stretch, asym)	3234.3	1.2	C-H in-plane stretch, asym)	not observed
3236.9	1.8	C-H in-plane stretch, sym)	3236.3	1.4	C-H in-plane stretch, sym)	not observed

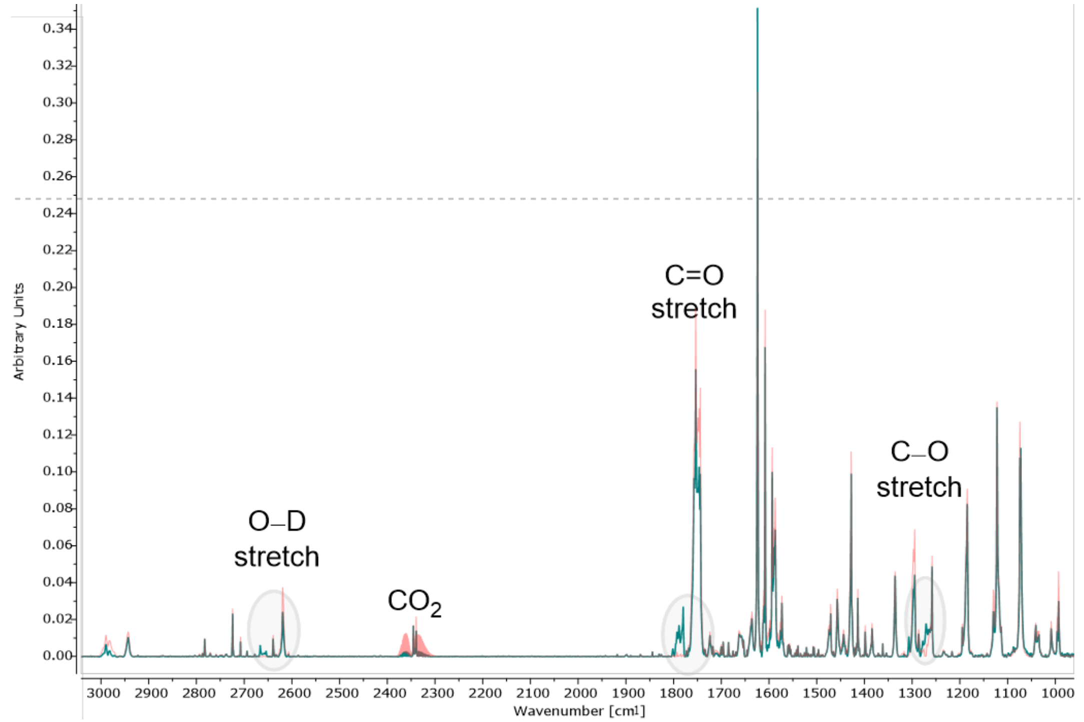

Figure S4.7. Matrix IR spectrum of acid *Z*-**6e** in Ar before (red) and after irradiation at 254 nm (green).

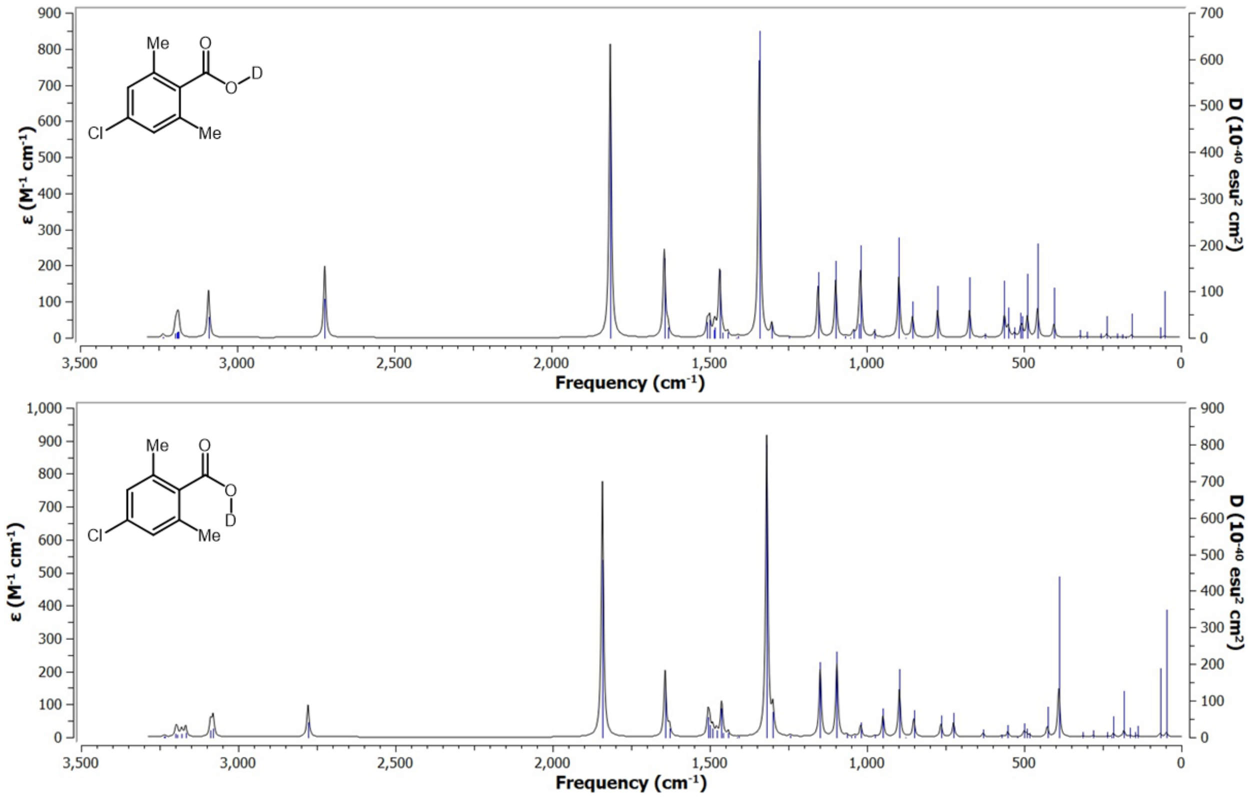

Figure S4.8. MP2/cc-pVDZ Harmonic IR-spectra of *E*- and *Z*-isomers of **6e**.

Table S4.5. Computed and experimental vibrational frequencies in Ar solid at 11 K for *E*-**7a** and *Z*-**7a** (wavenumbers [cm^{-1}], intensities [km mol^{-1}]).

**MP2/cc-pVDZ (*Z*-7a) / cm^{-1}**	**Intensity / km mol^{-1}**	**Approximate Assignment**	**MP2/cc-pVDZ (*E*-7a) / cm^{-1}**	**Intensity / km mol^{-1}**	**Approximate Assignment**	**Ar matrix / cm^{-1}**
931.4	0.2	H-C-C-C torsion	934.0	0.1	H-C-C-C torsion	not observed
968.9	3.1	H-C-C-C torsion	971.6	8.4	H-C-C-C torsion	not observed
1019.8	175.2	C-O-H bend	959.0	96.2	C-O-H bend	992.8 vw
1067.9	29.4	C-C-C bend + H-C-C bend	1065.0	8.8	C-C-C bend + H-C-C bend	1061.8 vw
1097.9	218.0	H-C-C in-plane bend	1096.8	224.5	H-C-C in-plane bend	1077.3 m
1152.3	43.9	H-C-C in-plane bend	1150.8	81.0	H-C-C in-plane bend	1150.6 w
1160.7	197.9	H-C-C in-plane bend	1158.1	243.2	H-C-C in-plane bend	1159.1 m/s
1209.8	33.3	H-C-C in-plane bend	1190.9	592.7	H-C-C bend + C-F stretch	1161.8 s
1220.1	445.4	C-F stretch	1211.6	159.4	H-C-C in-plane bend	1166.9 s
1224.9	1130.8	H-C-C bend + C-F stretch	1230.0	796.5	C-F stretch	1177.2 s
1234.3	872.5	H-C-C bend + C-F stretch	1233.7	773.3	C-C-C bend + C-C-H bend	1190.4 s
1237.0	879.4	H-C-C bend + C-F stretch	1254.5	772.4	H-C-C bend + C-F stretch	1193.6 s
1325.6	1189.5	H-C-C in-plane bend	1326.1	1046.7	C-O stretch	1212.9 vs
1351.7	990.1	C-O stretch + H-C-C bend	1327.0	1464.0	C-O stretch + H-C-C bend	1302.2 vs
1389.6	370.0	C-(CF_3) stretch + H-C-C bend	1389.6	415.0	C-(CF_3) stretch + H-C-C bend	1309.6 m/s
1487.4	34.3	C=C stretch + H-C-C bend	1484.0	45.2	C=C stretch	1347.9 m
1506.1	36.6	C=C stretch + H-C-C bend	1500.2	50.5	C=C stretch + H-C-C bend	1478.0 w
1512.2	71.3	C=C stretch + H-C-C bend	1506.8	26.0	C=C stretch + H-C-C bend	not observed
1649.9	61.9	C=C stretch	1645.6	62.6	C=C stretch	1600.9 w
1659.6	2.3	C=C stretch	1658.4	1.9	C=C stretch	not observed
1839.4	501.5	C=O stretch	1853.4	472.9	C=O stretch	1779.0 s (Z), 1800.8 (E)
2723.8	93.1	O-D stretch	2784.8	66.6	O-D stretch	2617.1 s (Z), 2661.9 (E)
3239.3	2.8	C-H stretch (CH_3, asym)	3240.6	2.2	C-H stretch (CH_3, asym)	not observed
3263.6	0.2	C-H stretch (CH_3, asym)	3264.1	0.3	C-H stretch (CH_3, asym)	not observed
3267.8	1.0	C-H in-plane stretch, sym)	3268.2	0.7	C-H in-plane stretch, sym)	not observed

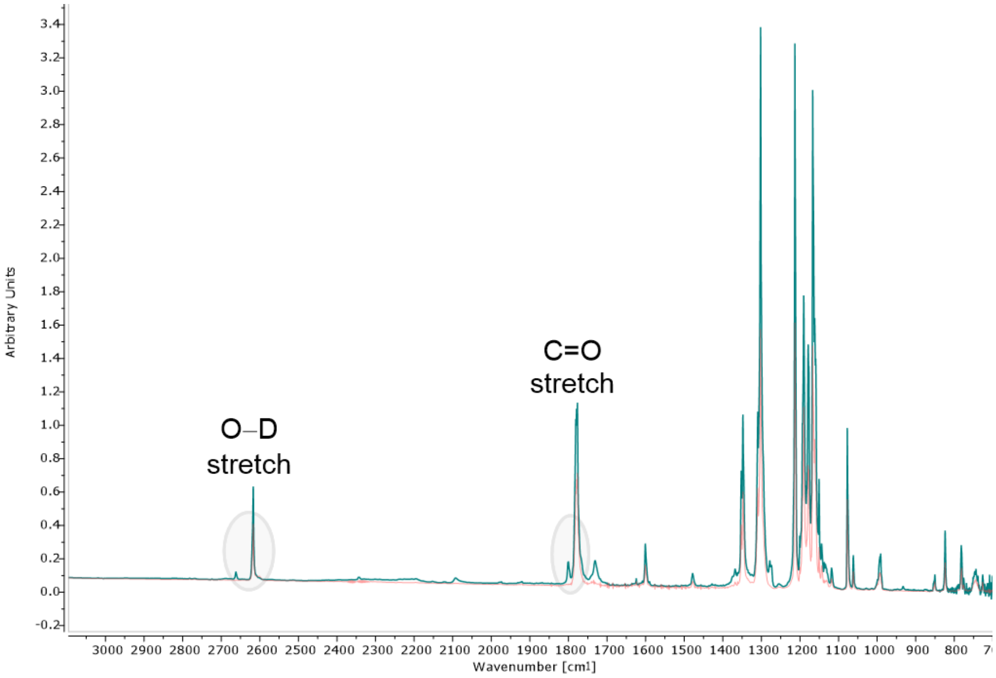

Figure S4.9. Matrix IR spectrum of acid *Z*-**7a** in Ar before (red) and after irradiation at 254 nm (green).

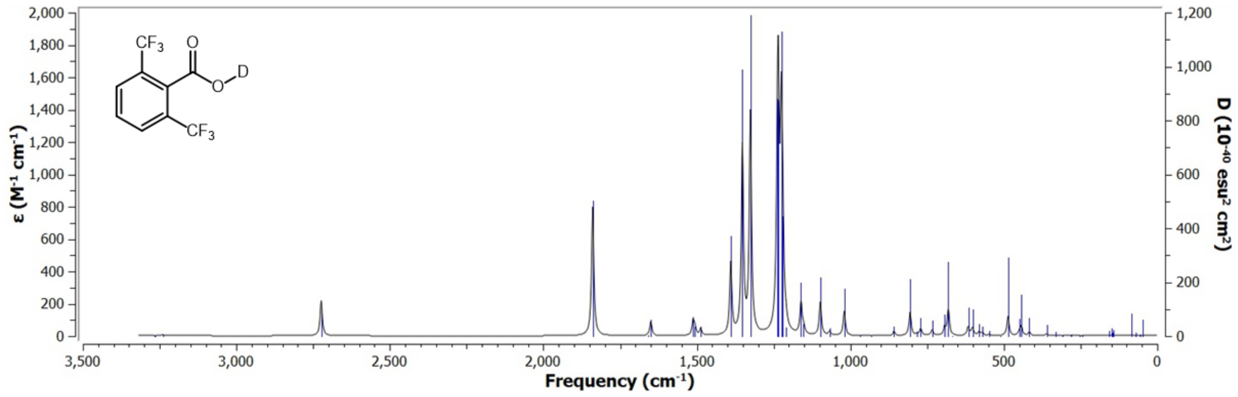

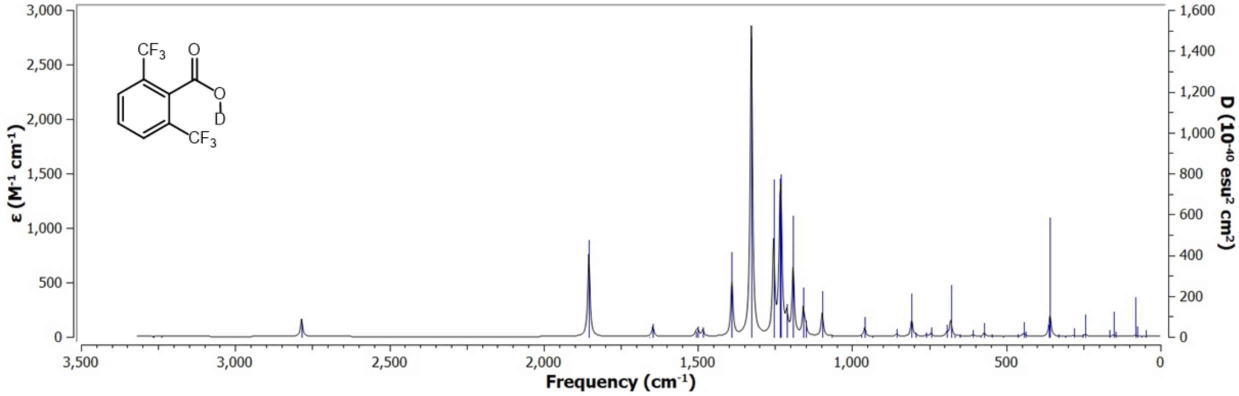

Figure S4.10. MP2/cc-pVDZ Harmonic IR-spectra of *E*- and *Z*-isomers of **7a**

5. Matrix Isolation Kinetic Measurements

As in our previous matrix isolation study on benzoic acid derivatives (compounds **5a**, **5c**, and **5d**), the characteristic C=O IR stretching bands were quantitatively monitored to determine the $E \rightarrow Z$ isomerization rate constants.[2] The C=O peak intensities of the *E*-isomer ([*E*]) and the *Z*-isomer ([*Z*]) were both monitored. The logarithm of the quotient ln([*E*]/([*E*]+[*Z*])) was then plotted against *t* to obtain the first-order decay constant *k*, *i.e.*, as ln([*E*]/([*E*]+[*Z*])) = k · *t* + const. The half-life $t_{1/2(exp)}$ equals ln(2)/*k*. Note that very low extinction values *E*, which can hardly be told apart from the baseline, lead to values of [*E*] which are afflicted with a large error. These data points were therefore excluded from the fitting procedure and are thus not shown in the kinetic plots.

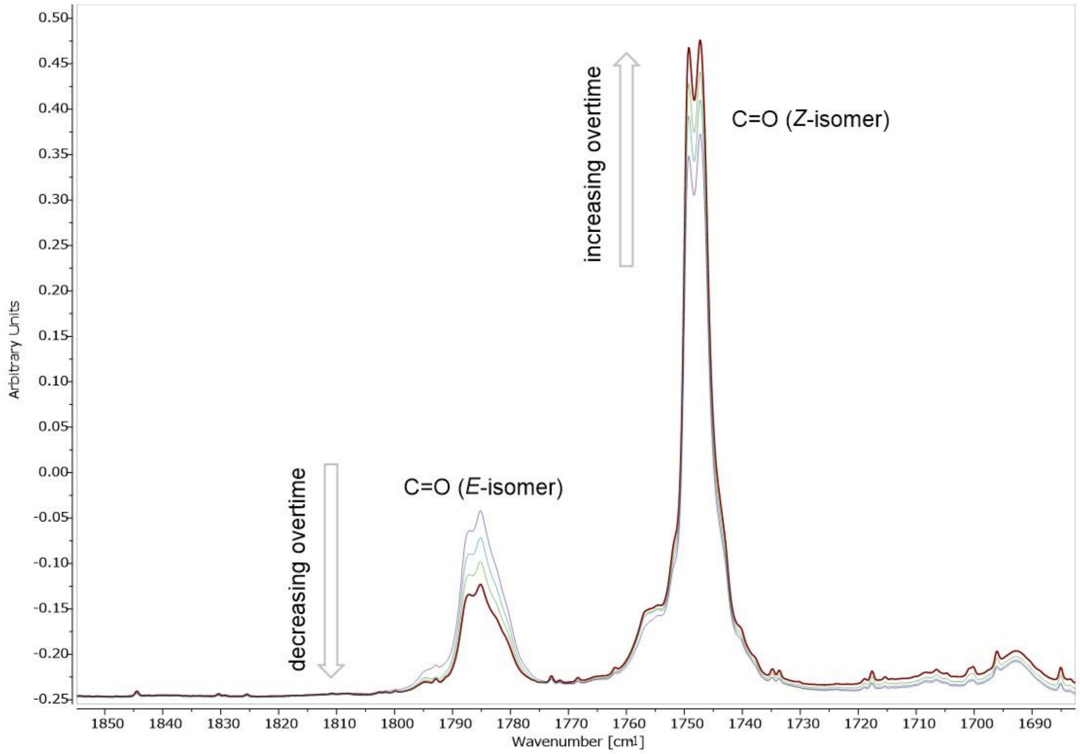

Figure S5.1. Decay of the matrix isolated *E*-**6a** (Ar, 11 K) as monitored by the evolution of intensities of the two C=O bands at 1748.2 and 1785.1 cm^{-1} over time.

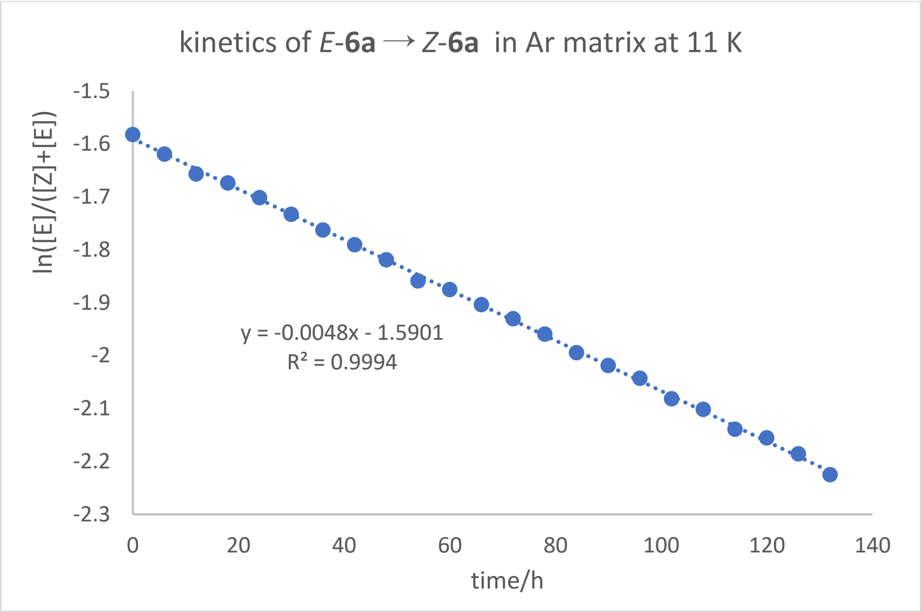

Figure S5.2. Kinetic evaluation of *E*-**6a** (1785.1 cm^{-1}) ⟶ *Z*-**6a** (1748.2 cm^{-1}) in Ar, 11 K; half-life $t_{1/2(exp)}$ (**6a**) = (8660 ± 60) min.

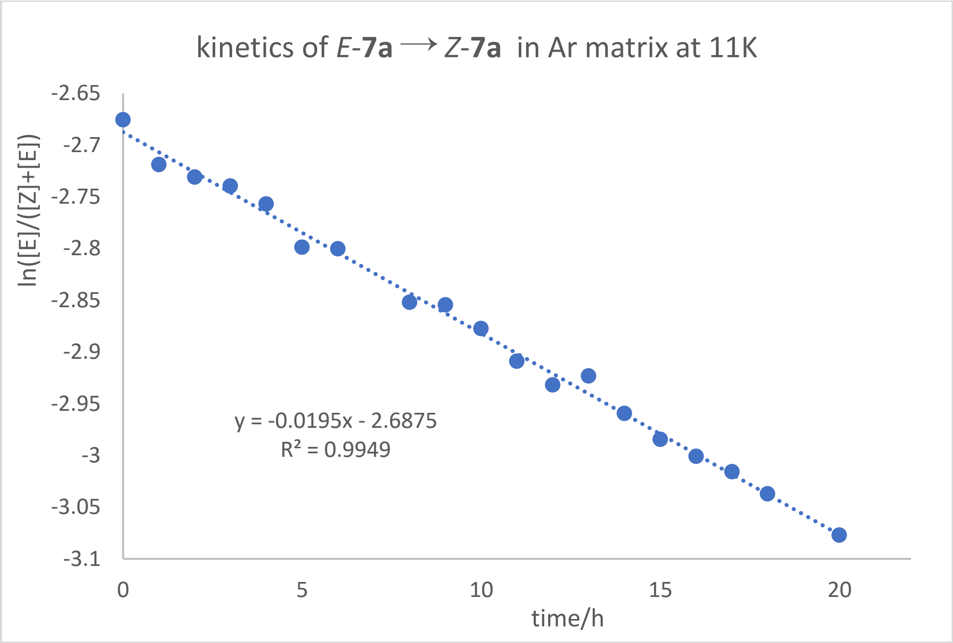

Figure S5.3. Kinetic evaluation of *E*-**7a** (1800.8 cm^{-1}) ⟶ *Z*-**7a** (1779.0 cm^{-1}) in Ar, 11 K; half-life $t_{1/2(exp)}$ (**7a**) = (2080 ± 50) min.

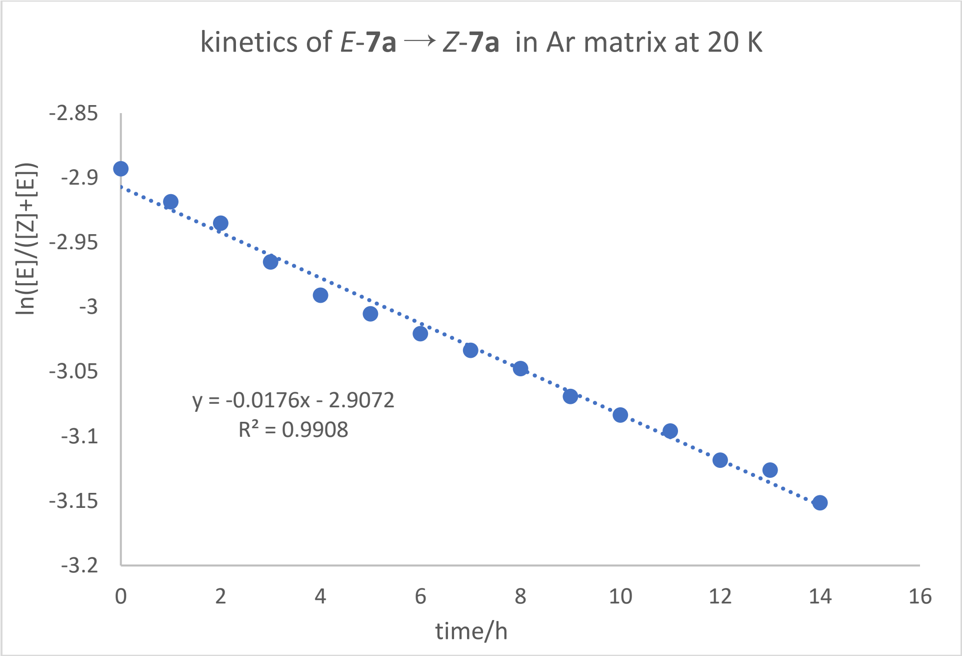

Figure S5.4 Kinetic evaluation of *E*-**7a** (1800.8 cm^{-1}) ⟶ *Z*-**7a** (1779.0 cm^{-1}) in Ar, 20 K; half-life $t_{1/2(exp)}$ (**7a**) = (2360 ± 60) min.

The kinetic behavior of the **6b**, **6c**, and **6e** isomerizations is more complicated, because the rate constant *k* measured in a given matrix at 11 K is not constant, but displayed two rate constants. This is observed frequently in solid-state reactions resulting in dispersive kinetics. Some molecules form in “fast” matrix sites and isomerize much more quickly than others that occupy “slow” sites, where “fast” sites react first and “slow” sites later.[4] We employ the model (**equation 1**) from our previous study of dispersive kinetics in conformational tunneling in carbonic acid monomethyl ester.[5] As before, we define the effective half life as the time required for the fitted relative population function of the *E*-isomer to reach $[Y]=1/2[Y]_0$.

$$Y/Y_0 = \frac{2^{-t/\tau_{slow}} + B \times 2^{-t/\tau_{fast}}}{1 + B} \qquad (1)$$

Y: percentage of *E*-isomer [E]/([E]+[Z])

Y_0: initial percentage of *E*-isomer $[E]_0/([E]_0+[Z]_0)$

B: $[E]_{fast\text{-}site}/[E]_{slow\text{-}site}$

τ_{slow}: half life of the slow-site

τ_{fast}: half life of the fast-site

effective half life: time required to reach $[Y]=1/2[Y]_0$

kinetics of *E*-(**6b**) → *Z*-(**6b**) in Ar matrix at 11 K

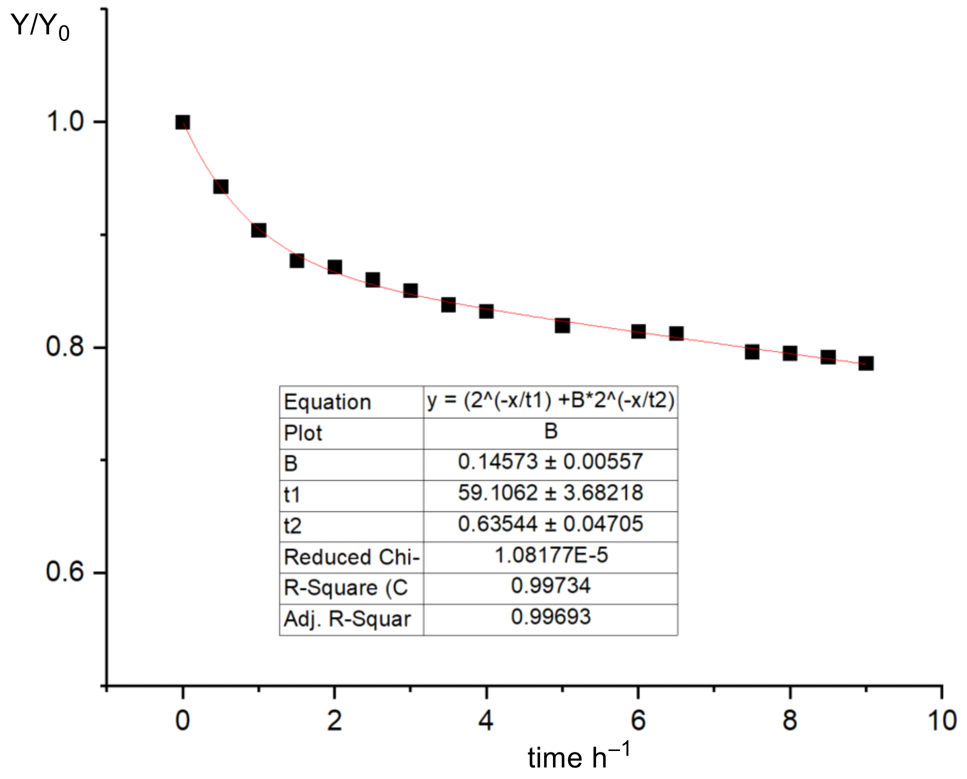

Figure S5.5 Kinetic evaluation of *E*-(**6b**) (1786.5 cm^{-1}) → *Z*-(**6b**) (1747.5 cm^{-1}) in Ar, 11 K

k_{fast} = (0.087 ± 0.009) h^{-1}, % $_{fast\text{-}site}$ = 12%
k_{slow} = (0.0137 ± 0.0008) h^{-1} , % $_{slow\text{-}site}$ = 88%
half-life $t_{1/2(exp)}$ (**6b**) = (2800 ± 100) min

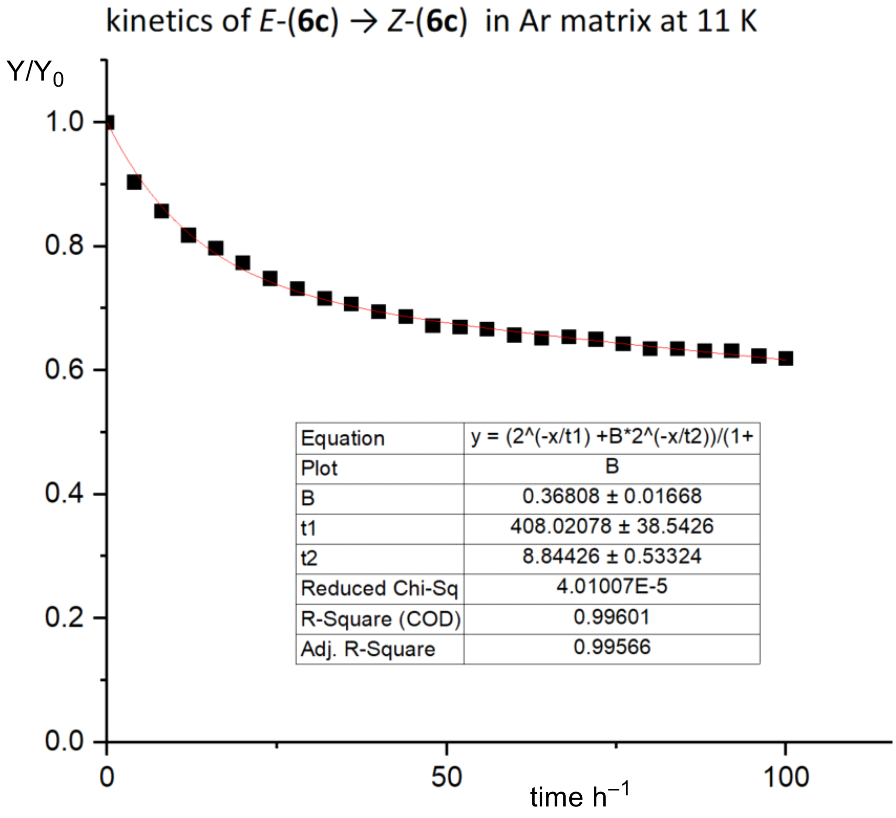

Figure S5.6. Kinetic evaluation of *E*-(**6c**) (1780.8 cm^{-1}) → *Z*-(**6c**) (1741.5 cm^{-1}) in Ar, 11 K

k_{fast} = (0.0081 ± 0.0005) h^{-1}, % $_{fast\text{-}site}$ = 30%
k_{slow} = (0.0018 ± 0.00009) h^{-1} , % $_{slow\text{-}site}$ = 70%
half-life $t_{1/2(exp)}$ (**6c**) = (13300 ± 700) min

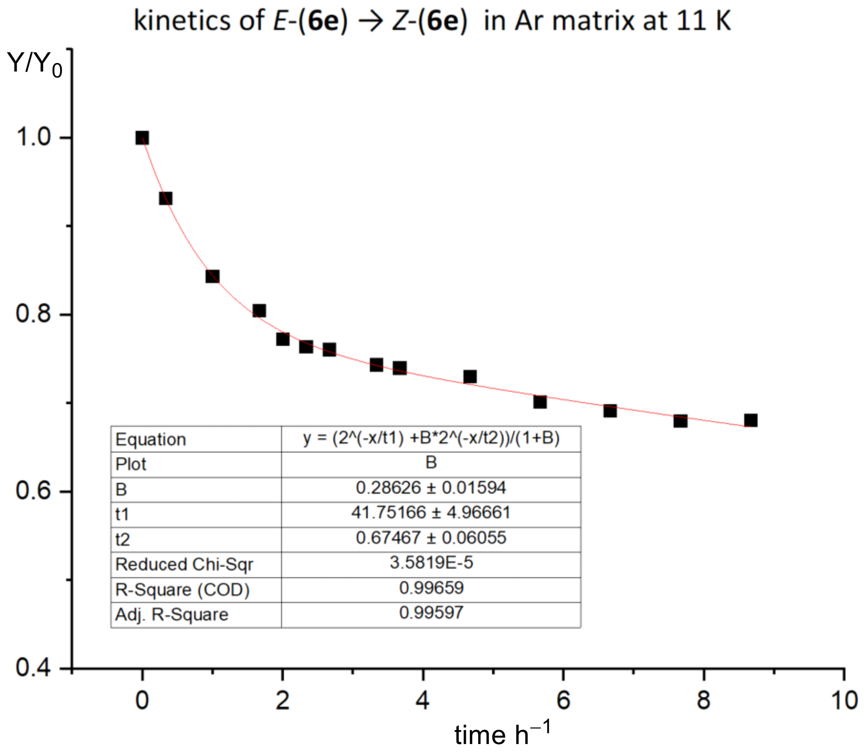

Figure S5.7. Kinetic evaluation of *E*-(**6e**) (1788.7 cm^{-1}) ⟶ *Z*-(**6e**) (1751.4 cm^{-1}) in Ar, 11 K

k_{fast} = (0.11 ± 0.01) h^{-1}, % $_{fast\text{-}site}$ = 23%
k_{slow} = (0.022 ± 0.001) h^{-1}, % $_{slow\text{-}site}$ = 77%
half-life $t_{1/2(exp)}$ (**6e**) = 30.8h = (1600 ± 200) min

As mentioned in the main text, to provide a more intuitive sense of the significance of the intrinsic QMT reactivity, we take the *ortho*-H series as the reference and assume that all compounds studied have the same intrinsic QMT reactivity and that the BEP principle alone is able to model their reactivity. Each reaction's free activation energy change was submitted to the linear BEP equation of the *ortho*-H series, obtaining $t_{1/2(\text{“R=H”})}$ in Table 2 of the main text. Such hypothetical half-lives are about two orders of magnitude different from the experimental half-lives. Here we compare each of the fast site and the slow site individually with $t_{1/2(\text{“R=H”})}$ (**Table S5.1**). All $t_{1/2(\text{“R=H”})}$ differ from the actual half-lives of the fast site and the slow site by about 1.5-3.5 orders of magnitude, both still manifesting the importance of intrinsic QMT reactivity.

Table S5.1. Experimental half-lives of the fast site ($t_{1/2(fast)}$) and the slow site ($t_{1/2(slow)}$) for the $E \longrightarrow Z$ rotamerization of compounds **6b**, **6c**, and **6e** in Ar matrix at 11 K, and hypothetical half-lives ($t_{1/2''R=H''}$) that assume that the intrinsic reactivity for all compounds (regardless of R) is identical to the R=H series.

Compound	R=	X=	Δ*G* (kcal/mol)	$t_{1/2(fast)}$ (min)	$t_{1/2(slow)}$ (min)	$t_{1/2''R=H''}$ (min)
**6b**	Me	Me	−4.1	530	24500	517000
**6e**	Me	Cl	−4.4	40	2500	132000
**6c**	Me	F	−4.4	40	3500	150000

The rate constant of a QMT reaction not only depends linearly on the barrier width but also has a square root dependence on the barrier height, by our definition. Thus, the *intrinsic* QMT reactivity also has a square root dependence on the *intrinsic* barrier height, besides the linear dependence on the *intrinsic* barrier width. The plot of experimental half-life against thermodynamic driving force is normalized to remove the effect of the barrier height to the QMT rate (**Figure S5.8**). There are still three distinct intrinsic QMT reactivities displayed, reflecting exclusively the role of the intrinsic barrier width.

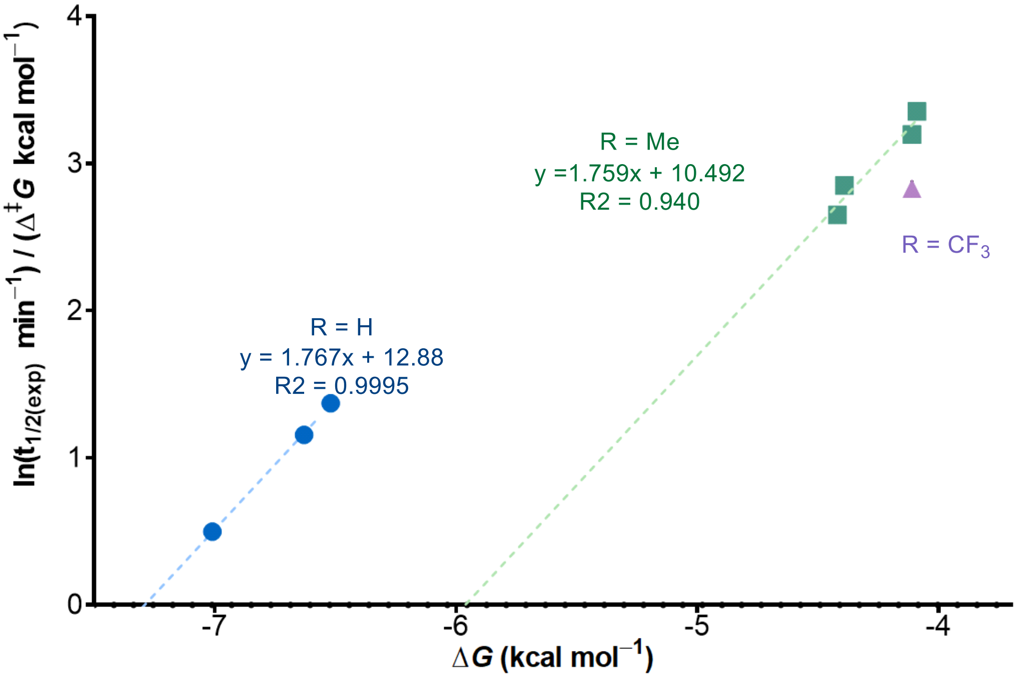

Figure S5.8. Barrier-height-normalized plot of experimental QMT ln($t_{1/2}$) in min against the free energy change of $E \longrightarrow Z$ rotamerization in Ar matrix at 11 K.

References

(1) Gaussian 09, M. J. Frisch, G. W. Trucks, H. B. Schlegel, G. E. Scuseria, M. A. Robb, J. R. Cheeseman, G. Scalmani, V. Barone, B. Mennucci, G. A. Petersson, H. Nakatsuji, M. Caricato, X. Li, H. P. Hratchian, A. F. Izmaylov, J. Bloino, G. Zheng, J. L. Sonnenberg, M. Hada, M. Ehara, K. Toyota, R. Fukuda, J. Hasegawa, M. Ishida, T. Nakajima, Y. Honda, O. Kitao, H. Nakai, T. Vreven, J. A. Montgomery, Jr., J. E. Peralta, F. Ogliaro, M. Bearpark, J. J. Heyd, E. Brothers, K. N. Kudin, V. N. Staroverov, R. Kobayashi, J. Normand, K. Raghavachari, A. Rendell, J. C. Burant, S. S. Iyengar, J. Tomasi, M. Cossi, N. Rega, J. M. Millam, M. Klene, J. E. Knox, J. B. Cross, V. Bakken, C. Adamo, J. Jaramillo, R. Gomperts, R. E. Stratmann, O. Yazyev, A. J. Austin, R. Cammi, C. Pomelli, J. W. Ochterski, R. L. Martin, K. Morokuma, V. G. Zakrzewski, G. A. Voth, P. Salvador, J. J. Dannenberg, S. Dapprich, A. D. Daniels, O. Farkas, J. B. Foresman, J. V. Ortiz, J. Cioslowski, D. J. Fox, Gaussian, Inc., Wallingford CT, 2009

(2) Amiri, S., Reisenauer, H. P. & Schreiner, P. R. Electronic effects on atom tunneling: Conformational isomerization of monomeric para -substituted benzoic acid derivatives. *J. Am. Chem. Soc.* **132**, 15902–15904 (2010).

(3) J. Zheng, J. L. Bao, R. Meana-Pañeda, S. Zhang, B. J. Lynch, J. C. Corchado, Y.-Y. Chuang, P. L. Fast, W.-P. Hu, Y.-P. Liu, G. C. Lynch, K. A. Nguyen, C. F. Jackels, A. Fernandez Ramos, B. A. Ellingson, V. S. Melissas, J. Villà, I. Rossi, E. L. Coitiño, J. Pu, T. V. Albu, A. Ratkiewicz, R. Steckler, B. C. Garrett, A. D. Isaacson, D. G. Truhlar, Polyrate Version 2017-C, 2017, University of Minnesota, Minneapolis.

(4) Ertelt, M., Hrovat, D.A., Borden, W.T. & Sander, W. Heavy-Atom Tunneling in the Ring Opening of a Strained Cyclopropene at Very Low Temperatures. *Chem. Eur. J.* **20**, 4713-4720 (2014).

(5) Linden, M. M.; Wagner, J. P.; Bernhardt, B.; Bartlett, M. A.; Allen, W. D.; Schreiner, P. R. Intricate Conformational Tunneling in Carbonic Acid Monomethyl Ester. **9** *J. Phys. Chem. Lett.* 1663– 1667 (2018)
