# Supplementary material for: The Intrinsic Barrier Width and Its Role in Chemical Reactivity
Source: ACS Cent Sci. 2023 Nov 6;9(11):2129–37. doi: 10.1021/acscentsci.3c00926 (PMC10683502; doi:10.1021/acscentsci.3c00926)
Supplement: Supplementary file 2 — oc3c00926_si_002.pdf [file oc3c00926_si_002.pdf]

oc-2023-009267.R1

Name: Peer Review Information for "The Intrinsic Barrier Width and its Role in Chemical Reactivity"

First Round of Reviewer Comments

Reviewer: 1

Comments to the Author

Brilliant work. This is the best paper that I have reviewed in 5 years.

A unified theory is presented based on Marcus-type analysis of barrier widths, which is missing from the mechanistic picture in contemporary thoughts of potential energy surfaces. Barrier "heights" have been studied extensively, but almost no studies exist on barrier "widths", especially as a unifying theory as is presented here. This can certainly provide a paradigm shift to organic chemistry. How this was assessed by both theoretical and experimental methods on E/Z isomerization of para-substituted (electronic) and ortho-substituted (sterically hindered) benzoic acids is not only convincing, it is ingenious.

The graphics that were presented helped to guide the reader grasp a lot of information quickly, some of it is quite complicated. I think the work opens the door and represents the start of many systems that can now be tested. There are now many new vistas to explore, I have no recommended changes to the manuscript except for an optional/forward-looking comment on implications to photochemical (excited-state) processes with return to the ground state and how barrier width on the ground state and near the conical intersection may play a role in reaching high-energy products that is yet to be explored (although this kind of speculative comment may be premature at this moment).

I recommend the manuscript is published as is.

Reviewer: 2

Comments to the Author

This is a very interesting and well-written contribution. I recommend publication essentially as is, except for a few minor language fixes:

* both "tunnelling" and "tunneling" (the more usual US English spelling) are used in the MS. Even if one insists on the double "l", one should be consistent about it

* p.1 RH column: "in order acquire" there is a "to" missing there

* caption of Figure 3 is a little confusing: "i, iii, and v have the same IBW, whereas ii, iv, and vi has the same IBW". I would rephrase the second instance to something like "... share a different IBW" or "whereas ii, iv, and vi all have a different IBW in common".

* Ref. 46: Marti should read Martin

Reviewer: 3

Comments to the Author

This paper presents a neat idea, but I believe it falls short to establish something new in the field. The point of the separability of the components of QMT, namely barrier height, width and mass as done for the isomerization of benzoic acid derivatives is ingenious and it is well developed. It is also productive to establish the importance of what here is called the “intrinsic barrier width”.

However, from a physical chemistry viewpoint the concepts are already widely known in the field. When reading the introduction and the citation of Marcus theory, I thought the article will provide a mathematical derivation of the influences of the driving force and frequencies on the barrier width (as Marcus did on the barrier heights). But the article only deals with qualitative points on the shape of the barrier that appear on many articles and books. Therefore, I believe that the article will be of practical use only if a more profound model of the barrier is brought.

In addition, the intrinsic barrier width at the base of the reactant state is important to describe the reaction, but in terms of the probability of tunnelling the barrier width is variable along the reaction, and it is more important with higher energies. If the barrier is very narrow at the top (high imaginary frequency) but with a large intrinsic barrier width at the bottom, the reaction can still occur by QMT. This cannot be analysed when comparing only one reaction.

A couple of small other issues:

It took me some time to understand the definition of intrinsic barrier as written in page 2, line 20. Maybe instead of saying “at zero driving force” it can say “at the same energy of the reactant”?

Speaking of a “unified theory” sound like a big overstatement.

I would change the letter omega with w for the barrier width, since the former is used for frequencies.

In the caption of fig.2, instead of “intrinsic barrier reflects” it should be “intrinsic barrier width reflects”.

The QMT method is not correctly explain in the text.

The mass weighted coordinates are used as a measure of the barrier widths. This is not correct. While mass weighted coordinates are useful, the proper width should be in units of distance.

I think I understand the idea of fig. 8, but the explanation in the caption and in the main text is extremely confusing.

Reviewer: 4

Comments to the Author

The authors present a combined experimental/theory based proposal for the new concept of a Marcus type dissection of the barrier widths

of a chemical reaction into a thermodynamic-independent (intrinsic) and the thermodynamic-dependent (Bell-Evans-Polanyi) parts. The

conclusions are important and generally relevant for the tunneling contribution to chemical reaction rates.

These observed disparities between experimental and theoretical values are explained by solvent effects of the Ar matrix host.

The study is carefully and competendly executed with reasonable computational chemistry methods. In my opinion an original

theoretical ansatz is suggested which is fully supported by the presented data. The question how to control barrier

widths practically remains open and is suggested as a challenge for the chemical community.

The MS is of high overall quality and is publishable basically as is.

Author's Response to Peer Review Comments:

Prof. Dr. Peter R. Schreiner, PhD
Liebig Chair, ML, FRSC

Heinrich-Buff-Ring 17, 35392 Giessen, Germany Fon: +49 641 99-34300 | Fax: +49 641 99-34309 prs@uni-giessen.de www.uni-giessen.de/schreiner

September 29, 2023

Revision of oc-2023-009267

Dear Prof.

Thank you for your good mail of September 19 requesting a revision of the above manuscript. We found the reviewer’s comments to be thoughtful and constructive and we believe that the changes we have made to address their concerns have improved the quality and clarity of our paper. Our point-by-point responses (as well as descriptions of the associated changes to the manuscript) are provided below.

Reviewer 1

I have no recommended changes to the manuscript except for an optional/forward-looking comment on implications to photochemical (excited-state) processes with return to the ground state and how barrier width on the ground state and near the conical intersection may play a role in reaching high-energy products that is yet to be explored (although this kind of speculative comment may be premature at this moment).

RESPONSE: We agree that commenting on the implication to excited-state photochemical processes is indeed thought-provoking. As the reviewer mentioned, it is too early to specify the concrete implications. On the general side, the concept of intrinsic barrier width is relevant to all types of reactions (besides photochemical processes) where the barrier shape matters.This, however, is a large body of work for the future.

Reviewer 2

This is a very interesting and well-written contribution. I recommend publication essentially as is, except for a few minor language fixes:

** both "tunnelling" and "tunneling" (the more usual US English spelling) are used in the MS. Even if one insists on the double "l", one should be consistent about it*

RESPONSE: We use "tunneling“ consistently throughout the main text. "Tunnelling“ was only used in references, where we chose to keep the original spelling of the given titles.

** p.1 RH column: "in order acquire" there is a "to" missing there*

RESPONSE: Corrected.

** caption of Figure 3 is a little confusing: "i, iii, and v have the same IBW, whereas ii, iv, and vi hasthe same IBW". I would rephrase the second instance to something like "... share a different IBW" or "whereas ii, iv, and vi all have a different IBW in common".*

RESPONSE: Corrected.

Reviewer 3
This paper presents a neat idea, but I believe it falls short to establish something new in the field. The point of the separability of the components of QMT, namely barrier height, width and mass as done for the isomerization of benzoic acid derivatives is ingenious and it is well developed. It is also productive to establish the importance of what here is called the "intrinsic barrier width". However, from a physical chemistry viewpoint the concepts are already widely known in the field. When reading the introduction and the citation of Marcus theory, I thought the article will provide a mathematical derivation of the influences of the driving force and frequencies on the barrier width (as Marcus did on the barrier heights). But the article only deals with qualitative points on the shape of the barrier that appear on many articles and books. Therefore, I believe that the article will be of practical use only if a more profound model of the barrier is brought.

RESPONSE: We respectfully but firmly disagree with the reviewer's first sentence in the absence of a single reference that would point us to the formulation of the qualitative description of barrier width as it is done in our present work. We recognize the anticipation for a mathematical derivation, a pursuit planned for our future work. Here, we focus on establishing the novelty of explicitly conceptualizing and separating the intrinsic barrier width and the thermodynamic effect on the barrier, a perspective not previously articulated. These insights set the stage for more detailed mathematical models in subsequent studies.

The Marcus dissection of the barrier height led to numerous flavors of semi-quantitative linear free energy relationship (LFER) correlations that have provided many valuable insights and have allowed reaction prediction. Regarding the barrier width, only the Bell-Evan-Polanyi (BEP) aspect (i.e., perceiving the intrinsic component as constant whereas driving force is the only independent variable) has been briefly studied. We firmly believe that work is the first to conceptualize the "barrier-width-counterparts" of the intrinsic barrier and Brønsted slope, bringing attention to the intrinsic barrier width as a variable alongside the BEP effect. While the absence of a mathematical model may seem like a limitation, our qualitative conceptual groundwork is a foundational step, deserving of a dedicated paper.

In fact, there are many examples reporting that, while QMT reactivity is strongly affected by parameters like thermodynamic driving force, substituents, and matrix environments, the trends are often conflicting. We present the following two examples to show how our new concept can help systematically resolve these conflicting trends:

(1) (Org. Lett. **2010**, *12*, 2798)

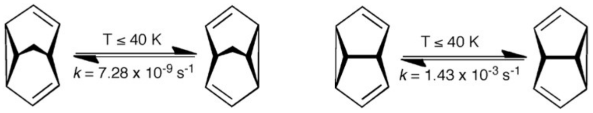

The two identity reactions drastically differ in computational QMT rate constants. The driving force of both reactions is zero. Thus, the drastic difference in the QMT reactivity manifests the

…/3

difference in the intrinsic barrier width. No such proposal has been made in the paper or in subsequent work.

(2) (*Chem.Eur. J.* **2020**, *26*, 10452)

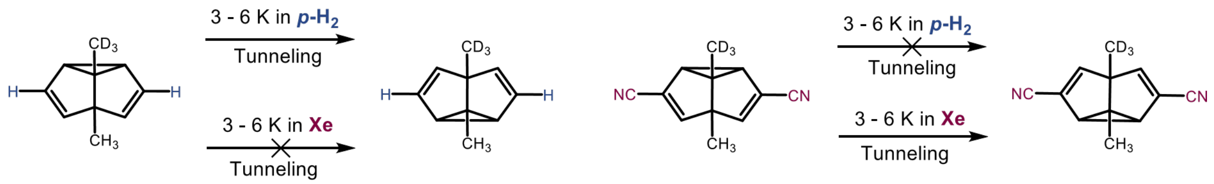

With different substituents (H or CN), the tunneling reactivity trends in different solvents are opposite. We propose that the solvation effect on the barrier consist both the intrinsic (left) and the thermodynamic (right) components:

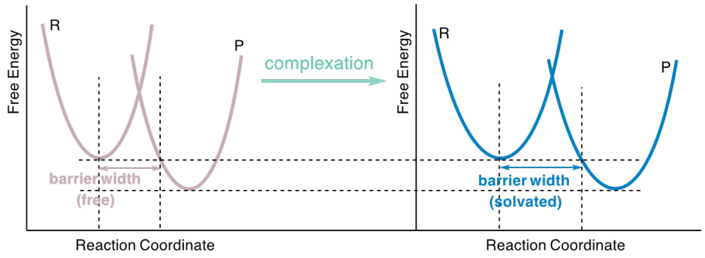

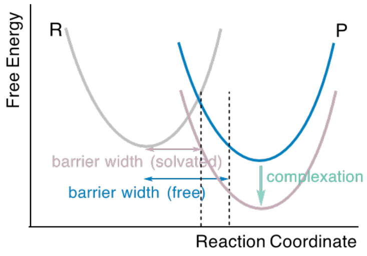

The interplay between the two components could result in one reaction series being relatively more reactive at higher driving forces while the other reaction series is relatively more reactive at lower driving forces:

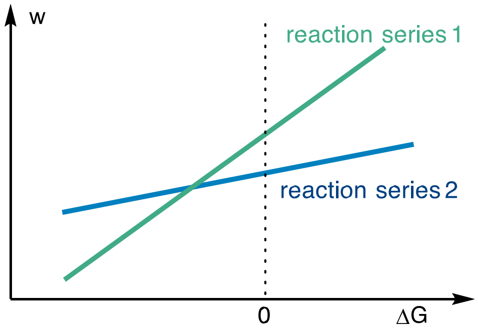

With the new concept we formulated, many observed QMT reactivity trends may no longer seem paradoxical and we would be able to come up with chemically intuitive ways to change the QMT reactivity and selectivity. In the synthetic organic chemistry community, even the intrinsic barrier height is underappreciated and the BEP effect has been the main consideration in many reaction designs. Therefore, we believe that a (less abstract) paper about qualitative understanding about the Marcus dissection of barrier width is necessary prior to the mathematical derivation.

With the reasons above, we believe that a qualitative conceptual formulation is necessary for the community to raise the awareness of the interplay between intrinsic and thermodynamic effects on the barrier width. At the same time, we look forward to the mathematical derivation in the next phase.

In addition, the intrinsic barrier width at the base of the reactant state is important to describe the reaction, but in terms of the probability of tunnelling the barrier width is variable along the reaction, and it is more important with higher energies. If the barrier is very narrow at the top (high imaginary frequency) but with a large intrinsic barrier width at the bottom, the reaction can still occur by QMT. This cannot be analysed when comparing only one reaction.

RESPONSE: We appreciate the reviewer's comment. "Intrinsic" refers to the thermodynamicindependent aspects. The Marcus thermodynamic effect vertically shifts all vibrational states. Therefore, every vibrational level of the reactant state has its own intrinsic barrier width and sensitivity of change in width in response to change in driving force (the "barrier-width-counterpart" of the Brønsted slope). At the bottom, the intrinsic barrier width is the barrier width at the vibrational ground state at zero thermodynamic driving force. Near the barrier top, the intrinsic barrier width is the barrier width at the higher vibrational state at zero thermodynamic driving force. As the first paper of the concept of intrinsic barrier width, we chose a reaction at cryogenic temperatures for simplicity. Thus, the intrinsic barrier width at the vibrational ground state is sufficiently representative. We use the following figure to compare and contrast the intrinsic barrier width's implications on the effects of thermodynamic driving force and vibrational excitation:

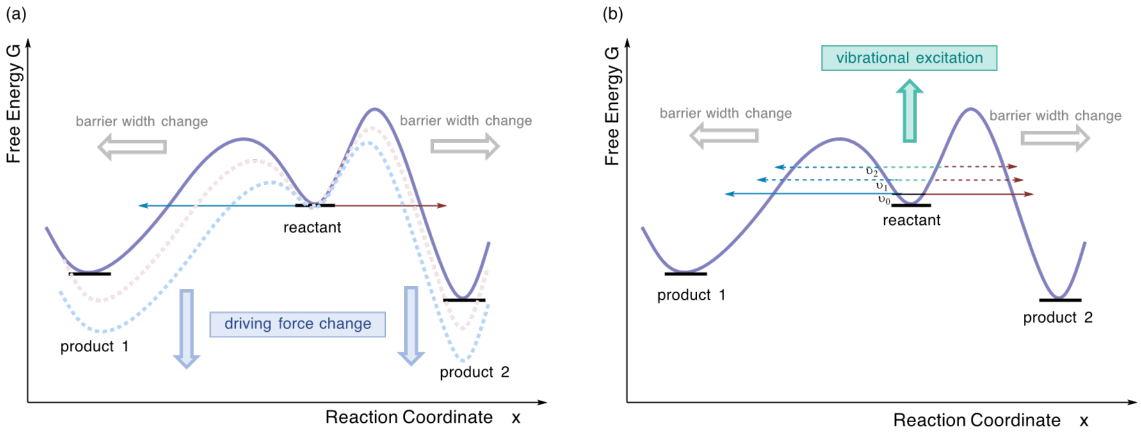

Despite the width change in both scenarios being directly affected by intrinsic barrier width, the thermodynamic BEP effect and the vibrational energy excitation are not the same. The former is the change in the whole set of all vibrational levels' energies represented by vertical displacement of the IRC-related Marcus parabolae without any change in the population partitioned among the vibrational energy levels (a), whereas the latter is the change in the population partitioned among the vibrational energy levels without any vertical displacement of the IRC-related Marcus parabola (b). In this study, (a) is the representative scenario, as the reactions were performed at cryogenic temperatures where only the vibrational ground state is populated, and the thermodynamic free energy change of the reactions was varied.

Nevertheless, understanding the effect of intrinsic barrier width will also provide useful insights and guidance for better control of (e.g., thermally- and photochemically-activated) QMTs near the barrier top.

A couple of small other issues:
It took me some time to understand the definition of intrinsic barrier as written in page 2, line 20. Maybe instead of saying "at zero driving force" it can say "at the same energy of the reactant"?

RESPONSE: We would like to retain the definition, as "at zero driving force“ the barrier width reflects the intercept of the rate-driving force correlation, which points to "intrinsic”.

Speaking of a “unified theory” sound like a big overstatement.

RESPONSE: We have changed “unified theory” to “unified paradigm”. We would like to retain the word “unified”, as the unification of the “intrinsic and thermodynamic components” of the “barrier

…/5

height and width” are unprecedented. This is the way of thinking we would like to introduce to the practical chemistry community.

I would change the letter omega with w for the barrier width, since the former is used for frequencies.

RESPONSE: Done.

In the caption of fig.2, instead of “intrinsic barrier reflects” it should be “intrinsic barrier width reflects”.

RESPONSE: Corrected.

The mass weighted coordinates are used as a measure of the barrier widths. This is not correct. While mass weighted coordinates are useful, the proper width should be in units of distance.

RESPONSE: As the reviewer mentioned, this paper is a qualitative formulation of a new concept, in which one of our main goals is to make chemists rethink reaction design. To the end, we select the unit of the barrier width that is more useful in rationalizing reactivity and selectivity, in reactions such as tunneling, post transition state bifurcation, and non-statistical internal vibrational energy redistribution, which are directly related to momenta of atoms (thus mass). Therefore, we would like to retain the mass-weighted coordinates and this the units.

I think I understand the idea of fig. 8, but the explanation in the caption and in the main text is extremely confusing.

RESPONSE: More elaborations have been added in the caption and in the main text.

Reviewer 4

No change is requested

Formatting Needs:

AU EMAIL: Please include the email address of the corresponding author on the first page of the manuscript, and the Supporting Information if submitted, with an asterisk next to their name in the author list. Please be sure to label “email.”

RESPONSE: The email addresses of the corresponding authors have been added on the first page of the manuscript and the supporting information. The asterisk and the email addresses are next to the names of the corresponding authors as it is in the “Corresponding Authors“ section. *ABSTRACT: Please remove figure*

RESPONSE: Done.

SI PARAGRAPH: If the manuscript is accompanied by any supporting information for publication, a brief description of the supplementary material is required in the manuscript. The appropriate format is: Supporting Information. Brief statement in non-sentence format listing the contents of the material supplied as Supporting Information.

…/6

RESPONSE: Done.

GENERAL REF FORMATTING: Periodical references should contain authors’ surnames followed by initials, article title, journal abbreviation, year, volume number, and page range. Refs with more than 10 authors should list the first 10 and then be followed by “et al.”

RESPONSE: We believe that we have met these requirements.

Web sources must include access date.

RESPONSE: Done.

In closing, I hope you find these comments and changes adequately address all of the reviewers’ concerns and hope for your favorable consideration.

Sincerely yours,

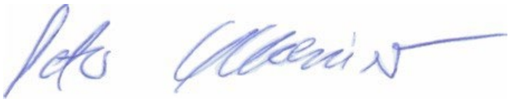

Prof. Dr. Peter R. Schreiner
